# Supplementary material for: Primed histone demethylation regulates shoot regenerative competency
Source: Nat Commun. 2019 Apr 16;10:1786. doi: 10.1038/s41467-019-09386-5 (PMC6467990; doi:10.1038/s41467-019-09386-5)
Supplement: Supplementary file 1 — Supplementary Information [file 41467_2019_9386_MOESM1_ESM.pdf]

Supplementary Information

Primed histone demethylation regulates shoot regenerative competency

Hiroya Ishihara, Kaoru Sugimoto, Paul Tarr, Haruka Temman, Satoshi Kadokura, Yayoi Inui, Takuya Sakamoto, Taku Sasaki, Mitsuhiro Aida, Takamasa Suzuki, Soichi Inagaki, Kengo Morohashi, Motoaki Seki, Tetsuji Kakutani, Elliot M. Meyerowitz, and Sachihiro Matsunaga.

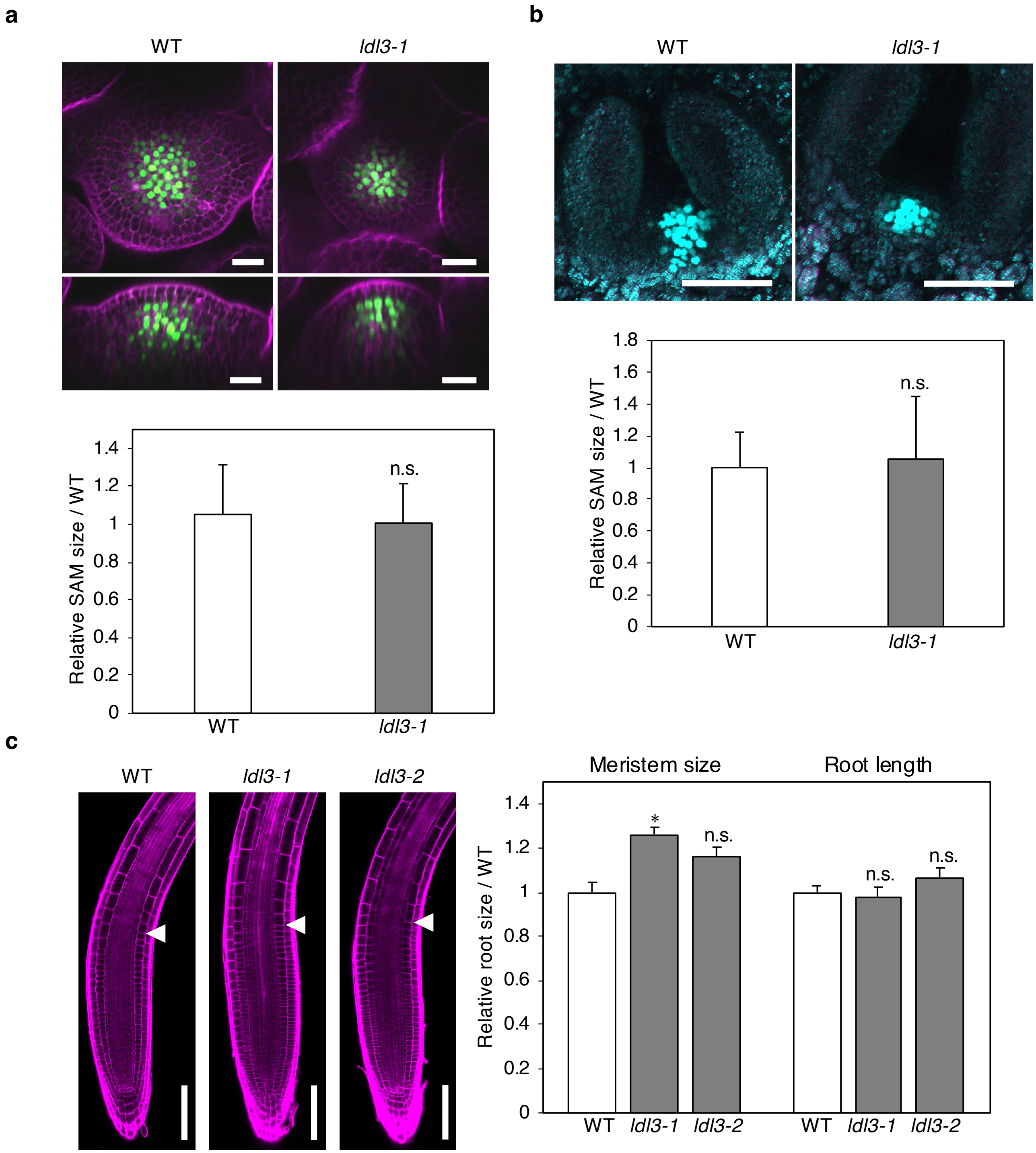

Supplementary Fig. 1. Phenotypes in shoot and root meristems in WT and *ldl3*.
a, **b** The organizing center of the SAM visualized with *pWUS::mTq2* (green) in plants after bolting (**a**) and in seedlings 3 days after sowing (**b**). The number of cells marked by *WUS* reporter with a nuclear localization signal was counted as the size of SAM. Spot signals with 3.5 – 5.0 µm in diameter in the SAM were extracted and counted after 3D construction of confocal Z-stack images using Imaris software. **c** Root meristems in seedlings 5 days after germination. Arrowheads indicate the top of the RAM region (length/width of cortex cells < 1). The total length of cortex cell files in the RAM region was measured as the size of the RAM. The root length of each line of explants was also measured at the same stage. Cellular outlines were visualized with PI staining (magenta) (**a** and **c**). All images are single optical sections. Values are mean ± s.d., n.s., not significant, *: $p < 0.05$ (Student's *t*-test). $12 < n < 20$ (**a**), $n = 23$ (**b**), and $n > 77$ (**c**). Scale bars: 20 µm (**a**), 50 µm (**b**), and 100 µm (**c**). Source data are provided as a Source Data file.

a

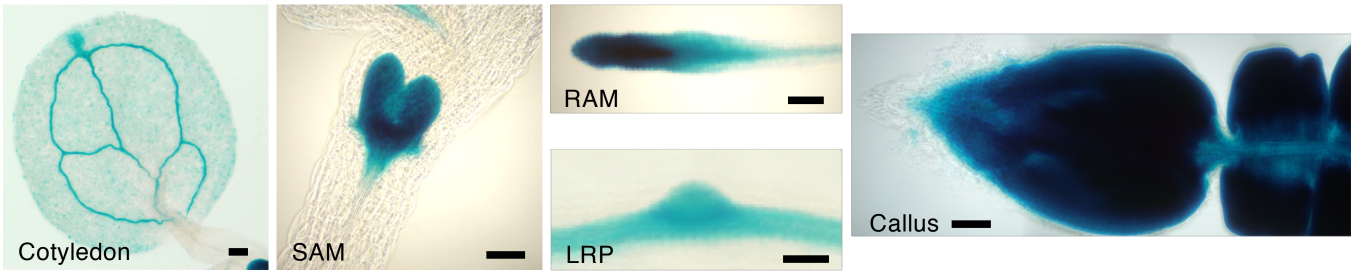

b

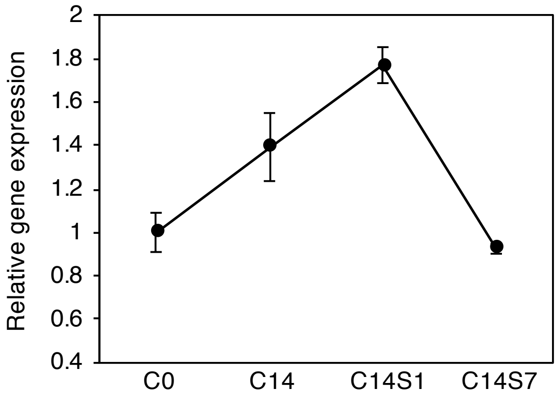

Supplementary Fig. 2. LDL3 expression patterns in seedlings and calli.

a Expression pattern of the *LDL3* transcriptional reporter (GUS) in seedlings (6 days after germination) and calli (CIM 14 days). Scale bars: 100 µm. **b** Expression level of *LDL3* in the root explants detected by RNA-seq at CIM 0, 14 days and SIM 1, 7 days (C0, C14, C14S1, and C14S7). Values are mean ±s.e. (n = 3). Source data are provided as a Source Data file.

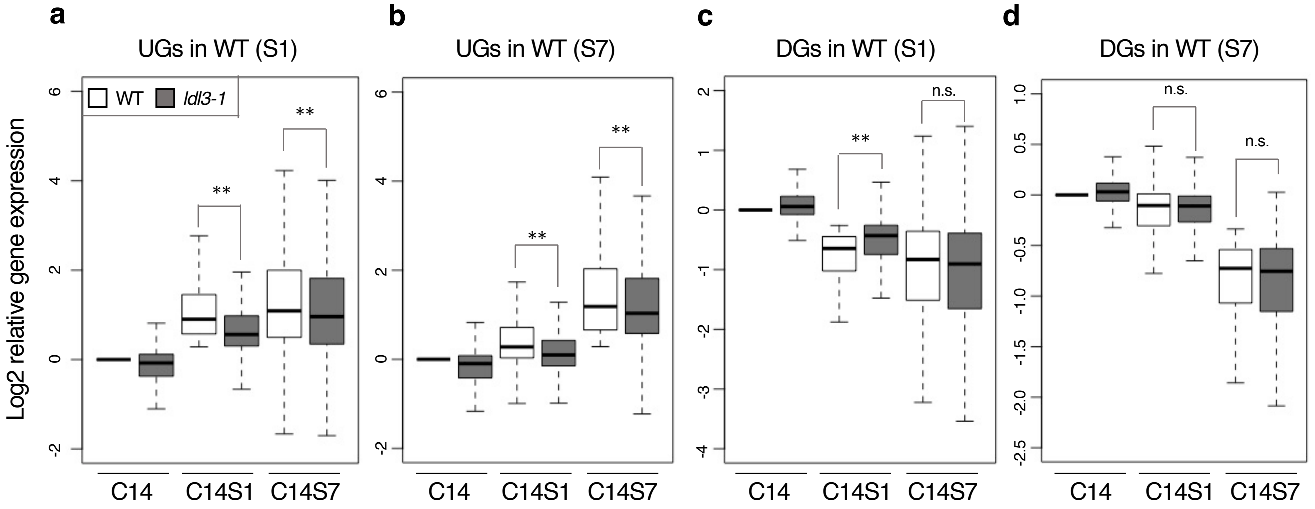

Supplementary Fig. 3. Expression changes of the genes up- or down-regulated in response to shoot induction at early or late stages in wild-type.

Comparing transcriptome profiles of wild-type (WT) before and after shoot induction (C14 vs C14S1 or C14 vs C14S7) identified genes that are up- or down-regulated (FC > 1.25 or < 0.8, $p < 0.01$) in response to shoot induction at early or late stages in WT (UGs_S1, DGs_S1, UGs_S7, and DGs_S7). The box plot shows the relative expression levels (Log2 ldl3/WT) of UGs_S1 (**a**), UGs_S7 (**b**), DGs_S1 (**c**), and DGs_S7 (**d**) at C14, C14S1 and C14S7, in WT and ldl3 (compared with those at C14 in WT). **, $p < 0.01$; n.s., not significant, $p > 0.05$ (Wilcoxon rank sum test).

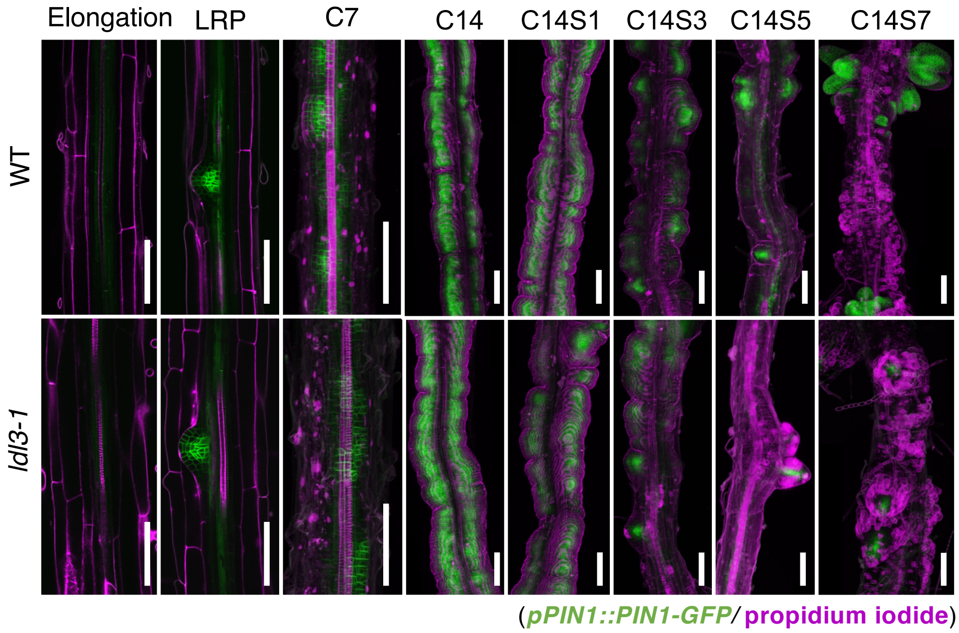

Supplementary Fig. 4. Expression pattern of auxin efflux carrier during shoot regeneration. Expression patterns of *PIN-FORMED 1* (*PIN1*) reporter, *pPIN1::PIN1-GFP* (green) in WT and *ldl3* explants. All panels are combined images of three sequential projections. Cellular outlines (magenta) were visualized with PI staining. Scale bars: 100 μm. There were no differences between wild-type and *ldl3* explants in their tissue morphology and reporter expression patterns until C14S1. At C14S3 and C14S5, the reporter signal localized in the LRP-like structures in the mutant explants, while it was detected in the shoot progenitor cells in wild-type explants. The mutant explants formed more roots than wild-type explants (C14S7).

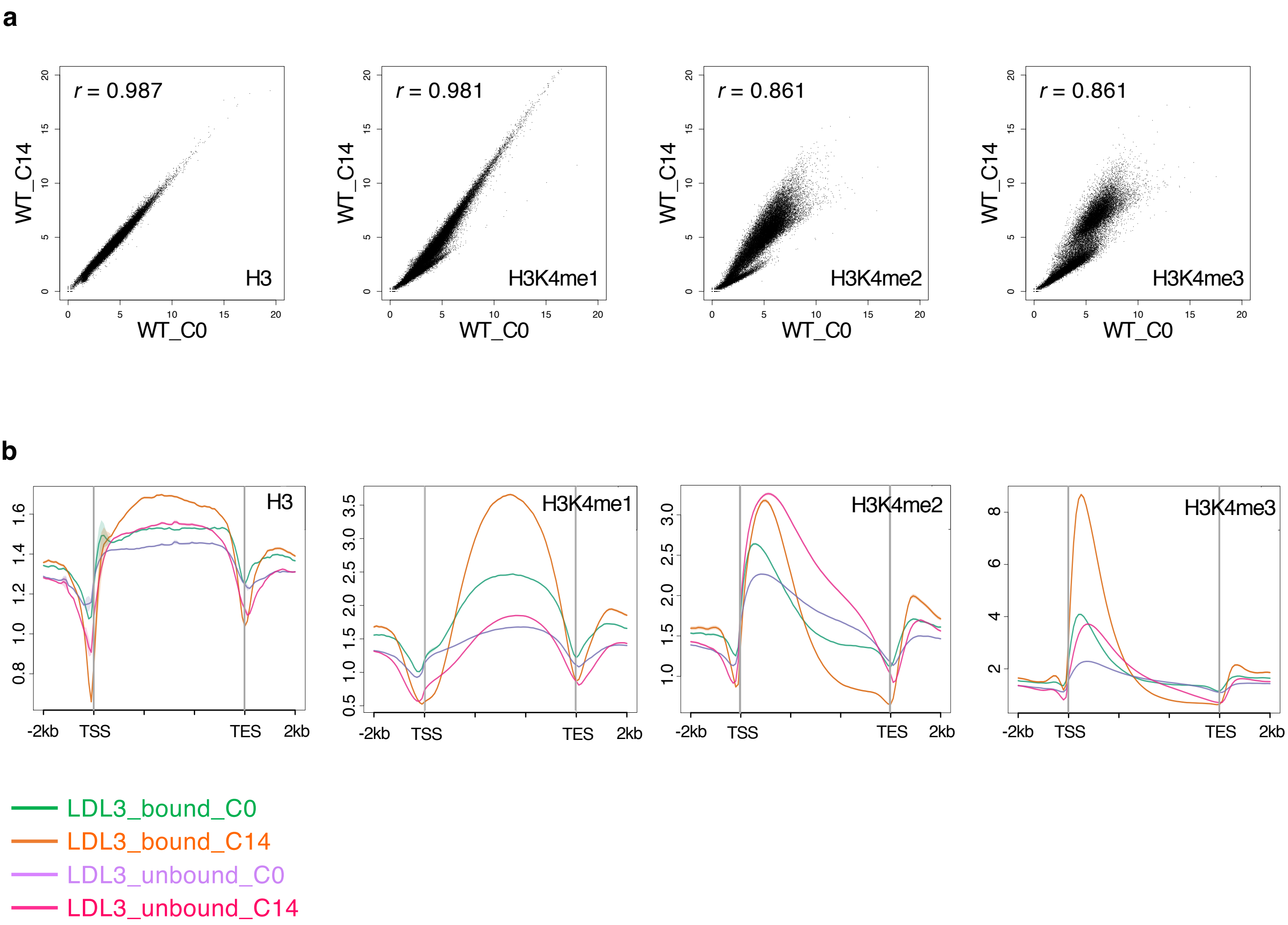

Supplementary Fig. 5. H3K4 methylation levels in WT during callus formation.
a H3 and H3K4 methylation levels in WT calli (C14) compared with original tissue (C0). Each dot represents the square root of RPM. Pearson correlation coefficient (*r*) is shown in each panel. H3K4me2 and H3K4me3 were enhanced during callus formation in WT explants. **b** Positional profiles of H3K4 methylation on LDL3-bound and -unbound genes in WT explants before (C0) and after callus induction (C14). H3, H3K4 mono- and tri-methylation increased in LDL3 bound genes during callus formation in WT explants, while only H3K4me2 showed reduction in the center towards the 3′ region of the gene body of LDL3 bound genes. H3K4me2 reduction in the same region of LDL3 unbound genes was not observed.

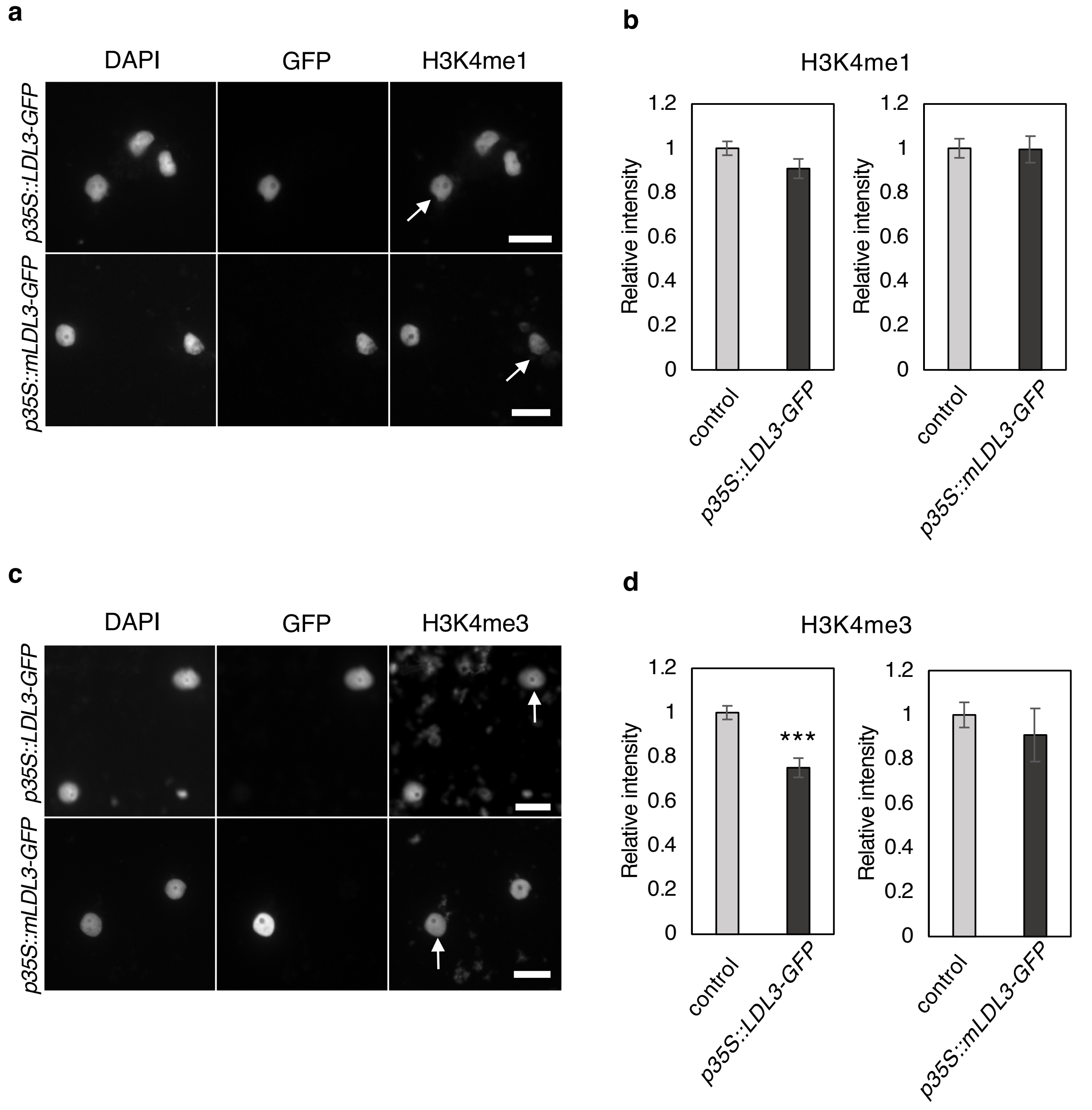

Supplementary Fig. 6. Demethylase activity of LDL3 for H3K4me1 and H3K4me3.
Nuclei transfected with *p35S::LDL3-GFP* or a mutated construct *p35S::mLDL3-GFP* were mixed with control nuclei without transfection. All nuclei were visualized by DAPI staining, and GFP and H3K4me1 (**a, b**) or H3K4me3 (**c, d**) were visualized by immunostaining. The transfected nuclei with GFP signal versus non-transfected nuclei without GFP signal (control) were observed. A maximum of five control nuclei per one transfected nucleus were randomly picked up from the same field and assessed for signal intensity. Arrows indicate nuclei transfected with *p35S::LDL3-GFP* or *p35S::mLDL3-GFP*. Scale bars: 20 µm. ***, $p < 0.001$ (Student's t test). $106 < n < 269$ (Control), $53 < n < 67$ (*p35S::LDL3-GFP*), $27 < n < 37$ (*p35S::mLDL3-GFP*). Source data are provided as a Source Data file.

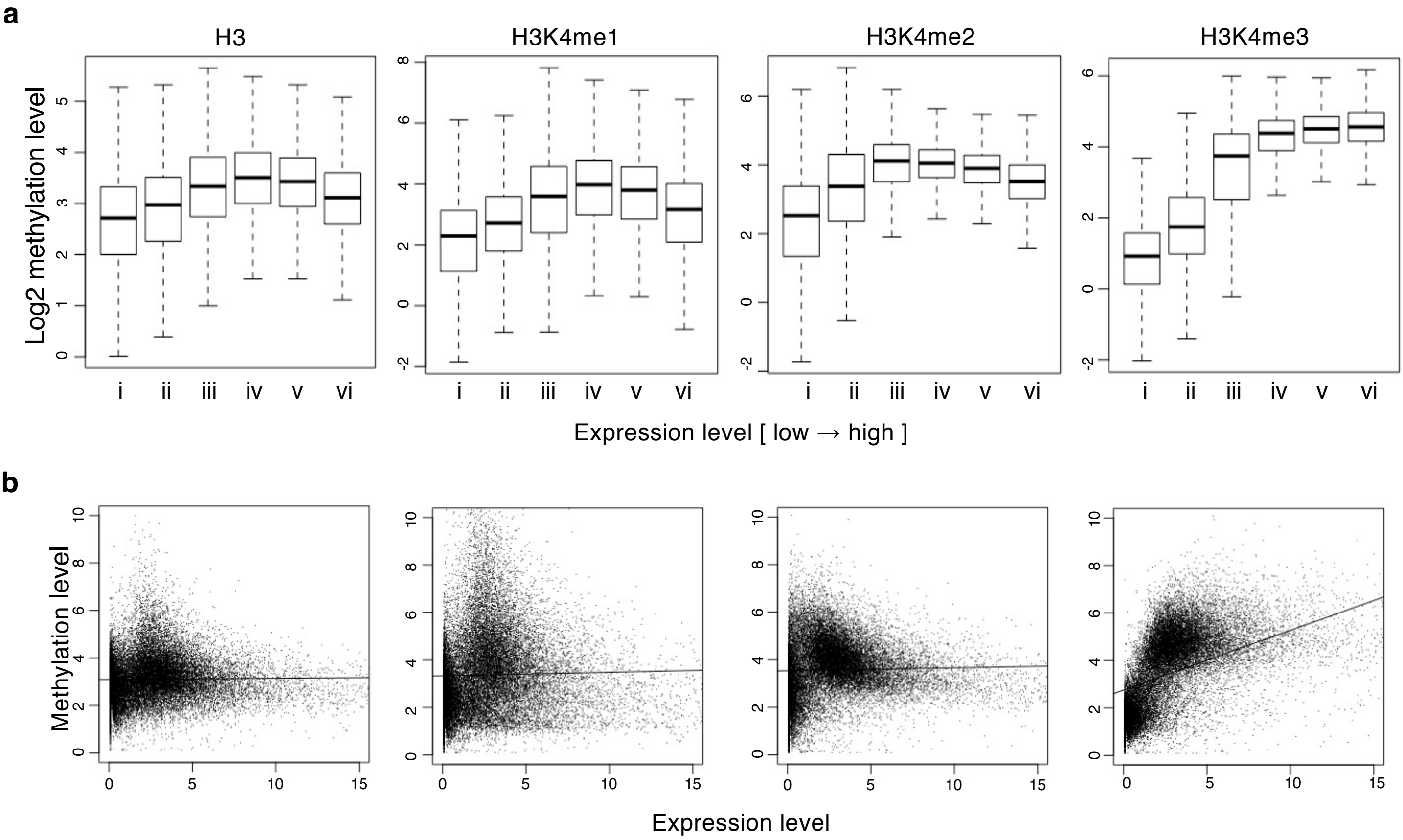

Supplementary Fig. 7. H3 and H3K4me levels with reference to each gene expression in WT callus.
a Based on the gene expression levels in WT at C14, genes were divided into six equal-sized groups from low signal to high (x-axis). The levels of H3 and H3K4 methylation **[**Log2 (read counts per kilobase million mapped reads: RPKM)**]** in WT at C14 were calculated and compared across groups. **b** Scatter plots of H3 and H3K4 methylation levels versus the gene expression levels in WT at C14. Each dot represents the square root of RPKM. Lines are linear regressions of the data. Only H3K4me3 showed a strong positive correlation with gene expression levels, compared with H3.

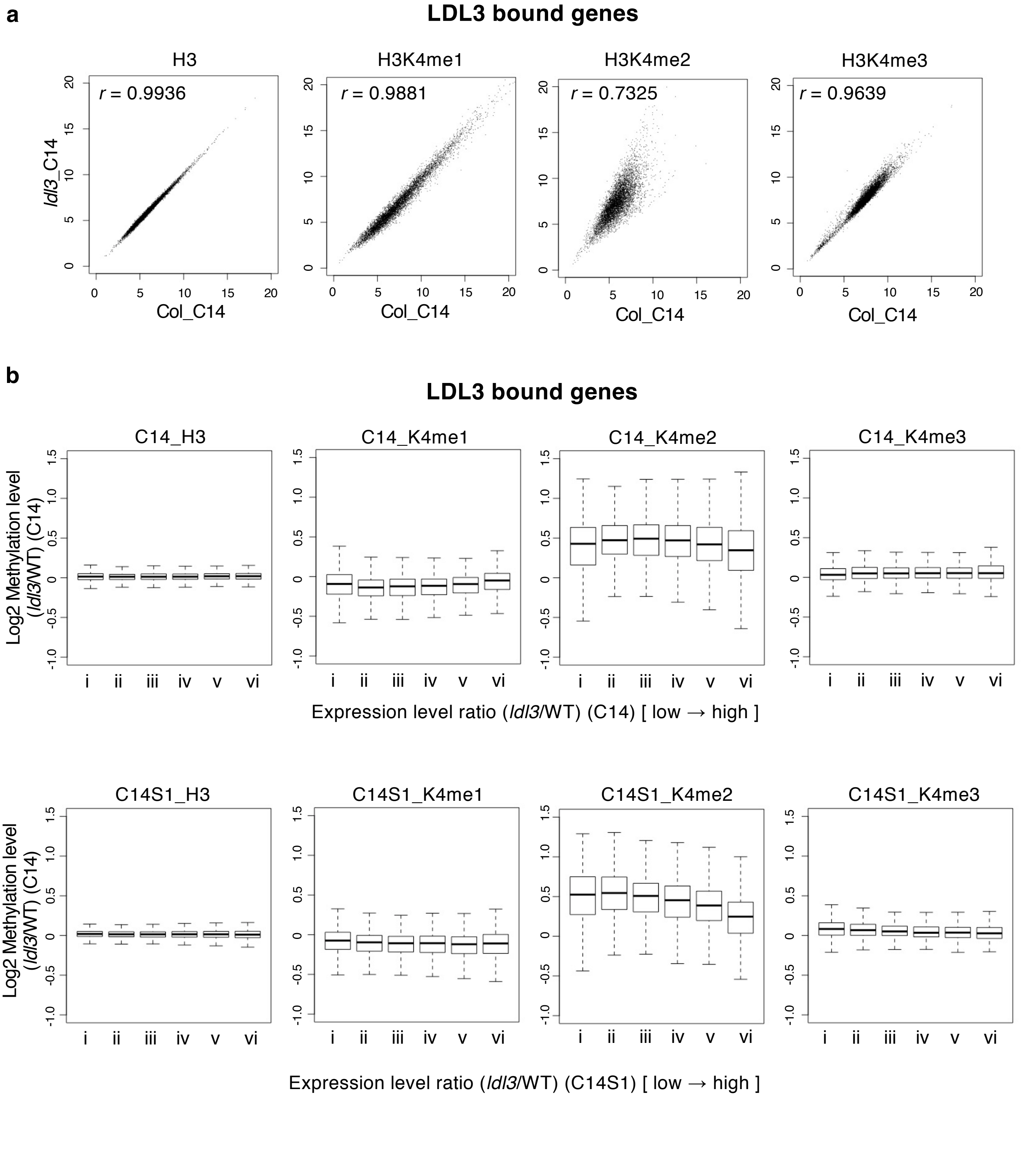

Supplementary Fig. 8. Changes in H3 and H3K4me levels of LDL3-bound genes in *ldl3* calli with reference to each altered gene expression before and after shoot induction.

a H3 and H3K4 methylation levels of LDL3-bound genes in *ldl3* compared with WT at C14 callus. Each dot represents the square root of RPM. Pearson correlation coefficient (*r*) is shown in each panel. Compared with wild-type callus, only H3K4me2 among the three H3K4methylations showed up-regulation at LDL3-bound genes in *ldl3* callus. **b** On the basis of the changes in gene expression levels (RPKM_*ldl3*/WT) at C14 (top) or C14S1(bottom), LDL3-bound genes were divided into six equal-sized groups from low signal to high (x-axis). The levels of H3 and H3K4 methylation [Log2 (RPKM_*ldl3*/WT)] at C14 were calculated and compared across groups. The hyper-H3K4me2, but neither hyper-H3K4me1 nor -H3K4me3, caused by *ldl3* in callus affects later gene activation, upon shoot induction, but not during callus formation.

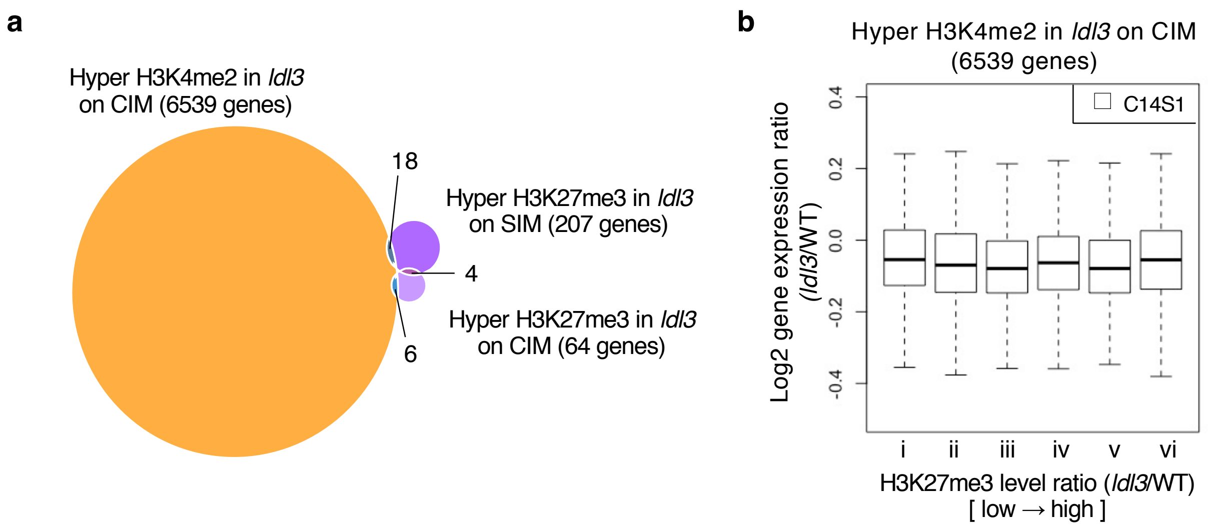

Supplementary Fig. 9. State of H3K27me3 and gene expression changes of hyper H3K4me2 in *ldl3* .
a Venn diagram of hyper H3K4me2 genes in *ldl3* at C14 (read count_*ldl3*/WT > 1.5, $p < 0.01$) and hyper H3K27me3 genes in *ldl3* at C14 and C14S1 (read count_*ldl3*/WT > 1.5, $p < 0.01$). **b** Altered gene expression of hyper H3K4me2 genes in *ldl3* with reference to hyper H3K27me3 caused by *ldl3* upon shoot induction. Based on H3K27me3 changes between WT and *ldl3* (RPKM_*ldl3*/WT) at C14S1, hyper H3K4me2 genes were divided into six equal-sized groups (i to vi) from low to high signal (x-axis). Gene expression fold-changes between WT and *ldl3* **[**Log2 (RPKM_*ldl3*/WT)**]** at C14S1 were calculated and compared across groups.

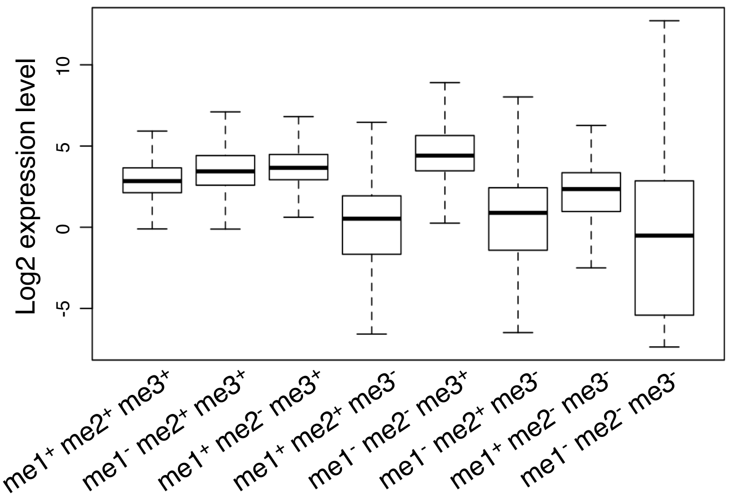

Supplementary Fig. 10. Gene expression with reference combination of H3K4me marks in WT callus. Genes with high level of H3K4me1, H3K4me2 and H3K4me3 (> 20 RPKM) in WT callus (C14) were counted as $me1^+$, $me2^+$, and $me3^+$, respectively ($me1^+$, 5867 genes; $me2^+$, 6032 genes; $me3^+$, 9254 genes). All genes were classified into eight groups according to the combinations of H3K4me marks ($me1^+me2^+me3^+$, 1814 genes; $me1^-me2^+me3^+$, 1220 genes; $me1^+me2^-me3^+$, 2195 genes; $me1^+me2^+me3^-$, 925 genes; $me1^-me2^-me3^+$, 4025 genes; $me1^-me2^+me3^-$, 2073 genes; $me1^+me2^-me3^-$, 933 genes; $me1^-me2^-me3^-$, 12992 genes), and the expression levels (Log2) of each group of genes were plotted.

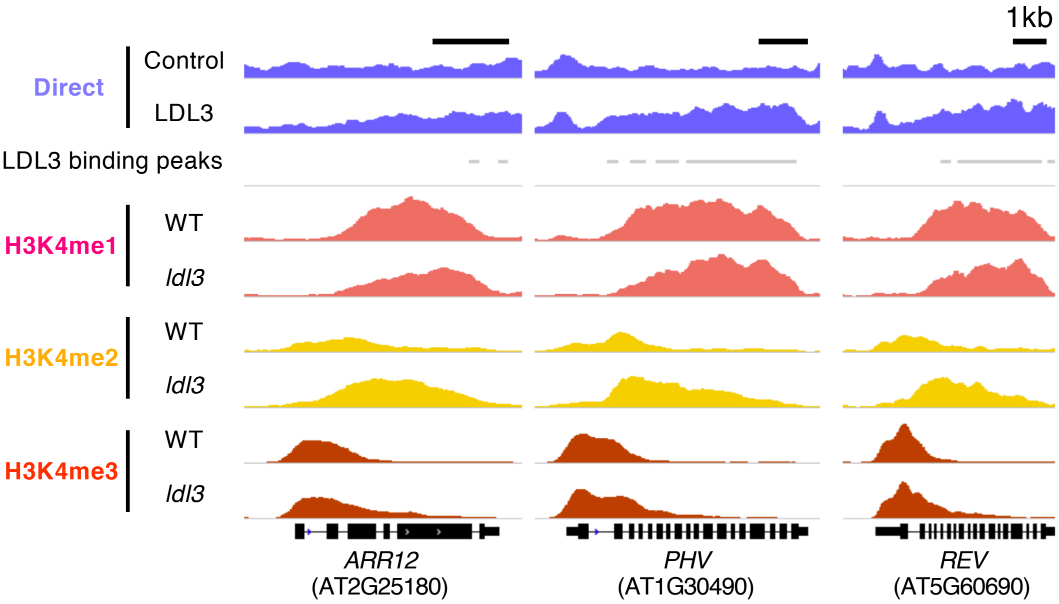

Supplementary Fig. 11. LDL3-bound genes with hyper H3K4me2.
Among the shoot meristem genes listed in Fig. 3b, those that are bound and H3K4me2-demethylated by LDL3 during callus formation were selected. H3K4 methylation and LDL3 binding patterns in selected genes are shown. Boxes, exons; thin lines, introns; bold lines at both ends, 5′ or 3′ UTR.
