# Supplementary material for: Primed histone demethylation regulates shoot regenerative competency
Source: Nat Commun. 2019 Apr 16;10:1786. doi: 10.1038/s41467-019-09386-5 (PMC6467990; doi:10.1038/s41467-019-09386-5)
Supplement: Supplementary file 3 — Description of Additional Supplementary Files [file 41467_2019_9386_MOESM3_ESM.pdf]

Description of Additional Supplementary Files

File Name: Supplementary Data 1
Description: RNA-seq and ChIP-seq experiment summary

File Name: Supplementary Data 2
Description: Pearson correlation between biological replicates in RNA-seq and ChIP-seq experiments

File Name: Supplementary Data 3
Description: Differentially expressed genes in WT upon shoot induction

File Name: Supplementary Data 4
Description: Gene expression of SAM and RAM genes (RPM)

File Name: Supplementary Data 5
Description: Gene lists for identifying the LDL3 target candidate genes

File Name: Supplementary Data 6
Description: Gene expression & H3K4me2 methylation levels (RPM) in LDL3 target candidate genes
