# Supplementary material for: Primed histone demethylation regulates shoot regenerative competency
Source: Nat Commun. 2019 Apr 16;10:1786. doi: 10.1038/s41467-019-09386-5 (PMC6467990; doi:10.1038/s41467-019-09386-5)
Supplement: Supplementary file 4 — Supplementary Data 1 [file 41467_2019_9386_MOESM4_ESM.pdf]

Supplementary Data 1. RNA-seq and ChIP-seq experiment summary

Experiment	Genotype	Stage	Replicate	Type of run	Raw reads	Mapped reads	Percentage mapped
**RNA-seq**	Col-0	C0	Rep1	36bp single-end	11638394	10535934	90.5%
			Rep2	64bp single-end	13637041	12963622	95.1%
			Rep3	84bp single-end	24714980	23295370	94.3%
	ldl3-1	C0	Rep1	76bp single-end	16973378	14173272	83.5%
			Rep2	64bp single-end	9529144	9109534	95.6%
	Col-0	C14	Rep1	64bp single-end	12607593	11845583	94.0%
			Rep2	84bp single-end	11416864	10618482	93.0%
			Rep3	84bp single-end	14730114	13722219	93.2%
	ldl3-1	C14	Rep1	64bp single-end	10015536	9533262	95.2%
			Rep2	84bp single-end	15125526	13754985	90.9%
			Rep3	84bp single-end	13937237	13034468	93.5%
	Col-0	C14S1	Rep1	36bp single-end	10478910	9666241	92.2%
			Rep2	64bp single-end	14739085	13845342	93.9%
			Rep3	84bp single-end	22890114	20710315	90.5%
	ldl3-1	C14S1	Rep1	76bp single-end	17548193	13208985	75.3%
			Rep2	64bp single-end	11285241	10715177	94.9%
			Rep3	84bp single-end	20683562	19195837	92.8%
	Col-0	C14S7	Rep1	74bp single-end	15734727	9173665	58.3%
			Rep2	74bp single-end	12104799	8231352	68.0%
			Rep3	86bp single-end	14027978	11735775	83.7%
	ldl3-1	C14S7	Rep1	74bp single-end	13246505	10458081	78.9%
			Rep2	74bp single-end	12058979	10608397	88.0%
			Rep3	86bp single-end	15150900	13719017	90.5%
**ChIP-seq H3**	Col-0	C0	Rep1	86bp single-end	27582188	19971327	72.4%
			Rep2	86bp single-end	24632918	17795578	72.2%
	ldl3-1	C0	Rep1	86bp single-end	25381761	17956773	70.7%
			Rep2	86bp single-end	21568044	15775622	73.1%
	Col-0	C14	Rep1	86bp single-end	29176133	21029319	72.1%
			Rep2	86bp single-end	19862114	14372865	72.4%
	ldl3-1	C14	Rep1	86bp single-end	29646595	21062636	71.0%
			Rep2	86bp single-end	21837868	14428393	66.1%
	Col-0	C14S1	Rep1	86bp single-end	24248405	15967281	65.8%
			Rep2	86bp single-end	20311423	11844896	58.3%
	ldl3-1	C14S1	Rep1	86bp single-end	21800097	13859021	63.6%
			Rep2	86bp single-end	25915660	20765879	80.1%
**ChIP-seq H3K4me1**	Col-0	C0	Rep1	86bp single-end	30223203	23538467	77.9%
			Rep2	86bp single-end	21155272	15083065	71.3%
	ldl3-1	C0	Rep1	86bp single-end	30660942	25401957	82.8%
			Rep2	86bp single-end	20613586	17261375	83.7%
	Col-0	C14	Rep1	86bp single-end	28501567	25834875	90.6%
			Rep2	86bp single-end	20870010	18220377	87.3%
	ldl3-1	C14	Rep1	86bp single-end	29533156	26708281	90.4%
			Rep2	86bp single-end	20396412	17879229	87.7%
**ChIP-seq H3K4me2**	Col-0	C0	Rep1	86bp single-end	29349376	19660085	67.0%
			Rep2	86bp single-end	21804470	17011705	78.0%
	ldl3-1	C0	Rep1	86bp single-end	33033918	25884230	78.4%
			Rep2	86bp single-end	22711677	19354335	85.2%
	Col-0	C14	Rep1	86bp single-end	31059573	28323323	91.2%
			Rep2	86bp single-end	23914076	20158383	84.3%
	ldl3-1	C14	Rep1	86bp single-end	32383031	29384752	90.7%
			Rep2	86bp single-end	24022021	21775375	90.6%
	Col-0	C14S1	Rep1	86bp single-end	23559491	19738729	83.8%
			Rep2	86bp single-end	18340661	9597203	52.3%
	ldl3-1	C14S1	Rep1	86bp single-end	30231414	24275453	80.3%
			Rep2	86bp single-end	23385916	18656144	79.8%
**ChIP-seq H3K4me3**	Col-0	C0	Rep1	86bp single-end	27939110	22423178	80.3%
			Rep2	86bp single-end	22430222	15837182	70.6%
	ldl3-1	C0	Rep1	86bp single-end	32074034	21321060	66.5%
			Rep2	86bp single-end	23071986	19319153	83.7%
	Col-0	C14	Rep1	86bp single-end	31659393	28778552	90.9%
			Rep2	86bp single-end	23197814	20384878	87.9%
	ldl3-1	C14	Rep1	86bp single-end	31151073	23752565	76.2%
			Rep2	86bp single-end	24377568	20708102	84.9%
**ChIP-seq H3K27me3**	Col-0	C14	Rep1	86bp single-end	14319809	8536281	59.6%
			Rep2	86bp single-end	16505985	11545922	70.0%
	ldl3-1	C14	Rep1	86bp single-end	15551443	9818956	63.1%
			Rep2	86bp single-end	25383781	19050397	75.1%
	Col-0	C14S1	Rep1	86bp single-end	13829185	7854425	56.8%
			Rep2	86bp single-end	12142698	7950504	65.5%
	ldl3-1	C14S1	Rep1	86bp single-end	11198558	7441592	66.5%
			Rep2	86bp single-end	22870798	14973106	65.5%
**Direct ChIP-seq**	*pLDL3::LDL3-GFP/ldl3-1*	C14	Rep1	86bp single-end	18862269	12969423	68.8%
	p35S:GFP	C14	Rep1	86bp single-end	19354833	12517210	64.7%
