# Supplementary material for: Primed histone demethylation regulates shoot regenerative competency
Source: Nat Commun. 2019 Apr 16;10:1786. doi: 10.1038/s41467-019-09386-5 (PMC6467990; doi:10.1038/s41467-019-09386-5)
Supplement: Supplementary file 6 — Supplementary Data 3 [file 41467_2019_9386_MOESM6_ESM.pdf]

Supplementary Data 3. Differentially expressed genes in WT upon shoot induction

	Col_C14_1	Col_C14_2	Col_C14_3	Col_C14S7_1	Col_C14S7_2	Col_C14S7_3	*p.value*	Log fold change	Gene annotation
**AT1G01110**	39.50839735	25.89824045	34.54251823	15.58809919	14.69989377	22.92136651	0.000732983	-0.834311009	IQ-domain 18
**AT1G01160**	51.495988	50.66637585	62.23483243	30.74016764	31.82952205	40.13369377	0.002547638	-0.619108614	GRF1-interacting factor 2
**AT1G01300**	171.7939927	203.3247314	236.6235373	100.7231025	104.843044	147.923763	0.001859086	-0.729139159	Eukaryotic aspartyl protease family protein
**AT1G01510**	102.4854581	83.34524652	107.3441548	55.37590483	57.09876093	64.92967018	0.000596971	-0.668874826	NAD(P)-binding Rossmann-fold superfamily protein
**AT1G01570**	9.28616177	8.569963202	12.02429432	3.488245974	4.252035389	4.175267505	6.75958E-06	-1.274180518	Protein of unknown function (DUF604)
**AT1G01580**	16.63067153	13.37290961	21.35223173	4.033284407	4.859469016	5.6238297	2.97792E-10	-1.757576182	ferric reduction oxidase 2
**AT1G01600**	7.091250806	11.3952258	7.797572681	1.526107613	1.214867254	2.897124391	8.81887E-08	-2.115625754	cytochrome P450, family 86, subfamily A, polypeptide 4
**AT1G01610**	49.30107703	78.07142302	46.20244	24.30871413	24.29734508	28.45998666	3.45429E-06	-1.124546889	glycerol-3-phosphate acyltransferase 4
**AT1G01630**	50.39853252	42.56728975	49.55466751	24.63573719	30.97911497	24.28471916	1.12573E-05	-0.794862282	Sec14p-like phosphatidylinositol transfer family protein
**AT1G01680**	16.54625188	22.03704823	5.028341262	1.30809224	2.551221233	2.897124391	2.24171E-07	-2.621762164	plant U-box 54
**AT1G01690**	18.23464493	15.82147053	20.62348662	11.11878404	11.66272564	8.180115928	0.000343408	-0.792393464	putative recombination initiation defects 3
**AT1G01930**	16.37741258	11.11269954	19.38461994	8.284584187	8.018123876	7.072391896	0.000167299	-0.96249138	zinc finger protein-related
**AT1G01940**	35.62509334	40.21290426	37.96762025	20.05741435	18.22300881	23.60304283	5.33318E-05	-0.821626942	Cyclophilin-like peptidyl-prolyl cis-trans isomerase family protein
**AT1G01950**	32.33272689	29.57108182	39.78948303	22.78260652	22.4750442	23.09178559	0.006343598	-0.52539349	armadillo repeat kinesin 2
**AT1G01960**	88.38737612	86.82973706	133.2874807	73.58018851	67.91107949	47.88776199	0.001641178	-0.675994394	SEC7-like guanine nucleotide exchange family protein
**AT1G02060**	18.65674319	13.27873419	21.20648271	8.502599561	9.354477855	7.583649141	2.36935E-05	-1.023450504	Tetratricopeptide repeat (TPR)-like superfamily protein
**AT1G02080**	114.2197898	121.0154144	222.9960038	88.73225696	82.48948654	45.16105668	0.000110768	-1.063856563	transcription regulators
**AT1G02110**	58.92491741	54.43339265	93.0607506	31.50322145	29.52127427	36.46968351	1.01812E-05	-1.025088629	Protein of unknown function (DUF630 and DUF632)
**AT1G02130**	70.65924911	64.2276363	66.97167565	48.39941288	46.52941582	37.57740754	0.000985797	-0.572321578	RAS 5
**AT1G02270**	15.6176357	11.86610289	17.63563167	10.79176098	10.20488493	7.839277764	0.009617446	-0.614369256	Calcium-binding endonuclease/exonuclease/phosphatase family
**AT1G02290**	24.48169921	22.41374991	22.00810233	15.47909151	17.12962828	13.97436471	0.00609526	-0.526034068	unknown protein
**AT1G02370**	33.93670029	30.60701144	27.47369066	20.27542972	20.04530969	21.81364247	0.007737725	-0.513752571	Tetratricopeptide repeat (TPR)-like superfamily protein
**AT1G02390**	9.877099337	9.32336656	11.14980019	3.2702306	3.766088487	3.493591177	4.8697E-08	-1.478631602	glycerol-3-phosphate acyltransferase 2
**AT1G02410**	35.54067368	30.04195892	32.3562829	21.80153734	24.66180525	17.89400359	0.001875311	-0.572311645	cytochrome c oxidase assembly protein CtaG / Cox11 family
**AT1G02520**	43.89821928	44.82749983	69.37653451	16.56916837	13.84948669	17.55316543	6.77839E-13	-1.664960758	P-glycoprotein 11
**AT1G02530**	18.91000215	14.78554091	23.53846707	3.2702306	2.065274332	3.067543473	5.53988E-20	-2.698241932	P-glycoprotein 12
**AT1G02670**	4.052143318	2.448560915	3.352227508	1.199084553	1.214867254	1.278143114	0.000334758	-1.353523712	P-loop containing nucleoside triphosphate hydrolases superfamily protein
**AT1G02730**	53.85973827	68.18300394	68.57491489	38.04368265	44.46414149	44.22375173	0.00581174	-0.542725566	cellulose synthase-like D5
**AT1G02800**	0.759776872	0.565052519	0.801619621	0.218015373	0.121486725	0	0.001599964	-2.609512157	cellulase 2
**AT1G02810**	43.64496032	42.66146517	56.7692441	14.49802233	18.58746898	19.76861349	1.33991E-10	-1.380116859	Plant invertase/pectin methylesterase inhibitor superfamily
**AT1G02850**	218.5624802	291.0962226	274.1539105	155.5539689	137.6444599	116.1406043	3.09799E-06	-0.907408506	beta glucosidase 11
**AT1G02970**	16.54625188	16.76322472	16.25101596	7.194507321	8.261097326	6.135086946	2.603E-07	-1.165639148	WEE1 kinase homolog
**AT1G02990**	16.20857327	11.3952258	25.7247024	8.938630308	11.41975219	4.345686587	0.00133367	-1.100526459	unknown protein
**AT1G03100**	10.21477795	8.287436942	11.44129823	5.123361274	4.009061938	6.135086946	0.00179097	-0.901696929	Pentatricopeptide repeat (PPR) superfamily protein
**AT1G03230**	190.8728342	215.5675359	245.7328512	106.0644791	112.8611679	123.979882	9.90577E-06	-0.877218506	Eukaryotic aspartyl protease family protein
**AT1G03270**	13.25388544	11.58357664	14.86640025	5.014353587	4.373522114	6.220296487	1.92036E-06	-1.277494783	CBS domain-containing protein with a domain of unknown function (DUF21)
**AT1G03300**	9.539420728	5.933051447	13.40891003	2.507176794	2.915681409	3.152753014	1.67622E-06	-1.689663772	DOMAIN OF UNKNOWN FUNCTION 724 1
**AT1G03365**	23.72192234	19.21178564	29.95142404	13.95298389	13.97097342	12.01454527	0.000135977	-0.828635839	RING/U-box superfamily protein
**AT1G03457**	27.3519674	21.66034655	35.48988688	11.55481479	12.99907962	6.987182355	1.75071E-08	-1.402870582	RNA-binding (RRM/RBD/RNP motifs) family protein
**AT1G03530**	34.2743789	27.68757342	52.46964795	11.11878404	13.36353979	16.61586048	1.25547E-07	-1.410726539	nuclear assembly factor 1
**AT1G03540**	2.194910964	2.071859236	2.987854953	0.545038433	0.971893803	1.363352655	0.005681845	-1.236795396	Pentatricopeptide repeat (PPR-like) superfamily protein
**AT1G03687**	28.95594079	32.01964273	34.83401628	23.54566032	20.89571677	19.68340395	0.00370653	-0.540572304	DTW domain-containing protein
**AT1G03760**	38.74862048	35.31578243	47.58705571	21.36550659	25.39072561	25.30723365	0.000366896	-0.704418427	Prefoldin chaperone subunit family protein
**AT1G03770**	33.59902168	17.23410182	32.64778095	10.57374561	11.78421236	11.58849756	1.30806E-06	-1.242316797	RING 1B
**AT1G03780**	18.15022528	12.14862915	20.69636114	4.360307467	6.560283171	3.237962555	7.17059E-10	-1.829695279	targeting protein for XKLP2
**AT1G03800**	1.772812702	1.789332976	2.842105931	0.32702306	0.728920352	0.766885868	0.000361566	-1.731390563	ERF domain protein 10
**AT1G03830**	35.87835229	23.54385495	44.38057722	13.0809224	14.8213805	19.76861349	6.16061E-05	-1.051830096	guanylate-binding family protein
**AT1G03860**	141.6561768	122.0513441	134.8178454	95.92676428	95.48856616	88.70313209	0.007447653	-0.465172923	prohibitin 2
**AT1G03910**	90.16018882	64.03928546	104.5020488	41.85895168	48.23022998	50.27362914	0.000113029	-0.826115563	unknown protein
**AT1G04010**	17.98138597	16.19817221	20.40486309	13.0809224	12.14867254	7.328020518	0.002215952	-0.727045741	phospholipid sterol acyl transferase 1
**AT1G04020**	25.24147608	24.48560915	31.19029072	10.68275329	12.87759289	10.05472583	3.7197E-09	-1.230784354	breast cancer associated RING 1
**AT1G04050**	21.78027033	17.32827724	24.4129612	9.156645681	9.840424756	9.543468582	7.03861E-07	-1.105275099	homolog of SU(var)3-9 1
**AT1G04080**	181.8399314	173.0944216	225.4737372	131.3542624	123.3090263	97.13887664	0.000213837	-0.688854161	Tetratricopeptide repeat (TPR)-like superfamily protein
**AT1G04100**	7.006831154	6.498103966	8.38056877	4.9053459	4.495008839	3.067543473	0.005873708	-0.790139751	indoleacetic acid-induced protein 10
**AT1G04150**	3.967723665	4.70877099	7.141702082	0.218015373	0.364460176	0.426047705	4.83642E-15	-3.85462236	C2 calcium/lipid-binding plant phosphoribosyltransferase family protein
**AT1G04160**	10.89013517	9.229191141	17.05263558	4.796338214	4.373522114	3.067543473	3.4324E-07	-1.577180117	myosin XI B
**AT1G04180**	16.54625188	11.3952258	6.048584416	4.25129978	4.130548663	6.135086946	0.002380958	-1.149726941	YUCCA 9
**AT1G04200**	42.46308519	36.35171204	58.29960883	31.28520608	25.14775216	17.46795589	0.000108842	-0.867127339	unknown protein
**AT1G04210**	85.60152759	96.5298053	116.8178412	75.43331918	74.47136266	46.18357117	0.00457626	-0.586702641	Leucine-rich repeat protein kinase family protein
**AT1G04220**	26.84544948	34.65655449	31.91903584	18.42229905	21.26017694	16.10460323	0.00033527	-0.712207583	3-ketoacyl-CoA synthase 2
**AT1G04300**	47.69710364	38.89444838	80.5263347	38.58872108	36.56750434	26.75579585	0.005599927	-0.681258147	TRAF-like superfamily protein
**AT1G04510**	108.4792534	100.7676992	128.4048885	54.61285103	55.0334866	43.03081816	1.48077E-09	-1.110353131	MOS4-associated complex 3A
**AT1G04520**	56.56116715	42.56728975	49.19029495	29.75909846	28.54938047	22.15448064	3.16318E-06	-0.847186706	plasmodesmata-located protein 2
**AT1G04600**	3.039107488	1.883508396	7.287451104	0.545038433	0.485946902	0.511257245	6.27675E-07	-2.909856914	myosin XI A
**AT1G04610**	102.9075563	53.11493677	124.7611629	16.46016069	16.40070793	27.18184355	9.7161E-12	-2.142410712	YUCCA 3
**AT1G04650**	9.708260032	9.040840301	11.95141981	3.379238287	2.794194684	2.130238523	1.75245E-10	-1.857913631	unknown protein
**AT1G04660**	0.506517915	0.847578778	0.364372555	0.109007687	0	0	0.000501719	-3.603132368	glycine-rich protein
**AT1G04700**	21.02049346	13.93796213	32.72065546	5.450384334	6.195822995	4.516105668	4.76378E-11	-2.029014993	PB1 domain-containing protein tyrosine kinase
**AT1G04730**	19.83861833	21.18946946	28.27531028	8.938630308	7.653663699	5.368201077	2.97513E-11	-1.635280621	P-loop containing nucleoside triphosphate hydrolases superfamily protein
**AT1G04770**	49.97643425	51.98483173	51.23078126	33.24734444	31.34357515	35.87321672	0.002330592	-0.557334666	Tetratricopeptide repeat (TPR)-like superfamily protein
**AT1G04790**	36.72254882	26.55746838	42.34009091	25.18077562	26.48410613	18.40526084	0.006834123	-0.560681177	RING/U-box superfamily protein
**AT1G04810**	89.73809056	85.69963202	101.4413194	60.39025842	57.09876093	53.59680123	0.000233881	-0.652976496	26S proteasome regulatory complex, non-ATPase subcomplex, Rpn2/Psmd1 subunit
**AT1G04850**	163.7741258	121.9571686	166.3725087	79.03057284	90.14315024	111.9653368	0.004414098	-0.621307585	ubiquitin-associated (UBA)/TS-N domain-containing protein
**AT1G04900**	39.93049561	30.98371311	43.43320858	26.70688324	29.39978754	17.80879405	0.002963878	-0.604183942	Protein of unknown function (DUF185)
**AT1G04945**	23.04656512	22.03704823	25.65182789	12.31786859	13.72799997	8.094906387	2.35105E-06	-1.030750677	HIT-type Zinc finger family protein
**AT1G04990**	43.89821928	42.56728975	57.35224019	37.71665959	31.46506188	29.5677107	0.007509793	-0.504317112	Zinc finger C-x8-C-x5-C-x3-H type family protein
**AT1G05010**	286.0137825	303.2448518	313.433272	101.2681409	99.01168119	194.2777533	7.61067E-05	-1.115387007	ethylene-forming enzyme
**AT1G05120**	58.33397985	41.34300929	66.75305211	26.05283712	32.19398223	22.75094742	1.2043E-06	-1.002067964	Helicase protein with RING/U-box domain
**AT1G05135**	3.967723665	4.991297249	8.890690347	0.109007687	0.121486725	0.255628623	2.6516E-17	-4.986496109	pseudogene
**AT1G05280**	4.980759495	5.650525188	6.558705994	1.96213836	1.822300881	1.533771736	4.10962E-07	-1.654674955	Protein of unknown function (DUF604)
**AT1G05380**	50.56737182	30.79536227	70.68827571	27.90596779	24.29734508	19.25735625	3.62855E-05	-1.052273212	Acyl-CoA N-acyltransferase with RING/FYVE/PHD-type zinc finger protein
**AT1G05420**	1.013035829	1.130105038	2.331984353	0.436030747	0.485946902	0.596466786	0.007242351	-1.485421386	ovate family protein 12
**AT1G05440**	5.993795324	7.063156485	4.809717729	3.2702306	3.766088487	1.959819441	0.003137268	-0.974749963	C-8 sterol isomerases
**AT1G05460**	21.52701138	17.1399264	29.29555344	9.047637994	7.167716798	9.032211337	8.08748E-08	-1.371557868	P-loop containing nucleoside triphosphate hydrolases superfamily protein
**AT1G05490**	2.026071659	1.977683816	1.894737287	0.65404612	0.364460176	0.340838164	1.45506E-05	-2.058022425	chromatin remodeling 31
**AT1G05577**	31.74178932	25.70988961	30.31579659	5.668399707	3.401628311	5.964667864	2.34303E-22	-2.469883313	Domain of unknown function (DUF966)
**AT1G05630**	17.30602875	10.4534716	21.42510625	8.938630308	11.05529201	7.668858682	0.004043039	-0.795382889	Endonuclease/exonuclease/phosphatase family protein
**AT1G05640**	12.49410856	9.982594499	12.60729041	7.739545754	6.681769896	6.987182355	0.005972567	-0.664657355	Ankyrin repeat family protein
**AT1G05680**	2.363750269	2.542736335	3.206478486	0.872061493	1.093380528	0.596466786	6.79758E-05	-1.647750315	Uridine diphosphate glycosyltransferase 74E2
**AT1G05810**	13.08504613	18.5525577	11.07692568	8.720614934	8.504070777	5.964667864	0.001864289	-0.856846808	RAB GTPase homolog A5E
**AT1G05910**	53.09996139	49.15956914	79.36034252	16.89619143	18.22300881	17.97921313	3.52775E-15	-1.725050514	cell division cycle protein 48-related / CDC48-related
**AT1G05950**	16.79951084	11.11269954	18.94737287	7.739545754	6.803256622	4.771734291	4.14075E-06	-1.251155475	unknown protein
**AT1G06070**	36.13161125	32.49051983	46.93118511	19.83939898	21.38166367	13.71873609	5.47042E-07	-1.047087108	Basic-leucine zipper (bZIP) transcription factor family protein
**AT1G06080**	362.5824073	512.8793362	270.655934	90.14935688	84.31178742	122.1052721	5.91934E-12	-1.89603043	delta 9 desaturase 1
**AT1G06210**	56.89884576	50.38384959	54.65588328	29.54108309	37.41791142	32.46483509	0.000270199	-0.658486237	ENTH/VHS/GAT family protein
**AT1G06220**	118.4407724	113.7639071	146.4048927	62.57041215	61.47228305	68.76409952	2.08643E-06	-0.921838181	Ribosomal protein S5/Elongation factor G/III/V family protein
**AT1G06230**	50.90505043	51.13725295	77.31985621	32.37528294	29.88573445	21.21717569	4.80072E-07	-1.07622442	global transcription factor group E4
**AT1G06310**	12.15642995	7.063156485	7.943321703	5.014353587	3.887575212	3.408381636	0.000283321	-1.100697808	acyl-CoA oxidase 6
**AT1G06420**	28.53384253	17.32827724	27.25506713	3.161222914	4.616495565	5.709039241	1.12431E-15	-2.359290793	unknown protein
**AT1G06560**	32.41714654	29.28855556	40.37247912	22.23756808	23.68991145	21.55801385	0.00392344	-0.554032161	NOL1/NOP2/sun family protein
**AT1G06590**	32.6704055	30.70118686	37.53037319	21.91054502	20.77423004	15.84897461	5.9206E-05	-0.755023649	unknown protein
**AT1G06660**	17.55928771	17.42245266	18.43725129	8.066568814	10.20488493	10.22514491	0.000184768	-0.853171486	unknown protein
**AT1G06670**	84.25081315	62.24995249	123.7409197	33.02932906	34.62371674	28.54519621	5.41741E-10	-1.448315767	nuclear DEIH-boxhelicase
**AT1G06710**	13.25388544	11.77192748	17.92712972	9.592676428	7.775150425	3.323172096	0.001166764	-1.050519231	Tetratricopeptide repeat (TPR)-like superfamily protei
