# Supplementary material for: Primed histone demethylation regulates shoot regenerative competency
Source: Nat Commun. 2019 Apr 16;10:1786. doi: 10.1038/s41467-019-09386-5 (PMC6467990; doi:10.1038/s41467-019-09386-5)
Supplement: Supplementary file 7 — Supplementary Data 4 [file 41467_2019_9386_MOESM7_ESM.pdf]

Supplemetary Data 4. Gene expression of SAM and RAM genes (RPM)

Gene Name	Gene ID	Col_C14	ldl3_C14	Col_C14S1	ldl3_C14S1	Col_C14S7	ldl3_C14S7
WUS	AT2G17950	0	0.024233638	0.277801626	0.124434093	2.027416194	0.710571067
FIL	AT2G45190	0	0	0	0.062217047	1.926223801	0
DRNL	AT1G24590	0	0	0	0	0.186225801	0
CUC1	AT3G15170	0.589417825	0.536885324	2.501717439	1.592599933	5.401487299	1.295645812
CUC3	AT1G76420	0.122315304	0.102292885	0.964593536	0.55393885	0.913717	0.269900782
DRN	AT1G12980	0.262418267	0.250373881	0.510978476	0.341429807	1.044472506	0.835568375
ANT	AT4G37750	8.63150861	9.33889495	15.2490098	17.07585639	33.13110668	28.22002474
NANA	AT3G12700	6.089098437	9.776820827	12.85322541	12.19203957	19.31337705	19.45657363
MYB37	AT5G23000	0.457608082	0.481720133	1.272523685	1.370981612	1.39517629	1.231471317
CLE41	AT3G24770	8.837493468	11.47224896	16.35988768	20.49734709	23.41006573	27.3477681
CLE42	AT2G34925	0.848322842	0.619054475	1.080749357	1.219433589	2.07302087	1.92678858
OBE2	AT5G48160	134.4149096	116.6114887	194.6826456	131.1217039	215.8659211	173.7923111
RR12	AT2G25180	32.95780579	30.69514517	47.87799122	37.81468236	49.44103446	34.48764864
STM	AT1G62360	1.927933472	2.27132749	0.882653699	1.530632078	2.735268927	0.900218192
CLV3	AT2G27250	0.248814119	0.095503815	0.384646506	0.174276776	0.332401382	0
REV	AT5G60690	97.52572379	93.7246301	104.7949571	96.19945079	130.0346357	121.8026828
AS1	AT2G37630	20.71126068	15.23508807	22.48963571	14.89968532	23.94628168	17.69590402
TFL1	AT5G03840	0.542624192	0.644551536	0.611868815	0.505838814	0.473971929	0.143762554
CLV1	AT1G75820	8.604089482	5.641252375	5.886233693	4.185770921	7.327828836	5.020184692
PHV	AT1G30490	177.4294171	170.0120493	176.5833339	149.5996124	130.9310257	110.0602577
UFO	AT1G30950	0.121457518	0.382182575	0.024075486	0	0.085209541	0
KN	AT1G08560	107.6930524	93.45493999	81.2254594	94.69116503	72.93718192	66.29613319
CUC2	AT5G53950	25.71888692	25.53701263	37.3977405	40.95131533	16.69352691	9.293054749
ZLL	AT5G43810	165.6838059	178.1496848	147.39721	140.5326634	75.7917527	70.9343713
