# Supplementary material for: Primed histone demethylation regulates shoot regenerative competency
Source: Nat Commun. 2019 Apr 16;10:1786. doi: 10.1038/s41467-019-09386-5 (PMC6467990; doi:10.1038/s41467-019-09386-5)
Supplement: Supplementary file 8 — Supplementary Data 5 [file 41467_2019_9386_MOESM8_ESM.pdf]

Table S5. Gene lists for identifying the LDL3 target candidate genes

Hyper H3K4me2 in *ldl3* on CIM(6539 genes)	LDL3 bound genes on CIM(6243 genes)	Up-regulated by shoot induction in WT(UGs_S1, 1678 genes)	Down-regulated in *ldl3* compared to WT on SIM(932 genes)	LDL3 target candidate genes(16 genes)
AT1G01040	AT1G01040	AT1G01010	AT1G01060	AT1G18460
AT1G01046	AT1G01050	AT1G01090	AT1G01560	AT1G30270
AT1G01050	AT1G01090	AT1G01480	AT1G01680	AT1G76550
AT1G01160	AT1G01100	AT1G01490	AT1G02120	AT2G30600
AT1G01220	AT1G01160	AT1G01700	AT1G02250	AT2G34750
AT1G01230	AT1G01170	AT1G02205	AT1G02450	AT3G02470
AT1G01320	AT1G01220	AT1G02230	AT1G02840	AT3G22200
AT1G01440	AT1G01230	AT1G02250	AT1G03850	AT3G24180
AT1G01448	AT1G01320	AT1G02450	AT1G03870	AT3G55610
AT1G01450	AT1G01490	AT1G02500	AT1G04180	AT4G38470
AT1G01510	AT1G01510	AT1G02950	AT1G04280	AT4G39850
AT1G01540	AT1G01550	AT1G03440	AT1G04660	AT5G02880
AT1G01550	AT1G01650	AT1G03840	AT1G04710	AT5G13710
AT1G01650	AT1G01670	AT1G03850	AT1G04810	AT5G23380
AT1G01660	AT1G01690	AT1G03870	AT1G05240	AT5G53460
AT1G01670	AT1G01710	AT1G03920	AT1G05250	AT5G56890
AT1G01740	AT1G01820	AT1G04280	AT1G05400	
AT1G01750	AT1G01830	AT1G04680	AT1G05760	
AT1G01780	AT1G01840	AT1G04980	AT1G05880	
AT1G01820	AT1G01920	AT1G05340	AT1G06080	
AT1G01860	AT1G01940	AT1G05470	AT1G06430	
AT1G01910	AT1G01950	AT1G05570	AT1G06810	
AT1G01920	AT1G01960	AT1G05750	AT1G07000	
AT1G01950	AT1G02010	AT1G05760	AT1G08050	
AT1G01960	AT1G02080	AT1G05770	AT1G08210	
AT1G02010	AT1G02100	AT1G05805	AT1G08320	
AT1G02050	AT1G02110	AT1G05880	AT1G08500	
AT1G02080	AT1G02180	AT1G06000	AT1G08520	
AT1G02100	AT1G02270	AT1G06030	AT1G08590	
AT1G02120	AT1G02280	AT1G06040	AT1G08670	
AT1G02145	AT1G02305	AT1G06149	AT1G09010	
AT1G02270	AT1G02310	AT1G06180	AT1G09100	
AT1G02290	AT1G02320	AT1G06290	AT1G09430	
AT1G02305	AT1G02330	AT1G06490	AT1G09665	
AT1G02410	AT1G02370	AT1G06520	AT1G09750	
AT1G02430	AT1G02400	AT1G06980	AT1G09932	
AT1G02500	AT1G02410	AT1G06990	AT1G10340	
AT1G02520	AT1G02500	AT1G07200	AT1G10640	
AT1G02560	AT1G02560	AT1G07530	AT1G10960	
AT1G02680	AT1G02690	AT1G07880	AT1G11530	
AT1G02780	AT1G02740	AT1G08160	AT1G11540	
AT1G02840	AT1G02780	AT1G08320	AT1G12010	
AT1G02890	AT1G02840	AT1G08590	AT1G12040	
AT1G02910	AT1G02850	AT1G08900	AT1G12080	
AT1G02960	AT1G02870	AT1G08920	AT1G12090	
AT1G02990	AT1G02890	AT1G09210	AT1G12750	
AT1G03000	AT1G02990	AT1G09390	AT1G13260	
AT1G03040	AT1G03000	AT1G09430	AT1G13300	
AT1G03060	AT1G03040	AT1G09880	AT1G13710	
AT1G03080	AT1G03050	AT1G10070	AT1G13750	
AT1G03110	AT1G03060	AT1G10090	AT1G14160	
AT1G03150	AT1G03080	AT1G10120	AT1G14220	
AT1G03190	AT1G03090	AT1G10140	AT1G14540	
AT1G03250	AT1G03110	AT1G10340	AT1G14700	
AT1G03280	AT1G03140	AT1G10470	AT1G14960	
AT1G03290	AT1G03150	AT1G10585	AT1G15290	
AT1G03310	AT1G03190	AT1G10640	AT1G15740	
AT1G03320	AT1G03260	AT1G10700	AT1G16090	
AT1G03365	AT1G03270	AT1G10850	AT1G16390	
AT1G03495	AT1G03280	AT1G11260	AT1G17300	
AT1G03530	AT1G03310	AT1G11440	AT1G17600	
AT1G03545	AT1G03320	AT1G11500	AT1G18140	
AT1G03550	AT1G03330	AT1G11570	AT1G18270	
AT1G03560	AT1G03365	AT1G11610	AT1G18450	
AT1G03687	AT1G03370	AT1G11670	AT1G18460	
AT1G03760	AT1G03380	AT1G11915	AT1G19250	
AT1G03830	AT1G03390	AT1G12010	AT1G19530	
AT1G03860	AT1G03530	AT1G12080	AT1G19660	
AT1G03910	AT1G03540	AT1G12090	AT1G19960	
AT1G04010	AT1G03560	AT1G12240	AT1G20010	
AT1G04050	AT1G03590	AT1G12420	AT1G20190	
AT1G04080	AT1G03650	AT1G13110	AT1G20630	
AT1G04120	AT1G03670	AT1G13260	AT1G20960	
AT1G04140	AT1G03687	AT1G13300	AT1G21010	
AT1G04160	AT1G03750	AT1G13400	AT1G21110	
AT1G04170	AT1G03760	AT1G13420	AT1G21120	
AT1G04190	AT1G03770	AT1G13480	AT1G21525	
AT1G04200	AT1G03830	AT1G13500	AT1G21580	
AT1G04210	AT1G03860	AT1G13510	AT1G21680	
AT1G04230	AT1G03900	AT1G13740	AT1G21720	
AT1G04270	AT1G03910	AT1G13920	AT1G22430	
AT1G04300	AT1G03930	AT1G14120	AT1G22500	
AT1G04310	AT1G03960	AT1G14220	AT1G22640	
AT1G04340	AT1G03980	AT1G14240	AT1G22710	
AT1G04390	AT1G04010	AT1G14260	AT1G22770	
AT1G04400	AT1G04080	AT1G14290	AT1G22910	
AT1G04410	AT1G04120	AT1G14730	AT1G22980	
AT1G04430	AT1G04140	AT1G14860	AT1G23390	
AT1G04440	AT1G04160	AT1G14960	AT1G23720	
AT1G04510	AT1G04170	AT1G15080	AT1G23760	
AT1G04590	AT1G04190	AT1G15100	AT1G23840	
AT1G04620	AT1G04200	AT1G15330	AT1G23870	
AT1G04640	AT1G04210	AT1G15380	AT1G24792	
AT1G04690	AT1G04270	AT1G16022	AT1G24879	
AT1G04750	AT1G04300	AT1G16060	AT1G25141	
AT1G04780	AT1G04340	AT1G16390	AT1G25209	
AT1G04790	AT1G04390	AT1G16530	AT1G25230	
AT1G04800	AT1G04400	AT1G17190	AT1G25450	
AT1G04810	AT1G04410	AT1G17230	AT1G25560	
AT1G04820	AT1G04430	AT1G17300	AT1G26200	
AT1G04850	AT1G04440	AT1G18140	AT1G26270	
AT1G04860	AT1G04480	AT1G18270	AT1G26390	
AT1G04870	AT1G04510	AT1G18330	AT1G26410	
AT1G04940	AT1G04590	AT1G18460	AT1G26945	
AT1G04945	AT1G04610	AT1G18590	AT1G26960	
AT1G04950	AT1G04690	AT1G18620	AT1G27020	
AT1G04960	AT1G04750	AT1G18870	AT1G27410	
AT1G04970	AT1G04780	AT1G19050	AT1G27420	
AT1G05030	AT1G04790	AT1G19420	AT1G27595	
AT1G05090	AT1G04800	AT1G19610	AT1G29100	
AT1G05120	AT1G04810	AT1G19620	AT1G29520	
AT1G05160	AT1G04820	AT1G19660	AT1G30190	
AT1G05180	AT1G04830	AT1G19700	AT1G30270	
AT1G05270	AT1G04850	AT1G19960	AT1G30400	
AT1G05350	AT1G04860	AT1G20010	AT1G30410	
AT1G05360	AT1G04870	AT1G20190	AT1G30420	
AT1G05380	AT1G04900	AT1G20620	AT1G30750	
AT1G05410	AT1G04910	AT1G20840	AT1G30870	
AT1G05460	AT1G04950	AT1G20925	AT1G31770	

AT1G05500	AT1G04960	AT1G21100	AT1G31950
AT1G05620	AT1G04970	AT1G21110	AT1G32450
AT1G05785	AT1G04980	AT1G21120	AT1G32960
AT1G05830	AT1G05030	AT1G21130	AT1G32970
AT1G05835	AT1G05055	AT1G21400	AT1G33080
AT1G05840	AT1G05070	AT1G21520	AT1G33960
AT1G05850	AT1G05120	AT1G21525	AT1G36160
AT1G05890	AT1G05160	AT1G21670	AT1G36180
AT1G05900	AT1G05180	AT1G21810	AT1G43020
AT1G05910	AT1G05270	AT1G21920	AT1G43910
AT1G05960	AT1G05350	AT1G22330	AT1G44180
AT1G06050	AT1G05380	AT1G22400	AT1G45474
AT1G06060	AT1G05410	AT1G22430	AT1G50200
AT1G06070	AT1G05470	AT1G22440	AT1G51460
AT1G06130	AT1G05500	AT1G22530	AT1G51830
AT1G06149	AT1G05520	AT1G22640	AT1G52070
AT1G06150	AT1G05630	AT1G22650	AT1G52130
AT1G06190	AT1G05700	AT1G22710	AT1G52370
AT1G06200	AT1G05720	AT1G22880	AT1G52710
AT1G06210	AT1G05780	AT1G23160	AT1G53350
AT1G06220	AT1G05785	AT1G23205	AT1G53480
AT1G06250	AT1G05790	AT1G23210	AT1G53490
AT1G06260	AT1G05830	AT1G23390	AT1G54970
AT1G06265	AT1G05840	AT1G23530	AT1G55020
AT1G06390	AT1G05850	AT1G23870	AT1G55850
AT1G06410	AT1G05890	AT1G24100	AT1G55860
AT1G06430	AT1G05900	AT1G24120	AT1G56710
AT1G06460	AT1G05910	AT1G24147	AT1G57630
AT1G06470	AT1G05925	AT1G24807	AT1G59590
AT1G06550	AT1G05930	AT1G24822	AT1G60060
AT1G06560	AT1G05940	AT1G24909	AT1G60940
AT1G06590	AT1G05960	AT1G24996	AT1G61590
AT1G06670	AT1G06060	AT1G25083	AT1G62190
AT1G06700	AT1G06070	AT1G25097	AT1G62480
AT1G06720	AT1G06100	AT1G25155	AT1G62530
AT1G06730	AT1G06130	AT1G25220	AT1G62810
AT1G06790	AT1G06140	AT1G25230	AT1G63180
AT1G06840	AT1G06150	AT1G25400	AT1G63310
AT1G06890	AT1G06190	AT1G25530	AT1G63640
AT1G06900	AT1G06200	AT1G26190	AT1G64590
AT1G06950	AT1G06210	AT1G26762	AT1G64690
AT1G06960	AT1G06220	AT1G26945	AT1G66600
AT1G07080	AT1G06230	AT1G26970	AT1G66880
AT1G07110	AT1G06290	AT1G27020	AT1G66960
AT1G07120	AT1G06390	AT1G27030	AT1G67148
AT1G07220	AT1G06410	AT1G27140	AT1G67480
AT1G07300	AT1G06430	AT1G27240	AT1G67910
AT1G07350	AT1G06460	AT1G27290	AT1G67980
AT1G07360	AT1G06470	AT1G27620	AT1G68330
AT1G07380	AT1G06550	AT1G27920	AT1G68440
AT1G07420	AT1G06560	AT1G28100	AT1G69360
AT1G07470	AT1G06570	AT1G28160	AT1G69600
AT1G07473	AT1G06590	AT1G28280	AT1G69730
AT1G07476	AT1G06630	AT1G28330	AT1G69840
AT1G07480	AT1G06640	AT1G28390	AT1G69930
AT1G07510	AT1G06660	AT1G28440	AT1G70230
AT1G07530	AT1G06670	AT1G28480	AT1G70250
AT1G07615	AT1G06680	AT1G28610	AT1G70320
AT1G07650	AT1G06700	AT1G28640	AT1G70730
AT1G07670	AT1G06720	AT1G28660	AT1G70860
AT1G07705	AT1G06790	AT1G28670	AT1G71740
AT1G07710	AT1G06840	AT1G28680	AT1G71890
AT1G07730	AT1G06870	AT1G29090	AT1G72820
AT1G07740	AT1G06890	AT1G29380	AT1G72900
AT1G07745	AT1G06900	AT1G29520	AT1G72910
AT1G07810	AT1G06950	AT1G30040	AT1G72930
AT1G07840	AT1G07030	AT1G30270	AT1G73260
AT1G07890	AT1G07070	AT1G30420	AT1G73300
AT1G07910	AT1G07080	AT1G30700	AT1G73600
AT1G07920	AT1G07110	AT1G30720	AT1G73602
AT1G07930	AT1G07140	AT1G30730	AT1G73680
AT1G07940	AT1G07200	AT1G30820	AT1G73805
AT1G07960	AT1G07210	AT1G31310	AT1G74140
AT1G07970	AT1G07350	AT1G31710	AT1G74458
AT1G07990	AT1G07360	AT1G31750	AT1G74710
AT1G08000	AT1G07370	AT1G31770	AT1G74880
AT1G08010	AT1G07380	AT1G31950	AT1G74890
AT1G08030	AT1G07420	AT1G32090	AT1G76550
AT1G08040	AT1G07470	AT1G32170	AT1G76720
AT1G08060	AT1G07510	AT1G32460	AT1G76810
AT1G08110	AT1G07560	AT1G32470	AT1G76990
AT1G08125	AT1G07570	AT1G32640	AT1G77130
AT1G08190	AT1G07615	AT1G32700	AT1G77330
AT1G08200	AT1G07650	AT1G32970	AT1G77500
AT1G08210	AT1G07670	AT1G33080	AT1G78260
AT1G08220	AT1G07705	AT1G33100	AT1G78780
AT1G08260	AT1G07710	AT1G33240	AT1G79210
AT1G08270	AT1G07740	AT1G33670	AT1G79260
AT1G08300	AT1G07770	AT1G34420	AT1G79840
AT1G08360	AT1G07810	AT1G35513	AT1G80070
AT1G08370	AT1G07840	AT1G35515	AT1G80380
AT1G08410	AT1G07850	AT1G35580	AT1G80830
AT1G08420	AT1G07870	AT1G35910	AT2G01530
AT1G08450	AT1G07910	AT1G36240	AT2G02080
AT1G08480	AT1G07920	AT1G43160	AT2G02220
AT1G08490	AT1G07930	AT1G44160	AT2G02310
AT1G08510	AT1G07960	AT1G44170	AT2G02320
AT1G08520	AT1G07970	AT1G44180	AT2G02340
AT1G08550	AT1G07980	AT1G44318	AT2G02710
AT1G08570	AT1G07990	AT1G44608	AT2G02930
AT1G08600	AT1G08000	AT1G45160	AT2G02950
AT1G08620	AT1G08010	AT1G45201	AT2G03430
AT1G08640	AT1G08030	AT1G47271	AT2G03530
AT1G08660	AT1G08040	AT1G47480	AT2G03720
AT1G08680	AT1G08060	AT1G48100	AT2G04050
AT1G08720	AT1G08070	AT1G48480	AT2G04160
AT1G08750	AT1G08125	AT1G48850	AT2G04450
AT1G08760	AT1G08130	AT1G49200	AT2G05070
AT1G08800	AT1G08190	AT1G49230	AT2G05540
AT1G08820	AT1G08200	AT1G49240	AT2G07180
AT1G08830	AT1G08220	AT1G49450	AT2G07706
AT1G08840	AT1G08260	AT1G49500	AT2G13115
AT1G08970	AT1G08360	AT1G49560	AT2G13810
AT1G09010	AT1G08370	AT1G49660	AT2G13910
AT1G09020	AT1G08410	AT1G49750	AT2G15680
AT1G09050	AT1G08420	AT1G51260	AT2G15880
AT1G09060	AT1G08450	AT1G51680	AT2G16390
AT1G09100	AT1G08460	AT1G51790	AT2G16500
AT1G09130	AT1G08470	AT1G51850	AT2G16980
AT1G09140	AT1G08510	AT1G52050	AT2G17330
AT1G09150	AT1G08520	AT1G52100	AT2G17500
AT1G09160	AT1G08540	AT1G52140	AT2G17820
AT1G09210	AT1G08570	AT1G52200	AT2G18050

AT1G09230	AT1G08600	AT1G52342	AT2G18350
AT1G09270	AT1G08610	AT1G52343	AT2G18660
AT1G09280	AT1G08620	AT1G52760	AT2G18690
AT1G09290	AT1G08640	AT1G53180	AT2G18700
AT1G09310	AT1G08660	AT1G54010	AT2G18750
AT1G09330	AT1G08680	AT1G54120	AT2G18980
AT1G09490	AT1G08720	AT1G54300	AT2G20470
AT1G09570	AT1G08730	AT1G54660	AT2G20520
AT1G09580	AT1G08750	AT1G55020	AT2G20670
AT1G09590	AT1G08760	AT1G55060	AT2G20720
AT1G09610	AT1G08780	AT1G55240	AT2G20750
AT1G09620	AT1G08820	AT1G55810	AT2G21045
AT1G09630	AT1G08830	AT1G56120	AT2G21430
AT1G09640	AT1G08840	AT1G56710	AT2G21520
AT1G09645	AT1G08845	AT1G57980	AT2G21560
AT1G09660	AT1G08960	AT1G58030	AT2G21900
AT1G09700	AT1G08970	AT1G58110	AT2G22540
AT1G09730	AT1G09000	AT1G58170	AT2G22860
AT1G09760	AT1G09010	AT1G58190	AT2G22990
AT1G09770	AT1G09020	AT1G58360	AT2G23410
AT1G09810	AT1G09060	AT1G58602	AT2G23600
AT1G09840	AT1G09100	AT1G59453	AT2G24160
AT1G09870	AT1G09130	AT1G59590	AT2G24550
AT1G09910	AT1G09140	AT1G59940	AT2G24580
AT1G09920	AT1G09150	AT1G60030	AT2G24980
AT1G09940	AT1G09160	AT1G60140	AT2G25090
AT1G09980	AT1G09190	AT1G60360	AT2G25150
AT1G10130	AT1G09210	AT1G60390	AT2G25160
AT1G10170	AT1G09220	AT1G60870	AT2G25510
AT1G10180	AT1G09230	AT1G60950	AT2G25810
AT1G10240	AT1G09270	AT1G61590	AT2G26080
AT1G10290	AT1G09280	AT1G61740	AT2G26190
AT1G10320	AT1G09290	AT1G61760	AT2G26330
AT1G10390	AT1G09300	AT1G61810	AT2G26430
AT1G10410	AT1G09330	AT1G61820	AT2G27020
AT1G10430	AT1G09410	AT1G61830	AT2G28290
AT1G10450	AT1G09420	AT1G61890	AT2G28670
AT1G10490	AT1G09430	AT1G62190	AT2G28671
AT1G10510	AT1G09510	AT1G62380	AT2G28680
AT1G10570	AT1G09570	AT1G62400	AT2G29330
AT1G10590	AT1G09590	AT1G62480	AT2G29380
AT1G10610	AT1G09610	AT1G62500	AT2G30070
AT1G10630	AT1G09620	AT1G62660	AT2G30230
AT1G10660	AT1G09640	AT1G62800	AT2G30520
AT1G10670	AT1G09645	AT1G62975	AT2G30600
AT1G10700	AT1G09660	AT1G63180	AT2G30740
AT1G10730	AT1G09680	AT1G63310	AT2G30770
AT1G10760	AT1G09690	AT1G63560	AT2G31020
AT1G10820	AT1G09700	AT1G63720	AT2G31380
AT1G10840	AT1G09710	AT1G64080	AT2G31810
AT1G10865	AT1G09720	AT1G64160	AT2G31880
AT1G10870	AT1G09730	AT1G64280	AT2G32130
AT1G10900	AT1G09760	AT1G64590	AT2G32140
AT1G10930	AT1G09770	AT1G64660	AT2G32150
AT1G10940	AT1G09800	AT1G64690	AT2G32160
AT1G10950	AT1G09840	AT1G64900	AT2G32300
AT1G11000	AT1G09870	AT1G64920	AT2G32680
AT1G11060	AT1G09900	AT1G65570	AT2G33340
AT1G11130	AT1G09910	AT1G65730	AT2G33710
AT1G11200	AT1G09920	AT1G65900	AT2G33830
AT1G11280	AT1G09960	AT1G65910	AT2G34420
AT1G11350	AT1G09980	AT1G65960	AT2G34470
AT1G11390	AT1G10090	AT1G66140	AT2G34500
AT1G11400	AT1G10095	AT1G66190	AT2G34655
AT1G11480	AT1G10130	AT1G66460	AT2G34750
AT1G11650	AT1G10170	AT1G66470	AT2G35310
AT1G11660	AT1G10180	AT1G66480	AT2G35650
AT1G11680	AT1G10240	AT1G66690	AT2G36100
AT1G11720	AT1G10280	AT1G66700	AT2G36830
AT1G11750	AT1G10290	AT1G66725	AT2G37040
AT1G11755	AT1G10300	AT1G66800	AT2G37130
AT1G11760	AT1G10310	AT1G66880	AT2G37170
AT1G11840	AT1G10320	AT1G66970	AT2G37260
AT1G11860	AT1G10390	AT1G67050	AT2G37340
AT1G11880	AT1G10410	AT1G67110	AT2G37630
AT1G11890	AT1G10430	AT1G67195	AT2G37690
AT1G11900	AT1G10450	AT1G67480	AT2G37700
AT1G11910	AT1G10490	AT1G67520	AT2G37750
AT1G11960	AT1G10510	AT1G67750	AT2G38230
AT1G12000	AT1G10580	AT1G67830	AT2G38400
AT1G12050	AT1G10590	AT1G67900	AT2G38540
AT1G12120	AT1G10670	AT1G68130	AT2G39040
AT1G12210	AT1G10680	AT1G68230	AT2G39130
AT1G12230	AT1G10730	AT1G68440	AT2G39210
AT1G12280	AT1G10740	AT1G68490	AT2G39400
AT1G12290	AT1G10760	AT1G68700	AT2G39410
AT1G12300	AT1G10780	AT1G68710	AT2G39430
AT1G12360	AT1G10810	AT1G68840	AT2G39570
AT1G12410	AT1G10820	AT1G69190	AT2G39830
AT1G12430	AT1G10830	AT1G69325	AT2G40000
AT1G12470	AT1G10840	AT1G69530	AT2G40610
AT1G12520	AT1G10850	AT1G69580	AT2G40670
AT1G12580	AT1G10865	AT1G69588	AT2G40750
AT1G12640	AT1G10870	AT1G69600	AT2G41100
AT1G12650	AT1G10890	AT1G69780	AT2G41180
AT1G12680	AT1G10900	AT1G69870	AT2G41620
AT1G12730	AT1G10910	AT1G69890	AT2G41740
AT1G12750	AT1G10930	AT1G69920	AT2G42850
AT1G12770	AT1G10940	AT1G70230	AT2G43150
AT1G12780	AT1G10950	AT1G70290	AT2G43500
AT1G12840	AT1G10970	AT1G70470	AT2G43570
AT1G12920	AT1G11000	AT1G70670	AT2G43590
AT1G12930	AT1G11020	AT1G70700	AT2G43620
AT1G13000	AT1G11060	AT1G70710	AT2G43820
AT1G13050	AT1G11070	AT1G70830	AT2G45180
AT1G13060	AT1G11100	AT1G70850	AT2G45290
AT1G13120	AT1G11120	AT1G70880	AT2G45540
AT1G13150	AT1G11130	AT1G70885	AT2G45760
AT1G13160	AT1G11200	AT1G71470	AT2G46020
AT1G13170	AT1G11240	AT1G71880	AT2G46400
AT1G13190	AT1G11280	AT1G71890	AT2G46430
AT1G13195	AT1G11300	AT1G72110	AT2G46610
AT1G13220	AT1G11310	AT1G72130	AT2G46670
AT1G13270	AT1G11340	AT1G72150	AT2G46740
AT1G13320	AT1G11380	AT1G72510	AT2G46790
AT1G13330	AT1G11400	AT1G72960	AT2G46800
AT1G13340	AT1G11430	AT1G73260	AT2G46830
AT1G13350	AT1G11480	AT1G73270	AT2G47400
AT1G13560	AT1G11650	AT1G73280	AT2G47450
AT1G13570	AT1G11660	AT1G73300	AT2G47510
AT1G13730	AT1G11680	AT1G73370	AT2G48020
AT1G13770	AT1G11710	AT1G73610	AT2G48110
AT1G13860	AT1G11720	AT1G73620	AT3G01190

AT1G13870	AT1G11750	AT1G73805	AT3G01290
AT1G13940	AT1G11790	AT1G73850	AT3G01345
AT1G13980	AT1G11820	AT1G74000	AT3G01850
AT1G13990	AT1G11840	AT1G74020	AT3G02260
AT1G14000	AT1G11870	AT1G74055	AT3G02468
AT1G14090	AT1G11890	AT1G74458	AT3G02470
AT1G14180	AT1G11905	AT1G74710	AT3G02770
AT1G14300	AT1G11910	AT1G74740	AT3G03380
AT1G14320	AT1G11960	AT1G74770	AT3G03450
AT1G14340	AT1G12000	AT1G74840	AT3G04210
AT1G14380	AT1G12050	AT1G74880	AT3G06390
AT1G14400	AT1G12120	AT1G74890	AT3G06460
AT1G14480	AT1G12270	AT1G75000	AT3G06880
AT1G14510	AT1G12290	AT1G75240	AT3G07660
AT1G14560	AT1G12350	AT1G75260	AT3G08940
AT1G14570	AT1G12360	AT1G75388	AT3G09220
AT1G14590	AT1G12370	AT1G75390	AT3G09840
AT1G14610	AT1G12470	AT1G75430	AT3G09940
AT1G14650	AT1G12520	AT1G75440	AT3G10340
AT1G14670	AT1G12530	AT1G75450	AT3G10720
AT1G14690	AT1G12580	AT1G75500	AT3G10780
AT1G14710	AT1G12680	AT1G75700	AT3G10910
AT1G14830	AT1G12760	AT1G75720	AT3G10912
AT1G14850	AT1G12770	AT1G75780	AT3G10960
AT1G14910	AT1G12775	AT1G76090	AT3G11270
AT1G14970	AT1G12820	AT1G76100	AT3G11340
AT1G15020	AT1G12850	AT1G76240	AT3G11550
AT1G15040	AT1G12910	AT1G76250	AT3G11720
AT1G15060	AT1G12920	AT1G76410	AT3G12220
AT1G15110	AT1G12930	AT1G76420	AT3G12230
AT1G15120	AT1G13000	AT1G76490	AT3G12240
AT1G15125	AT1G13020	AT1G76550	AT3G12540
AT1G15200	AT1G13050	AT1G76590	AT3G13090
AT1G15215	AT1G13060	AT1G76660	AT3G13100
AT1G15240	AT1G13120	AT1G76880	AT3G13130
AT1G15270	AT1G13160	AT1G76952	AT3G13235
AT1G15290	AT1G13170	AT1G76990	AT3G13610
AT1G15340	AT1G13180	AT1G77380	AT3G13630
AT1G15370	AT1G13190	AT1G77500	AT3G13810
AT1G15420	AT1G13195	AT1G77700	AT3G14530
AT1G15430	AT1G13210	AT1G77760	AT3G14550
AT1G15440	AT1G13220	AT1G77885	AT3G14620
AT1G15490	AT1G13320	AT1G78260	AT3G15020
AT1G15570	AT1G13350	AT1G78370	AT3G15353
AT1G15690	AT1G13440	AT1G78390	AT3G15450
AT1G15740	AT1G13560	AT1G78580	AT3G16800
AT1G15750	AT1G13730	AT1G78700	AT3G17690
AT1G15780	AT1G13770	AT1G78780	AT3G18670
AT1G15860	AT1G13860	AT1G78830	AT3G18750
AT1G15880	AT1G13880	AT1G78850	AT3G18860
AT1G15910	AT1G13940	AT1G79245	AT3G20570
AT1G15930	AT1G13960	AT1G79420	AT3G20680
AT1G15940	AT1G13980	AT1G79430	AT3G21000
AT1G16010	AT1G14000	AT1G79480	AT3G21080
AT1G16020	AT1G14010	AT1G79520	AT3G21090
AT1G16090	AT1G14030	AT1G80050	AT3G21520
AT1G16180	AT1G14100	AT1G80160	AT3G21710
AT1G16190	AT1G14140	AT1G80280	AT3G22200
AT1G16240	AT1G14230	AT1G80660	AT3G22235
AT1G16270	AT1G14290	AT1G80920	AT3G22240
AT1G16300	AT1G14300	AT2G01021	AT3G22400
AT1G16470	AT1G14320	AT2G01275	AT3G22910
AT1G16480	AT1G14360	AT2G01530	AT3G23080
AT1G16490	AT1G14370	AT2G01770	AT3G23175
AT1G16520	AT1G14380	AT2G01830	AT3G23240
AT1G16540	AT1G14470	AT2G01890	AT3G23530
AT1G16560	AT1G14500	AT2G01950	AT3G23880
AT1G16570	AT1G14570	AT2G02060	AT3G24020
AT1G16610	AT1G14610	AT2G02070	AT3G24170
AT1G16650	AT1G14650	AT2G02080	AT3G24180
AT1G16670	AT1G14670	AT2G02310	AT3G24750
AT1G16680	AT1G14690	AT2G02710	AT3G25020
AT1G16690	AT1G14710	AT2G02950	AT3G25190
AT1G16700	AT1G14740	AT2G03090	AT3G25717
AT1G16710	AT1G14790	AT2G04110	AT3G25882
AT1G16780	AT1G14810	AT2G04160	AT3G25930
AT1G16800	AT1G14830	AT2G04170	AT3G26290
AT1G16810	AT1G14850	AT2G04400	AT3G26320
AT1G16858	AT1G14910	AT2G04690	AT3G26340
AT1G16860	AT1G14970	AT2G05400	AT3G26720
AT1G16870	AT1G15020	AT2G05440	AT3G27040
AT1G16890	AT1G15030	AT2G05441	AT3G27070
AT1G16900	AT1G15060	AT2G05510	AT3G27940
AT1G16920	AT1G15110	AT2G05530	AT3G28040
AT1G17040	AT1G15125	AT2G05910	AT3G28210
AT1G17050	AT1G15130	AT2G07050	AT3G28510
AT1G17070	AT1G15200	AT2G07180	AT3G28550
AT1G17080	AT1G15270	AT2G07706	AT3G28600
AT1G17110	AT1G15280	AT2G09970	AT3G28740
AT1G17130	AT1G15290	AT2G11620	AT3G28899
AT1G17145	AT1G15310	AT2G13360	AT3G29240
AT1G17210	AT1G15340	AT2G14560	AT3G29430
AT1G17250	AT1G15370	AT2G14620	AT3G29635
AT1G17280	AT1G15415	AT2G14740	AT3G30720
AT1G17290	AT1G15420	AT2G15220	AT3G30841
AT1G17350	AT1G15440	AT2G15680	AT3G33528
AT1G17360	AT1G15480	AT2G15880	AT3G42640
AT1G17370	AT1G15500	AT2G15960	AT3G44340
AT1G17440	AT1G15570	AT2G16005	AT3G44400
AT1G17450	AT1G15690	AT2G16190	AT3G44630
AT1G17460	AT1G15740	AT2G16360	AT3G44670
AT1G17470	AT1G15750	AT2G16400	AT3G44860
AT1G17500	AT1G15780	AT2G16500	AT3G44870
AT1G17520	AT1G15810	AT2G16890	AT3G45070
AT1G17580	AT1G15880	AT2G16980	AT3G45410
AT1G17650	AT1G15910	AT2G17330	AT3G45650
AT1G17680	AT1G15930	AT2G17370	AT3G45700
AT1G17690	AT1G15940	AT2G17440	AT3G45710
AT1G17720	AT1G15950	AT2G17500	AT3G45780
AT1G17760	AT1G15970	AT2G17550	AT3G46080
AT1G17820	AT1G16000	AT2G17820	AT3G46090
AT1G17870	AT1G16040	AT2G17950	AT3G46600
AT1G17880	AT1G16090	AT2G18050	AT3G46700
AT1G17930	AT1G16170	AT2G18150	AT3G47090
AT1G17940	AT1G16180	AT2G18160	AT3G47210
AT1G17980	AT1G16190	AT2G18162	AT3G47340
AT1G18030	AT1G16220	AT2G18280	AT3G47380
AT1G18070	AT1G16240	AT2G18350	AT3G47470
AT1G18150	AT1G16260	AT2G18500	AT3G47500
AT1G18160	AT1G16270	AT2G18590	AT3G47540
AT1G18190	AT1G16280	AT2G18700	AT3G47600
AT1G18270	AT1G16300	AT2G19110	AT3G47770
AT1G18335	AT1G16330	AT2G19800	AT3G47910

AT1G18340	AT1G16380	AT2G20080	AT3G47960
AT1G18415	AT1G16460	AT2G20362	AT3G48080
AT1G18420	AT1G16470	AT2G20670	AT3G48090
AT1G18450	AT1G16480	AT2G20750	AT3G48360
AT1G18460	AT1G16540	AT2G21140	AT3G48380
AT1G18470	AT1G16560	AT2G21330	AT3G48390
AT1G18530	AT1G16570	AT2G21560	AT3G48640
AT1G18580	AT1G16610	AT2G22125	AT3G48650
AT1G18610	AT1G16670	AT2G22330	AT3G49960
AT1G18630	AT1G16680	AT2G22660	AT3G50170
AT1G18640	AT1G16710	AT2G22730	AT3G50480
AT1G18660	AT1G16770	AT2G22840	AT3G50560
AT1G18700	AT1G16780	AT2G22850	AT3G50640
AT1G18800	AT1G16800	AT2G22860	AT3G51690
AT1G18840	AT1G16810	AT2G22930	AT3G51840
AT1G18850	AT1G16858	AT2G22980	AT3G52340
AT1G18900	AT1G16870	AT2G22990	AT3G52430
AT1G18950	AT1G16890	AT2G23300	AT3G52910
AT1G19100	AT1G16900	AT2G23400	AT3G53150
AT1G19110	AT1G16920	AT2G23410	AT3G53180
AT1G19170	AT1G16960	AT2G23560	AT3G53230
AT1G19220	AT1G16970	AT2G23950	AT3G53420
AT1G19270	AT1G17050	AT2G23960	AT3G53800
AT1G19290	AT1G17070	AT2G24260	AT3G54500
AT1G19310	AT1G17080	AT2G24550	AT3G54580
AT1G19360	AT1G17110	AT2G24580	AT3G54590
AT1G19397	AT1G17200	AT2G24790	AT3G54770
AT1G19400	AT1G17210	AT2G24850	AT3G55230
AT1G19485	AT1G17220	AT2G25090	AT3G55470
AT1G19580	AT1G17250	AT2G25150	AT3G55610
AT1G19680	AT1G17260	AT2G25160	AT3G55970
AT1G19715	AT1G17280	AT2G25180	AT3G56080
AT1G19800	AT1G17290	AT2G25590	AT3G56400
AT1G19850	AT1G17360	AT2G25810	AT3G56710
AT1G19860	AT1G17370	AT2G25900	AT3G57460
AT1G19870	AT1G17440	AT2G26400	AT3G57520
AT1G19880	AT1G17450	AT2G26440	AT3G57950
AT1G20090	AT1G17460	AT2G26480	AT3G59370
AT1G20200	AT1G17470	AT2G26640	AT3G59480
AT1G20220	AT1G17480	AT2G26700	AT3G59570
AT1G20260	AT1G17500	AT2G27000	AT3G59710
AT1G20410	AT1G17510	AT2G27010	AT3G60160
AT1G20460	AT1G17520	AT2G27140	AT3G60420
AT1G20470	AT1G17550	AT2G27300	AT3G60470
AT1G20540	AT1G17560	AT2G27930	AT3G60966
AT1G20575	AT1G17580	AT2G28510	AT3G60970
AT1G20580	AT1G17590	AT2G28570	AT3G61280
AT1G20620	AT1G17600	AT2G28610	AT3G61430
AT1G20630	AT1G17630	AT2G28670	AT3G62570
AT1G20640	AT1G17680	AT2G28671	AT3G62680
AT1G20670	AT1G17690	AT2G28680	AT3G63110
AT1G20693	AT1G17720	AT2G28810	AT3G63520
AT1G20760	AT1G17750	AT2G29410	AT4G00380
AT1G20770	AT1G17760	AT2G29450	AT4G00780
AT1G20780	AT1G17820	AT2G29660	AT4G01140
AT1G20910	AT1G17850	AT2G29710	AT4G01330
AT1G20950	AT1G17880	AT2G29750	AT4G01600
AT1G20960	AT1G17930	AT2G29970	AT4G02120
AT1G20970	AT1G17980	AT2G30490	AT4G02270
AT1G20980	AT1G18030	AT2G30520	AT4G02733
AT1G21080	AT1G18040	AT2G30540	AT4G03420
AT1G21160	AT1G18050	AT2G30600	AT4G03440
AT1G21170	AT1G18070	AT2G30750	AT4G03470
AT1G21340	AT1G18080	AT2G30770	AT4G03560
AT1G21380	AT1G18160	AT2G30860	AT4G03630
AT1G21450	AT1G18190	AT2G30930	AT4G04220
AT1G21480	AT1G18335	AT2G31020	AT4G04570
AT1G21560	AT1G18440	AT2G31030	AT4G04620
AT1G21580	AT1G18450	AT2G31180	AT4G04710
AT1G21610	AT1G18460	AT2G31380	AT4G05030
AT1G21630	AT1G18470	AT2G31540	AT4G06746
AT1G21640	AT1G18480	AT2G31550	AT4G08040
AT1G21690	AT1G18485	AT2G31750	AT4G08093
AT1G21730	AT1G18500	AT2G31800	AT4G08290
AT1G21760	AT1G18530	AT2G31810	AT4G08400
AT1G21930	AT1G18540	AT2G31880	AT4G08410
AT1G22040	AT1G18610	AT2G32140	AT4G09770
AT1G22060	AT1G18640	AT2G32150	AT4G09960
AT1G22070	AT1G18660	AT2G32160	AT4G11210
AT1G22200	AT1G18680	AT2G32460	AT4G11290
AT1G22300	AT1G18700	AT2G32510	AT4G11320
AT1G22450	AT1G18800	AT2G32680	AT4G11340
AT1G22510	AT1G18840	AT2G32750	AT4G11740
AT1G22620	AT1G18850	AT2G33080	AT4G11890
AT1G22660	AT1G18890	AT2G33230	AT4G12510
AT1G22730	AT1G18900	AT2G33780	AT4G12520
AT1G22770	AT1G18910	AT2G33830	AT4G12545
AT1G22780	AT1G18950	AT2G33850	AT4G12550
AT1G22790	AT1G19000	AT2G34010	AT4G13390
AT1G22860	AT1G19100	AT2G34060	AT4G13580
AT1G22930	AT1G19110	AT2G34500	AT4G14365
AT1G23180	AT1G19220	AT2G34530	AT4G14390
AT1G23190	AT1G19270	AT2G34540	AT4G14940
AT1G23230	AT1G19310	AT2G34590	AT4G15165
AT1G23260	AT1G19350	AT2G34610	AT4G15230
AT1G23280	AT1G19430	AT2G34650	AT4G15340
AT1G23310	AT1G19485	AT2G34655	AT4G15350
AT1G23450	AT1G19520	AT2G34680	AT4G15390
AT1G23460	AT1G19580	AT2G34750	AT4G15393
AT1G23470	AT1G19600	AT2G34790	AT4G15420
AT1G23490	AT1G19690	AT2G34940	AT4G15670
AT1G23870	AT1G19720	AT2G35940	AT4G15690
AT1G23900	AT1G19800	AT2G35980	AT4G15990
AT1G23940	AT1G19850	AT2G36080	AT4G16000
AT1G23960	AT1G19870	AT2G36100	AT4G16008
AT1G24030	AT1G19880	AT2G36380	AT4G16190
AT1G24120	AT1G20100	AT2G36400	AT4G16480
AT1G24160	AT1G20110	AT2G36880	AT4G16760
AT1G24190	AT1G20200	AT2G37040	AT4G16860
AT1G24265	AT1G20220	AT2G37130	AT4G16890
AT1G24267	AT1G20260	AT2G37170	AT4G16920
AT1G24300	AT1G20270	AT2G37180	AT4G16950
AT1G24360	AT1G20370	AT2G37260	AT4G17330
AT1G24440	AT1G20380	AT2G37300	AT4G17970
AT1G24460	AT1G20410	AT2G37390	AT4G18020
AT1G24490	AT1G20460	AT2G37450	AT4G18250
AT1G24510	AT1G20540	AT2G37590	AT4G18510
AT1G24600	AT1G20550	AT2G37610	AT4G18880
AT1G24610	AT1G20575	AT2G37710	AT4G18910
AT1G24706	AT1G20580	AT2G37925	AT4G18920
AT1G24764	AT1G20630	AT2G38040	AT4G19410
AT1G25260	AT1G20640	AT2G38210	AT4G19520
AT1G25280	AT1G20670	AT2G38230	AT4G19720

AT1G25350	AT1G20696	AT2G38240	AT4G19840
AT1G25380	AT1G20760	AT2G38290	AT4G19920
AT1G25490	AT1G20770	AT2G38750	AT4G19925
AT1G25540	AT1G20780	AT2G38760	AT4G20070
AT1G25570	AT1G20830	AT2G38800	AT4G20110
AT1G25580	AT1G20840	AT2G39040	AT4G20140
AT1G25682	AT1G20910	AT2G39130	AT4G20320
AT1G26110	AT1G20920	AT2G39210	AT4G20420
AT1G26120	AT1G20950	AT2G39220	AT4G20460
AT1G26160	AT1G20960	AT2G39410	AT4G20850
AT1G26170	AT1G20970	AT2G39430	AT4G21120
AT1G26190	AT1G20980	AT2G39570	AT4G21340
AT1G26208	AT1G21080	AT2G39705	AT4G21380
AT1G26210	AT1G21090	AT2G39710	AT4G21640
AT1G26270	AT1G21160	AT2G39830	AT4G21650
AT1G26290	AT1G21170	AT2G39980	AT4G21660
AT1G26300	AT1G21380	AT2G40110	AT4G21750
AT1G26370	AT1G21480	AT2G40130	AT4G21830
AT1G26440	AT1G21580	AT2G40200	AT4G21840
AT1G26550	AT1G21600	AT2G40230	AT4G21903
AT1G26580	AT1G21610	AT2G40260	AT4G22050
AT1G26620	AT1G21630	AT2G40330	AT4G22080
AT1G26630	AT1G21650	AT2G40390	AT4G22214
AT1G26640	AT1G21660	AT2G40610	AT4G22666
AT1G26660	AT1G21700	AT2G40670	AT4G23030
AT1G26665	AT1G21720	AT2G40890	AT4G23220
AT1G26670	AT1G21730	AT2G40970	AT4G23250
AT1G26810	AT1G21740	AT2G41300	AT4G23260
AT1G26830	AT1G21980	AT2G41310	AT4G23270
AT1G26850	AT1G22020	AT2G41330	AT4G23570
AT1G26880	AT1G22060	AT2G41660	AT4G23990
AT1G26900	AT1G22070	AT2G42380	AT4G24630
AT1G26910	AT1G22180	AT2G42620	AT4G24670
AT1G26940	AT1G22410	AT2G42820	AT4G24690
AT1G27000	AT1G22450	AT2G42850	AT4G25050
AT1G27110	AT1G22520	AT2G42890	AT4G25170
AT1G27150	AT1G22650	AT2G43150	AT4G25400
AT1G27190	AT1G22710	AT2G43230	AT4G25820
AT1G27320	AT1G22780	AT2G43470	AT4G25940
AT1G27340	AT1G22850	AT2G43480	AT4G26010
AT1G27350	AT1G22860	AT2G43500	AT4G26070
AT1G27360	AT1G22870	AT2G43510	AT4G26090
AT1G27370	AT1G22920	AT2G43550	AT4G26200
AT1G27385	AT1G22930	AT2G43590	AT4G26220
AT1G27390	AT1G22950	AT2G43620	AT4G26260
AT1G27400	AT1G23030	AT2G43680	AT4G26320
AT1G27420	AT1G23180	AT2G43820	AT4G26970
AT1G27430	AT1G23190	AT2G43870	AT4G27310
AT1G27470	AT1G23230	AT2G43880	AT4G27870
AT1G27500	AT1G23260	AT2G43910	AT4G28085
AT1G27590	AT1G23280	AT2G44070	AT4G28190
AT1G27595	AT1G23310	AT2G44080	AT4G28290
AT1G27630	AT1G23450	AT2G44130	AT4G28490
AT1G27650	AT1G23490	AT2G44160	AT4G28650
AT1G27700	AT1G23820	AT2G44370	AT4G28860
AT1G27750	AT1G23830	AT2G44670	AT4G29900
AT1G27752	AT1G23880	AT2G44790	AT4G29990
AT1G27770	AT1G23900	AT2G45160	AT4G30110
AT1G27840	AT1G24050	AT2G45170	AT4G30170
AT1G27850	AT1G24159	AT2G45220	AT4G30250
AT1G27900	AT1G24180	AT2G45430	AT4G30720
AT1G27920	AT1G24190	AT2G45480	AT4G31290
AT1G27921	AT1G24240	AT2G45910	AT4G32212
AT1G27960	AT1G24265	AT2G46330	AT4G32730
AT1G28090	AT1G24267	AT2G46430	AT4G32860
AT1G28120	AT1G24280	AT2G46440	AT4G33050
AT1G28210	AT1G24300	AT2G46494	AT4G33730
AT1G28240	AT1G24310	AT2G46620	AT4G34135
AT1G28320	AT1G24340	AT2G46630	AT4G34220
AT1G28350	AT1G24360	AT2G46660	AT4G34390
AT1G28380	AT1G24440	AT2G46890	AT4G34580
AT1G28395	AT1G24490	AT2G47180	AT4G34890
AT1G28410	AT1G24510	AT2G47400	AT4G35800
AT1G28420	AT1G24560	AT2G47450	AT4G36220
AT1G28490	AT1G24706	AT2G47500	AT4G36790
AT1G28510	AT1G25260	AT2G47560	AT4G36880
AT1G28520	AT1G25280	AT2G47800	AT4G37010
AT1G28550	AT1G25350	AT2G48020	AT4G37220
AT1G28560	AT1G25360	AT3G01175	AT4G37300
AT1G28710	AT1G25380	AT3G01220	AT4G37520
AT1G28720	AT1G25420	AT3G01260	AT4G37610
AT1G28730	AT1G25490	AT3G01290	AT4G38440
AT1G28740	AT1G25540	AT3G01513	AT4G38470
AT1G28750	AT1G25570	AT3G01670	AT4G38540
AT1G28770	AT1G25580	AT3G01680	AT4G38550
AT1G28780	AT1G25682	AT3G01940	AT4G39030
AT1G28790	AT1G26110	AT3G01950	AT4G39640
AT1G28800	AT1G26120	AT3G01990	AT4G39660
AT1G28810	AT1G26160	AT3G02140	AT4G39740
AT1G28820	AT1G26170	AT3G02170	AT4G39770
AT1G28830	AT1G26180	AT3G02468	AT4G39850
AT1G28840	AT1G26190	AT3G02470	AT5G01210
AT1G28850	AT1G26290	AT3G02885	AT5G01250
AT1G28860	AT1G26300	AT3G03200	AT5G01550
AT1G28870	AT1G26370	AT3G03240	AT5G01810
AT1G28880	AT1G26460	AT3G03440	AT5G01850
AT1G28890	AT1G26500	AT3G03450	AT5G01900
AT1G28900	AT1G26550	AT3G03520	AT5G02020
AT1G28910	AT1G26630	AT3G03770	AT5G02030
AT1G28920	AT1G26660	AT3G03780	AT5G02090
AT1G28930	AT1G26670	AT3G03990	AT5G02100
AT1G28940	AT1G26690	AT3G04000	AT5G02550
AT1G28950	AT1G26730	AT3G04070	AT5G02840
AT1G28970	AT1G26740	AT3G04940	AT5G02880
AT1G28980	AT1G26750	AT3G05020	AT5G03240
AT1G28990	AT1G26830	AT3G05165	AT5G03860
AT1G29030	AT1G26850	AT3G05620	AT5G04470
AT1G29120	AT1G26880	AT3G05640	AT5G04890
AT1G29150	AT1G26900	AT3G05650	AT5G04960
AT1G29170	AT1G27000	AT3G05770	AT5G05040
AT1G29220	AT1G27008	AT3G05936	AT5G05060
AT1G29250	AT1G27070	AT3G06460	AT5G05170
AT1G29310	AT1G27090	AT3G06880	AT5G05340
AT1G29320	AT1G27150	AT3G07130	AT5G05460
AT1G29330	AT1G27190	AT3G07320	AT5G05500
AT1G29340	AT1G27300	AT3G07340	AT5G05890
AT1G29350	AT1G27310	AT3G07420	AT5G05965
AT1G29370	AT1G27320	AT3G07870	AT5G06043
AT1G29380	AT1G27340	AT3G09020	AT5G06200
AT1G29400	AT1G27360	AT3G09070	AT5G06630
AT1G29410	AT1G27370	AT3G09220	AT5G06640
AT1G29470	AT1G27385	AT3G09250	AT5G06980
AT1G29540	AT1G27390	AT3G09260	AT5G07350

AT1G29630	AT1G27400	AT3G09270	AT5G07550
AT1G29690	AT1G27420	AT3G09405	AT5G07740
AT1G29750	AT1G27430	AT3G09940	AT5G07770
AT1G29770	AT1G27435	AT3G10113	AT5G08170
AT1G29780	AT1G27440	AT3G10340	AT5G08240
AT1G29800	AT1G27450	AT3G10680	AT5G10140
AT1G29810	AT1G27460	AT3G10740	AT5G10210
AT1G29850	AT1G27470	AT3G10960	AT5G11320
AT1G29880	AT1G27595	AT3G11280	AT5G11570
AT1G29890	AT1G27600	AT3G11340	AT5G11920
AT1G29940	AT1G27630	AT3G11550	AT5G13010
AT1G29951	AT1G27750	AT3G11930	AT5G13210
AT1G29952	AT1G27752	AT3G12090	AT5G13320
AT1G30000	AT1G27760	AT3G12700	AT5G13490
AT1G30010	AT1G27770	AT3G12890	AT5G13590
AT1G30020	AT1G27840	AT3G12910	AT5G13650
AT1G30070	AT1G27850	AT3G12977	AT5G13710
AT1G30090	AT1G27900	AT3G13080	AT5G14120
AT1G30120	AT1G27910	AT3G13100	AT5G14330
AT1G30230	AT1G27960	AT3G13130	AT5G14610
AT1G30240	AT1G27970	AT3G13590	AT5G14780
AT1G30270	AT1G28060	AT3G13610	AT5G14880
AT1G30300	AT1G28090	AT3G13620	AT5G15150
AT1G30330	AT1G28100	AT3G13730	AT5G15310
AT1G30360	AT1G28110	AT3G13750	AT5G15725
AT1G30400	AT1G28120	AT3G13980	AT5G15948
AT1G30420	AT1G28240	AT3G14050	AT5G15950
AT1G30440	AT1G28320	AT3G14520	AT5G16570
AT1G30450	AT1G28340	AT3G14540	AT5G16900
AT1G30470	AT1G28350	AT3G14550	AT5G17920
AT1G30490	AT1G28395	AT3G14650	AT5G18670
AT1G30500	AT1G28420	AT3G14660	AT5G19230
AT1G30570	AT1G28510	AT3G14940	AT5G19250
AT1G30580	AT1G28520	AT3G15170	AT5G19600
AT1G30590	AT1G28530	AT3G15353	AT5G20000
AT1G30600	AT1G28540	AT3G15440	AT5G20250
AT1G30620	AT1G28580	AT3G15450	AT5G20350
AT1G30630	AT1G28815	AT3G15470	AT5G20420
AT1G30640	AT1G28960	AT3G15630	AT5G20670
AT1G30680	AT1G29030	AT3G15680	AT5G20910
AT1G30820	AT1G29120	AT3G15760	AT5G20960
AT1G30825	AT1G29150	AT3G15800	AT5G20970
AT1G30890	AT1G29200	AT3G15810	AT5G22020
AT1G30910	AT1G29220	AT3G15820	AT5G22300
AT1G30970	AT1G29250	AT3G15950	AT5G22410
AT1G31120	AT1G29310	AT3G16390	AT5G23360
AT1G31150	AT1G29320	AT3G16400	AT5G23380
AT1G31190	AT1G29330	AT3G16410	AT5G23570
AT1G31200	AT1G29350	AT3G16440	AT5G23660
AT1G31230	AT1G29370	AT3G16460	AT5G23670
AT1G31280	AT1G29395	AT3G16500	AT5G23860
AT1G31300	AT1G29400	AT3G16520	AT5G23890
AT1G31340	AT1G29470	AT3G16530	AT5G24070
AT1G31360	AT1G29690	AT3G16770	AT5G24120
AT1G31420	AT1G29710	AT3G16850	AT5G24200
AT1G31440	AT1G29770	AT3G17609	AT5G24210
AT1G31480	AT1G29810	AT3G17690	AT5G24300
AT1G31485	AT1G29820	AT3G17730	AT5G24620
AT1G31490	AT1G29850	AT3G17810	AT5G24690
AT1G31500	AT1G29880	AT3G17860	AT5G24810
AT1G31600	AT1G29890	AT3G18260	AT5G25250
AT1G31660	AT1G29900	AT3G18670	AT5G25260
AT1G31770	AT1G29940	AT3G18773	AT5G25350
AT1G31780	AT1G29950	AT3G18780	AT5G25890
AT1G31810	AT1G29965	AT3G18830	AT5G26690
AT1G31817	AT1G29970	AT3G18950	AT5G26920
AT1G31850	AT1G30000	AT3G19040	AT5G28080
AT1G31860	AT1G30120	AT3G19270	AT5G28770
AT1G31870	AT1G30210	AT3G19450	AT5G35190
AT1G31900	AT1G30240	AT3G19820	AT5G35690
AT1G31910	AT1G30270	AT3G19930	AT5G36180
AT1G31930	AT1G30330	AT3G20110	AT5G36870
AT1G31970	AT1G30360	AT3G20340	AT5G37180
AT1G32050	AT1G30400	AT3G20570	AT5G38200
AT1G32130	AT1G30410	AT3G20660	AT5G38350
AT1G32150	AT1G30440	AT3G20940	AT5G38990
AT1G32160	AT1G30450	AT3G21080	AT5G39050
AT1G32200	AT1G30455	AT3G21210	AT5G39080
AT1G32210	AT1G30460	AT3G21240	AT5G39110
AT1G32230	AT1G30470	AT3G21351	AT5G39120
AT1G32330	AT1G30480	AT3G21510	AT5G39150
AT1G32370	AT1G30490	AT3G21520	AT5G39180
AT1G32380	AT1G30540	AT3G21530	AT5G39580
AT1G32390	AT1G30580	AT3G21600	AT5G39660
AT1G32400	AT1G30590	AT3G21670	AT5G39670
AT1G32410	AT1G30600	AT3G21680	AT5G40000
AT1G32415	AT1G30620	AT3G21690	AT5G40450
AT1G32440	AT1G30630	AT3G21700	AT5G40580
AT1G32460	AT1G30680	AT3G21710	AT5G40730
AT1G32490	AT1G30810	AT3G21770	AT5G41290
AT1G32530	AT1G30820	AT3G22060	AT5G41300
AT1G32583	AT1G30870	AT3G22200	AT5G41700
AT1G32750	AT1G30960	AT3G22235	AT5G41740
AT1G32790	AT1G30970	AT3G22240	AT5G41750
AT1G32810	AT1G31010	AT3G22400	AT5G42180
AT1G32930	AT1G31220	AT3G22890	AT5G42235
AT1G32940	AT1G31230	AT3G23080	AT5G42240
AT1G33060	AT1G31300	AT3G23120	AT5G42580
AT1G33120	AT1G31340	AT3G23410	AT5G42830
AT1G33140	AT1G31360	AT3G23820	AT5G42950
AT1G33160	AT1G31420	AT3G24020	AT5G43000
AT1G33230	AT1G31430	AT3G24180	AT5G43470
AT1G33390	AT1G31440	AT3G24210	AT5G44417
AT1G33400	AT1G31480	AT3G24750	AT5G44572
AT1G33410	AT1G31490	AT3G24770	AT5G44585
AT1G33470	AT1G31500	AT3G25560	AT5G44680
AT1G33660	AT1G31600	AT3G25585	AT5G45110
AT1G33680	AT1G31650	AT3G25597	AT5G45200
AT1G33780	AT1G31660	AT3G25810	AT5G45380
AT1G33980	AT1G31730	AT3G25855	AT5G45490
AT1G34020	AT1G31780	AT3G25860	AT5G45500
AT1G34030	AT1G31790	AT3G26165	AT5G45800
AT1G34065	AT1G31800	AT3G26220	AT5G46180
AT1G34120	AT1G31810	AT3G26320	AT5G46510
AT1G34130	AT1G31850	AT3G26450	AT5G46520
AT1G34150	AT1G31860	AT3G26460	AT5G47130
AT1G34190	AT1G31870	AT3G26470	AT5G47560
AT1G34210	AT1G31910	AT3G26520	AT5G47950
AT1G34220	AT1G31920	AT3G26570	AT5G47980
AT1G34260	AT1G31970	AT3G26720	AT5G48100
AT1G34300	AT1G32050	AT3G26740	AT5G48160
AT1G34320	AT1G32090	AT3G27090	AT5G48350
AT1G34360	AT1G32130	AT3G27390	AT5G48390

AT1G34420	AT1G32150	AT3G27970	AT5G48400
AT1G34470	AT1G32190	AT3G27980	AT5G48620
AT1G34550	AT1G32210	AT3G28040	AT5G49360
AT1G34630	AT1G32230	AT3G28180	AT5G49448
AT1G35220	AT1G32330	AT3G28540	AT5G49450
AT1G35470	AT1G32360	AT3G28570	AT5G49760
AT1G35510	AT1G32400	AT3G28580	AT5G50140
AT1G35580	AT1G32415	AT3G28600	AT5G50600
AT1G35620	AT1G32440	AT3G28650	AT5G50700
AT1G35660	AT1G32460	AT3G28890	AT5G51060
AT1G36050	AT1G32490	AT3G28899	AT5G51340
AT1G36070	AT1G32500	AT3G29035	AT5G52250
AT1G36160	AT1G32530	AT3G29240	AT5G52540
AT1G36180	AT1G32580	AT3G29250	AT5G52570
AT1G36280	AT1G32750	AT3G29430	AT5G52680
AT1G36320	AT1G32790	AT3G29575	AT5G52700
AT1G36370	AT1G32810	AT3G29670	AT5G52710
AT1G36730	AT1G32990	AT3G29770	AT5G52720
AT1G36732	AT1G33040	AT3G30390	AT5G52730
AT1G36980	AT1G33060	AT3G30775	AT5G52750
AT1G36990	AT1G33120	AT3G32030	AT5G52760
AT1G41880	AT1G33230	AT3G43430	AT5G52810
AT1G42440	AT1G33265	AT3G44300	AT5G53250
AT1G42470	AT1G33390	AT3G44310	AT5G53460
AT1G42480	AT1G33400	AT3G44320	AT5G53500
AT1G42540	AT1G33410	AT3G44326	AT5G53870
AT1G42960	AT1G33420	AT3G44550	AT5G54710
AT1G43130	AT1G33470	AT3G44630	AT5G55250
AT1G43170	AT1G33480	AT3G44860	AT5G56370
AT1G43190	AT1G33490	AT3G44960	AT5G56540
AT1G43580	AT1G33680	AT3G44970	AT5G56700
AT1G43620	AT1G33980	AT3G45070	AT5G56870
AT1G43670	AT1G34030	AT3G45080	AT5G56890
AT1G43690	AT1G34065	AT3G45310	AT5G56960
AT1G43710	AT1G34110	AT3G45410	AT5G57350
AT1G43770	AT1G34130	AT3G45610	AT5G57580
AT1G43850	AT1G34140	AT3G45680	AT5G57625
AT1G43860	AT1G34150	AT3G45700	AT5G57660
AT1G43890	AT1G34160	AT3G45710	AT5G57920
AT1G43980	AT1G34190	AT3G45780	AT5G59080
AT1G44125	AT1G34200	AT3G45800	AT5G59090
AT1G44750	AT1G34210	AT3G46280	AT5G59440
AT1G44770	AT1G34220	AT3G46520	AT5G59780
AT1G44780	AT1G34260	AT3G46650	AT5G59810
AT1G44835	AT1G34270	AT3G46690	AT5G59940
AT1G44910	AT1G34300	AT3G46700	AT5G60100
AT1G45000	AT1G34320	AT3G46720	AT5G60120
AT1G45120	AT1G34430	AT3G47180	AT5G60610
AT1G45150	AT1G34470	AT3G47340	AT5G61260
AT1G45160	AT1G34550	AT3G47380	AT5G61410
AT1G45170	AT1G34630	AT3G47470	AT5G61490
AT1G45180	AT1G34770	AT3G47560	AT5G61495
AT1G45207	AT1G34780	AT3G47710	AT5G61510
AT1G45229	AT1G35220	AT3G47960	AT5G61900
AT1G45231	AT1G35460	AT3G48100	AT5G62680
AT1G45234	AT1G35470	AT3G48360	AT5G62740
AT1G45236	AT1G35510	AT3G48390	AT5G63225
AT1G45238	AT1G35580	AT3G48640	AT5G63600
AT1G45240	AT1G35720	AT3G48650	AT5G63660
AT1G45242	AT1G36160	AT3G49110	AT5G64060
AT1G45243	AT1G36280	AT3G49120	AT5G64100
AT1G45244	AT1G36310	AT3G49160	AT5G64120
AT1G45246	AT1G36370	AT3G49780	AT5G64570
AT1G45248	AT1G36980	AT3G49940	AT5G64810
AT1G45249	AT1G36990	AT3G50260	AT5G65070
AT1G45332	AT1G41880	AT3G50300	AT5G65210
AT1G45976	AT1G42440	AT3G50480	AT5G65500
AT1G47230	AT1G42960	AT3G50560	AT5G65530
AT1G47260	AT1G42990	AT3G50630	AT5G65800
AT1G47278	AT1G43130	AT3G50640	AT5G65940
AT1G47330	AT1G43170	AT3G50820	AT5G66390
AT1G47380	AT1G43190	AT3G50840	AT5G66640
AT1G47420	AT1G43580	AT3G50950	AT5G66690
AT1G47550	AT1G43690	AT3G51895	AT5G67340
AT1G47570	AT1G43700	AT3G51910	AT5G67430
AT1G47580	AT1G43710	AT3G51950	
AT1G47640	AT1G43850	AT3G51960	
AT1G47670	AT1G43860	AT3G52060	
AT1G47710	AT1G43890	AT3G52490	
AT1G47740	AT1G43980	AT3G52500	
AT1G47830	AT1G44170	AT3G52700	
AT1G47900	AT1G44750	AT3G52840	
AT1G48030	AT1G44770	AT3G52910	
AT1G48040	AT1G44780	AT3G53150	
AT1G48050	AT1G44835	AT3G53180	
AT1G48090	AT1G44910	AT3G53200	
AT1G48110	AT1G44960	AT3G53210	
AT1G48120	AT1G45000	AT3G53260	
AT1G48140	AT1G45050	AT3G53420	
AT1G48160	AT1G45130	AT3G53960	
AT1G48175	AT1G45150	AT3G53990	
AT1G48195	AT1G45229	AT3G54140	
AT1G48200	AT1G45233	AT3G54320	
AT1G48210	AT1G45332	AT3G54740	
AT1G48230	AT1G45976	AT3G54770	
AT1G48270	AT1G47240	AT3G55180	
AT1G48310	AT1G47260	AT3G55230	
AT1G48360	AT1G47278	AT3G55515	
AT1G48380	AT1G47310	AT3G55610	
AT1G48410	AT1G47380	AT3G55700	
AT1G48430	AT1G47410	AT3G55740	
AT1G48440	AT1G47420	AT3G55840	
AT1G48635	AT1G47490	AT3G55910	
AT1G48650	AT1G47500	AT3G55950	
AT1G48760	AT1G47550	AT3G55970	
AT1G48780	AT1G47570	AT3G56080	
AT1G48790	AT1G47640	AT3G56220	
AT1G48830	AT1G47670	AT3G56950	
AT1G48840	AT1G47740	AT3G57010	
AT1G48850	AT1G47830	AT3G57020	
AT1G48860	AT1G47900	AT3G57330	
AT1G48890	AT1G48090	AT3G57460	
AT1G48900	AT1G48110	AT3G57520	
AT1G48920	AT1G48140	AT3G57630	
AT1G48950	AT1G48160	AT3G57770	
AT1G49040	AT1G48210	AT3G57920	
AT1G49180	AT1G48230	AT3G58350	
AT1G49300	AT1G48240	AT3G58720	
AT1G49340	AT1G48370	AT3G59020	
AT1G49350	AT1G48380	AT3G59120	
AT1G49360	AT1G48410	AT3G59130	
AT1G49405	AT1G48430	AT3G59220	
AT1G49410	AT1G48440	AT3G59260	

AT1G49480	AT1G48490	AT3G59370
AT1G49520	AT1G48550	AT3G59480
AT1G49530	AT1G48560	AT3G59710
AT1G49540	AT1G48570	AT3G59850
AT1G49600	AT1G48600	AT3G59900
AT1G49630	AT1G48610	AT3G60390
AT1G49670	AT1G48620	AT3G60470
AT1G49710	AT1G48635	AT3G60630
AT1G49720	AT1G48650	AT3G60720
AT1G49760	AT1G48790	AT3G61280
AT1G49820	AT1G48830	AT3G61660
AT1G49950	AT1G48840	AT3G61820
AT1G49970	AT1G48850	AT3G61850
AT1G50010	AT1G48900	AT3G61890
AT1G50030	AT1G48920	AT3G62070
AT1G50100	AT1G48930	AT3G62150
AT1G50120	AT1G48950	AT3G62270
AT1G50140	AT1G49040	AT3G62280
AT1G50200	AT1G49340	AT3G62570
AT1G50240	AT1G49350	AT3G62690
AT1G50260	AT1G49360	AT3G62700
AT1G50300	AT1G49480	AT3G62760
AT1G50360	AT1G49530	AT3G62930
AT1G50370	AT1G49540	AT3G62960
AT1G50380	AT1G49590	AT3G63050
AT1G50410	AT1G49600	AT4G00150
AT1G50430	AT1G49670	AT4G00460
AT1G50460	AT1G49710	AT4G00730
AT1G50480	AT1G49730	AT4G00780
AT1G50500	AT1G49760	AT4G00890
AT1G50520	AT1G49820	AT4G00950
AT1G50600	AT1G49890	AT4G01130
AT1G50660	AT1G49950	AT4G01140
AT1G50730	AT1G49960	AT4G01330
AT1G50750	AT1G49970	AT4G01630
AT1G50840	AT1G49980	AT4G02090
AT1G50910	AT1G50010	AT4G02670
AT1G50920	AT1G50020	AT4G02710
AT1G50940	AT1G50030	AT4G02850
AT1G50970	AT1G50120	AT4G03415
AT1G51160	AT1G50140	AT4G03420
AT1G51200	AT1G50190	AT4G03610
AT1G51310	AT1G50200	AT4G03630
AT1G51350	AT1G50260	AT4G03930
AT1G51400	AT1G50300	AT4G04490
AT1G51402	AT1G50360	AT4G04710
AT1G51510	AT1G50370	AT4G04970
AT1G51560	AT1G50380	AT4G05150
AT1G51570	AT1G50410	AT4G05220
AT1G51590	AT1G50430	AT4G05430
AT1G51600	AT1G50440	AT4G06744
AT1G51630	AT1G50460	AT4G06746
AT1G51650	AT1G50480	AT4G07670
AT1G51690	AT1G50500	AT4G07820
AT1G51710	AT1G50510	AT4G08290
AT1G51720	AT1G50560	AT4G09210
AT1G51730	AT1G50620	AT4G09950
AT1G51740	AT1G50660	AT4G10500
AT1G51745	AT1G50710	AT4G11190
AT1G51980	AT1G50730	AT4G11210
AT1G52150	AT1G50750	AT4G11290
AT1G52160	AT1G50840	AT4G11360
AT1G52310	AT1G51070	AT4G11820
AT1G52360	AT1G51130	AT4G11830
AT1G52370	AT1G51160	AT4G12430
AT1G52380	AT1G51200	AT4G12432
AT1G52420	AT1G51310	AT4G12510
AT1G52500	AT1G51350	AT4G12520
AT1G52520	AT1G51450	AT4G12730
AT1G52570	AT1G51540	AT4G13510
AT1G52580	AT1G51580	AT4G13580
AT1G52600	AT1G51590	AT4G13890
AT1G52630	AT1G51600	AT4G13992
AT1G52670	AT1G51630	AT4G14040
AT1G52730	AT1G51650	AT4G14610
AT1G52740	AT1G51690	AT4G14740
AT1G52820	AT1G51710	AT4G14990
AT1G52980	AT1G51720	AT4G15215
AT1G53000	AT1G51740	AT4G15230
AT1G53040	AT1G51745	AT4G15300
AT1G53050	AT1G51760	AT4G15370
AT1G53165	AT1G51980	AT4G15430
AT1G53190	AT1G52150	AT4G15480
AT1G53210	AT1G52260	AT4G15610
AT1G53240	AT1G52300	AT4G15660
AT1G53300	AT1G52320	AT4G15670
AT1G53310	AT1G52340	AT4G15680
AT1G53320	AT1G52360	AT4G15690
AT1G53350	AT1G52370	AT4G15990
AT1G53380	AT1G52380	AT4G16000
AT1G53390	AT1G52420	AT4G16008
AT1G53410	AT1G52600	AT4G16190
AT1G53490	AT1G52620	AT4G16480
AT1G53500	AT1G52630	AT4G16690
AT1G53510	AT1G52640	AT4G16860
AT1G53570	AT1G52670	AT4G16890
AT1G53590	AT1G52730	AT4G16920
AT1G53670	AT1G52740	AT4G16950
AT1G53680	AT1G52930	AT4G17070
AT1G53710	AT1G52980	AT4G18020
AT1G53720	AT1G52990	AT4G18340
AT1G53730	AT1G53040	AT4G18510
AT1G53750	AT1G53050	AT4G18550
AT1G53760	AT1G53165	AT4G18880
AT1G53780	AT1G53200	AT4G19030
AT1G53800	AT1G53210	AT4G19410
AT1G53801	AT1G53280	AT4G20070
AT1G53910	AT1G53300	AT4G20110
AT1G54070	AT1G53310	AT4G20140
AT1G54080	AT1G53320	AT4G20260
AT1G54100	AT1G53350	AT4G20270
AT1G54115	AT1G53380	AT4G20320
AT1G54140	AT1G53400	AT4G20420
AT1G54170	AT1G53420	AT4G20430
AT1G54220	AT1G53440	AT4G20830
AT1G54260	AT1G53450	AT4G20840
AT1G54270	AT1G53500	AT4G20860
AT1G54320	AT1G53510	AT4G21120
AT1G54340	AT1G53570	AT4G21310
AT1G54350	AT1G53590	AT4G21326
AT1G54355	AT1G53645	AT4G21340
AT1G54370	AT1G53720	AT4G22050
AT1G54385	AT1G53730	AT4G22110

AT1G54390	AT1G53770	AT4G22214
AT1G54440	AT1G53780	AT4G22230
AT1G54460	AT1G54080	AT4G22570
AT1G54490	AT1G54100	AT4G22590
AT1G54510	AT1G54130	AT4G22592
AT1G54570	AT1G54140	AT4G22666
AT1G54580	AT1G54150	AT4G22690
AT1G54610	AT1G54170	AT4G22710
AT1G54630	AT1G54180	AT4G22753
AT1G54650	AT1G54210	AT4G22980
AT1G54710	AT1G54270	AT4G23030
AT1G54850	AT1G54320	AT4G23100
AT1G54920	AT1G54350	AT4G23220
AT1G54990	AT1G54360	AT4G23260
AT1G55090	AT1G54370	AT4G23270
AT1G55130	AT1G54380	AT4G23280
AT1G55150	AT1G54390	AT4G23420
AT1G55175	AT1G54440	AT4G23590
AT1G55207	AT1G54460	AT4G23680
AT1G55250	AT1G54490	AT4G23690
AT1G55270	AT1G54510	AT4G23700
AT1G55310	AT1G54520	AT4G23900
AT1G55325	AT1G54580	AT4G24015
AT1G55340	AT1G54610	AT4G24050
AT1G55350	AT1G54710	AT4G24180
AT1G55490	AT1G54990	AT4G24250
AT1G55500	AT1G55040	AT4G24780
AT1G55530	AT1G55090	AT4G24890
AT1G55540	AT1G55130	AT4G25000
AT1G55590	AT1G55140	AT4G25050
AT1G55680	AT1G55150	AT4G25080
AT1G55690	AT1G55175	AT4G25170
AT1G55730	AT1G55205	AT4G25250
AT1G55750	AT1G55250	AT4G25400
AT1G55810	AT1G55310	AT4G25410
AT1G55820	AT1G55325	AT4G26200
AT1G55830	AT1G55340	AT4G26260
AT1G55840	AT1G55350	AT4G26320
AT1G55860	AT1G55490	AT4G26380
AT1G55880	AT1G55500	AT4G27730
AT1G55900	AT1G55520	AT4G27850
AT1G55970	AT1G55540	AT4G27860
AT1G56000	AT1G55620	AT4G27950
AT1G56050	AT1G55680	AT4G28085
AT1G56070	AT1G55690	AT4G28250
AT1G56090	AT1G55730	AT4G28290
AT1G56110	AT1G55820	AT4G28530
AT1G56140	AT1G55830	AT4G28650
AT1G56145	AT1G55840	AT4G28840
AT1G56170	AT1G55860	AT4G29210
AT1G56180	AT1G55870	AT4G29270
AT1G56280	AT1G55900	AT4G29305
AT1G56290	AT1G55930	AT4G29310
AT1G56310	AT1G55970	AT4G29380
AT1G56320	AT1G56070	AT4G29690
AT1G56330	AT1G56100	AT4G29700
AT1G56340	AT1G56110	AT4G29710
AT1G56423	AT1G56120	AT4G29740
AT1G56440	AT1G56130	AT4G29905
AT1G56450	AT1G56140	AT4G29920
AT1G56460	AT1G56145	AT4G30170
AT1G56470	AT1G56180	AT4G30190
AT1G56590	AT1G56200	AT4G30250
AT1G56610	AT1G56290	AT4G30270
AT1G56700	AT1G56340	AT4G30410
AT1G57600	AT1G56350	AT4G30520
AT1G57620	AT1G56418	AT4G31500
AT1G57680	AT1G56440	AT4G31610
AT1G57700	AT1G56450	AT4G31620
AT1G57720	AT1G56570	AT4G32540
AT1G57765	AT1G56590	AT4G32860
AT1G57820	AT1G56690	AT4G33010
AT1G57870	AT1G56700	AT4G33050
AT1G58025	AT1G57600	AT4G33300
AT1G58030	AT1G57620	AT4G33490
AT1G58060	AT1G57680	AT4G33580
AT1G58070	AT1G57700	AT4G34050
AT1G58080	AT1G57720	AT4G34138
AT1G58110	AT1G57765	AT4G34220
AT1G58200	AT1G57820	AT4G34230
AT1G58220	AT1G57870	AT4G34530
AT1G58230	AT1G58025	AT4G34560
AT1G58250	AT1G58030	AT4G34588
AT1G58280	AT1G58060	AT4G34590
AT1G58350	AT1G58070	AT4G34800
AT1G58400	AT1G58080	AT4G34810
AT1G58410	AT1G58200	AT4G35060
AT1G58440	AT1G58220	AT4G35110
AT1G58470	AT1G58230	AT4G35150
AT1G59520	AT1G58250	AT4G35270
AT1G59540	AT1G58280	AT4G35290
AT1G59560	AT1G58350	AT4G35480
AT1G59580	AT1G58410	AT4G35660
AT1G59610	AT1G58440	AT4G35690
AT1G59650	AT1G59610	AT4G35725
AT1G59660	AT1G59650	AT4G35750
AT1G59750	AT1G59660	AT4G35770
AT1G59760	AT1G59750	AT4G35780
AT1G59820	AT1G59760	AT4G35970
AT1G59830	AT1G59820	AT4G36040
AT1G59870	AT1G59830	AT4G36380
AT1G59890	AT1G59870	AT4G36410
AT1G59990	AT1G59890	AT4G36430
AT1G60070	AT1G59900	AT4G36640
AT1G60140	AT1G59960	AT4G36670
AT1G60170	AT1G59980	AT4G36880
AT1G60200	AT1G60070	AT4G36988
AT1G60220	AT1G60080	AT4G36990
AT1G60420	AT1G60140	AT4G37150
AT1G60430	AT1G60170	AT4G37250
AT1G60440	AT1G60200	AT4G37400
AT1G60490	AT1G60220	AT4G37445
AT1G60540	AT1G60230	AT4G37450
AT1G60545	AT1G60490	AT4G37520
AT1G60620	AT1G60620	AT4G37530
AT1G60640	AT1G60640	AT4G37540
AT1G60650	AT1G60650	AT4G37550
AT1G60670	AT1G60670	AT4G37610
AT1G60700	AT1G60680	AT4G37750
AT1G60770	AT1G60710	AT4G37790
AT1G60780	AT1G60780	AT4G37800
AT1G60800	AT1G60800	AT4G38470
AT1G60850	AT1G60850	AT4G39010

AT1G60860	AT1G60860	AT4G39030
AT1G60900	AT1G60900	AT4G39070
AT1G60940	AT1G60940	AT4G39260
AT1G60989	AT1G60995	AT4G39640
AT1G60990	AT1G61100	AT4G39650
AT1G60995	AT1G61140	AT4G39660
AT1G61000	AT1G61150	AT4G39675
AT1G61010	AT1G61210	AT4G39760
AT1G61030	AT1G61250	AT4G39770
AT1G61040	AT1G61360	AT4G39780
AT1G61100	AT1G61580	AT4G39810
AT1G61140	AT1G61620	AT4G39850
AT1G61150	AT1G61680	AT4G39940
AT1G61210	AT1G61770	AT4G39950
AT1G61215	AT1G61780	AT4G39970
AT1G61226	AT1G61850	AT4G40060
AT1G61240	AT1G62020	AT5G01370
AT1G61250	AT1G62040	AT5G01600
AT1G61310	AT1G62240	AT5G02020
AT1G61430	AT1G62260	AT5G02030
AT1G61490	AT1G62330	AT5G02140
AT1G61580	AT1G62390	AT5G02160
AT1G61620	AT1G62430	AT5G02180
AT1G61640	AT1G62600	AT5G02270
AT1G61690	AT1G62610	AT5G02580
AT1G61770	AT1G62740	AT5G02780
AT1G61780	AT1G62750	AT5G02840
AT1G61850	AT1G62780	AT5G02880
AT1G61860	AT1G62830	AT5G03240
AT1G61900	AT1G62880	AT5G03350
AT1G62020	AT1G63020	AT5G03510
AT1G62250	AT1G63160	AT5G03680
AT1G62310	AT1G63260	AT5G03700
AT1G62330	AT1G63290	AT5G03860
AT1G62355	AT1G63430	AT5G03960
AT1G62390	AT1G63480	AT5G04120
AT1G62430	AT1G63490	AT5G04330
AT1G62600	AT1G63500	AT5G04370
AT1G62610	AT1G63640	AT5G04470
AT1G62680	AT1G63660	AT5G04680
AT1G62730	AT1G63690	AT5G04690
AT1G62840	AT1G63700	AT5G04770
AT1G62850	AT1G63740	AT5G04890
AT1G62910	AT1G63750	AT5G04930
AT1G62970	AT1G63770	AT5G05340
AT1G63010	AT1G63780	AT5G05600
AT1G63020	AT1G63810	AT5G05890
AT1G63110	AT1G63830	AT5G05965
AT1G63150	AT1G63900	AT5G06730
AT1G63250	AT1G63940	AT5G06740
AT1G63270	AT1G63970	AT5G06790
AT1G63290	AT1G63980	AT5G06870
AT1G63430	AT1G64040	AT5G07330
AT1G63460	AT1G64090	AT5G07440
AT1G63490	AT1G64230	AT5G07550
AT1G63640	AT1G64280	AT5G07770
AT1G63670	AT1G64385	AT5G07830
AT1G63690	AT1G64520	AT5G08130
AT1G63700	AT1G64530	AT5G08240
AT1G63770	AT1G64550	AT5G08260
AT1G63810	AT1G64570	AT5G08350
AT1G63830	AT1G64650	AT5G08370
AT1G63900	AT1G64720	AT5G09460
AT1G63940	AT1G64750	AT5G09461
AT1G63980	AT1G64790	AT5G09462
AT1G64040	AT1G64850	AT5G09463
AT1G64050	AT1G64880	AT5G09490
AT1G64090	AT1G64980	AT5G10210
AT1G64140	AT1G64990	AT5G10280
AT1G64142	AT1G65010	AT5G10430
AT1G64230	AT1G65030	AT5G10970
AT1G64250	AT1G65032	AT5G10990
AT1G64330	AT1G65070	AT5G11100
AT1G64385	AT1G65080	AT5G11160
AT1G64440	AT1G65220	AT5G11320
AT1G64460	AT1G65260	AT5G11420
AT1G64520	AT1G65270	AT5G11990
AT1G64530	AT1G65280	AT5G12050
AT1G64550	AT1G65290	AT5G12270
AT1G64570	AT1G65370	AT5G12440
AT1G64572	AT1G65410	AT5G12940
AT1G64628	AT1G65430	AT5G12950
AT1G64630	AT1G65440	AT5G12960
AT1G64650	AT1G65560	AT5G13180
AT1G64790	AT1G65580	AT5G13330
AT1G64960	AT1G65650	AT5G13710
AT1G64980	AT1G65660	AT5G13740
AT1G64990	AT1G65700	AT5G14070
AT1G65010	AT1G65840	AT5G14340
AT1G65040	AT1G65920	AT5G14430
AT1G65220	AT1G65930	AT5G14510
AT1G65230	AT1G65950	AT5G14880
AT1G65260	AT1G65980	AT5G14890
AT1G65270	AT1G66070	AT5G15210
AT1G65280	AT1G66150	AT5G15310
AT1G65290	AT1G66240	AT5G15410
AT1G65380	AT1G66260	AT5G15580
AT1G65410	AT1G66340	AT5G15600
AT1G65430	AT1G66345	AT5G15948
AT1G65440	AT1G66410	AT5G15950
AT1G65540	AT1G66430	AT5G16010
AT1G65580	AT1G66510	AT5G16080
AT1G65650	AT1G66520	AT5G16110
AT1G65660	AT1G66530	AT5G16560
AT1G65700	AT1G66680	AT5G17260
AT1G65820	AT1G66880	AT5G17300
AT1G65840	AT1G66900	AT5G17330
AT1G65920	AT1G67060	AT5G17700
AT1G65930	AT1G67120	AT5G18640
AT1G65950	AT1G67140	AT5G18650
AT1G66070	AT1G67230	AT5G18670
AT1G66150	AT1G67250	AT5G18840
AT1G66250	AT1G67300	AT5G18970
AT1G66260	AT1G67310	AT5G19100
AT1G66330	AT1G67325	AT5G19110
AT1G66340	AT1G67350	AT5G19230
AT1G66410	AT1G67430	AT5G19240
AT1G66430	AT1G67490	AT5G19250
AT1G66510	AT1G67530	AT5G19520
AT1G66530	AT1G67560	AT5G19600
AT1G66580	AT1G67580	AT5G19730
AT1G66590	AT1G67630	AT5G19740
AT1G66670	AT1G67690	AT5G20250

AT1G66680	AT1G67730	AT5G20430
AT1G66730	AT1G67785	AT5G20670
AT1G66750	AT1G67890	AT5G20870
AT1G67120	AT1G67930	AT5G20970
AT1G67140	AT1G67940	AT5G21150
AT1G67170	AT1G67950	AT5G21170
AT1G67190	AT1G67960	AT5G21280
AT1G67230	AT1G68030	AT5G21482
AT1G67280	AT1G68060	AT5G21940
AT1G67300	AT1G68100	AT5G21950
AT1G67310	AT1G68140	AT5G22630
AT1G67320	AT1G68185	AT5G22740
AT1G67325	AT1G68310	AT5G22870
AT1G67350	AT1G68370	AT5G22920
AT1G67430	AT1G68560	AT5G22930
AT1G67490	AT1G68580	AT5G23000
AT1G67500	AT1G68590	AT5G23050
AT1G67530	AT1G68640	AT5G23270
AT1G67580	AT1G68720	AT5G23280
AT1G67660	AT1G68930	AT5G23350
AT1G67670	AT1G68990	AT5G23360
AT1G67680	AT1G69010	AT5G23370
AT1G67690	AT1G69030	AT5G23380
AT1G67720	AT1G69060	AT5G23730
AT1G67880	AT1G69070	AT5G23830
AT1G67890	AT1G69200	AT5G23840
AT1G67950	AT1G69220	AT5G23860
AT1G68000	AT1G69340	AT5G24110
AT1G68020	AT1G69350	AT5G24200
AT1G68060	AT1G69420	AT5G24290
AT1G68100	AT1G69485	AT5G24530
AT1G68140	AT1G69510	AT5G24540
AT1G68290	AT1G69520	AT5G24580
AT1G68370	AT1G69620	AT5G24760
AT1G68580	AT1G69640	AT5G24770
AT1G68650	AT1G69670	AT5G24780
AT1G68720	AT1G69740	AT5G25370
AT1G68725	AT1G69830	AT5G25490
AT1G68820	AT1G69850	AT5G25620
AT1G68862	AT1G69960	AT5G25770
AT1G68870	AT1G70060	AT5G25810
AT1G68872	AT1G70180	AT5G25890
AT1G68910	AT1G70280	AT5G26190
AT1G68930	AT1G70290	AT5G26260
AT1G68935	AT1G70300	AT5G26280
AT1G68990	AT1G70320	AT5G26286
AT1G69010	AT1G70350	AT5G26620
AT1G69020	AT1G70490	AT5G26673
AT1G69060	AT1G70570	AT5G27420
AT1G69070	AT1G70590	AT5G27730
AT1G69170	AT1G70620	AT5G27930
AT1G69220	AT1G70630	AT5G28050
AT1G69250	AT1G70730	AT5G28080
AT1G69280	AT1G70760	AT5G28640
AT1G69325	AT1G70770	AT5G28770
AT1G69330	AT1G70790	AT5G28910
AT1G69340	AT1G70900	AT5G28960
AT1G69350	AT1G71010	AT5G34780
AT1G69360	AT1G71020	AT5G35740
AT1G69390	AT1G71070	AT5G36180
AT1G69420	AT1G71080	AT5G37180
AT1G69510	AT1G71110	AT5G37600
AT1G69587	AT1G71120	AT5G37640
AT1G69588	AT1G71180	AT5G38020
AT1G69620	AT1G71220	AT5G38350
AT1G69640	AT1G71235	AT5G38710
AT1G69670	AT1G71240	AT5G38770
AT1G69740	AT1G71250	AT5G38970
AT1G69830	AT1G71270	AT5G39080
AT1G69950	AT1G71300	AT5G39471
AT1G69960	AT1G71310	AT5G39660
AT1G70060	AT1G71330	AT5G39760
AT1G70150	AT1G71350	AT5G39785
AT1G70180	AT1G71400	AT5G40240
AT1G70280	AT1G71410	AT5G40390
AT1G70290	AT1G71420	AT5G40630
AT1G70300	AT1G71460	AT5G40690
AT1G70320	AT1G71696	AT5G40730
AT1G70330	AT1G71710	AT5G40890
AT1G70490	AT1G71720	AT5G40960
AT1G70570	AT1G71810	AT5G40980
AT1G70580	AT1G71820	AT5G41590
AT1G70610	AT1G71830	AT5G41700
AT1G70620	AT1G71840	AT5G42030
AT1G70730	AT1G71860	AT5G42180
AT1G70740	AT1G71900	AT5G42235
AT1G70770	AT1G72040	AT5G42530
AT1G70790	AT1G72050	AT5G42590
AT1G70900	AT1G72090	AT5G43420
AT1G71010	AT1G72180	AT5G43470
AT1G71020	AT1G72250	AT5G43570
AT1G71070	AT1G72270	AT5G43590
AT1G71110	AT1G72320	AT5G43600
AT1G71220	AT1G72340	AT5G43910
AT1G71240	AT1G72390	AT5G44390
AT1G71270	AT1G72410	AT5G44400
AT1G71330	AT1G72440	AT5G44417
AT1G71350	AT1G72540	AT5G44440
AT1G71480	AT1G72550	AT5G45180
AT1G71696	AT1G72650	AT5G45200
AT1G71800	AT1G72700	AT5G45210
AT1G71820	AT1G72710	AT5G45310
AT1G71830	AT1G72720	AT5G45320
AT1G71860	AT1G72730	AT5G45500
AT1G71865	AT1G72770	AT5G45530
AT1G71900	AT1G72830	AT5G46050
AT1G72050	AT1G72870	AT5G46530
AT1G72090	AT1G72970	AT5G46590
AT1G72175	AT1G73066	AT5G47435
AT1G72320	AT1G73090	AT5G47560
AT1G72370	AT1G73170	AT5G47950
AT1G72390	AT1G73177	AT5G47980
AT1G72410	AT1G73180	AT5G47990
AT1G72420	AT1G73190	AT5G48000
AT1G72480	AT1G73200	AT5G48010
AT1G72540	AT1G73340	AT5G48060
AT1G72550	AT1G73350	AT5G48100
AT1G72560	AT1G73390	AT5G48110
AT1G72650	AT1G73430	AT5G48160
AT1G72660	AT1G73460	AT5G48400
AT1G72700	AT1G73470	AT5G48620
AT1G72710	AT1G73480	AT5G48690
AT1G72730	AT1G73570	AT5G49320

AT1G72740	AT1G73720	AT5G49360
AT1G72770	AT1G73840	AT5G49448
AT1G72830	AT1G73920	AT5G49450
AT1G72880	AT1G73950	AT5G49660
AT1G73060	AT1G73960	AT5G49665
AT1G73066	AT1G73970	AT5G49760
AT1G73080	AT1G73980	AT5G50290
AT1G73090	AT1G73990	AT5G50390
AT1G73100	AT1G74030	AT5G50560
AT1G73180	AT1G74040	AT5G50600
AT1G73200	AT1G74088	AT5G50610
AT1G73230	AT1G74160	AT5G50700
AT1G73310	AT1G74170	AT5G50710
AT1G73320	AT1G74210	AT5G50720
AT1G73350	AT1G74230	AT5G50920
AT1G73390	AT1G74240	AT5G51260
AT1G73430	AT1G74260	AT5G51470
AT1G73450	AT1G74270	AT5G51520
AT1G73460	AT1G74320	AT5G51780
AT1G73470	AT1G74330	AT5G51790
AT1G73650	AT1G74340	AT5G52250
AT1G73720	AT1G74420	AT5G52270
AT1G73730	AT1G74510	AT5G52330
AT1G73780	AT1G74550	AT5G52710
AT1G73840	AT1G74600	AT5G52760
AT1G73875	AT1G74630	AT5G52882
AT1G73920	AT1G74690	AT5G53010
AT1G73930	AT1G74740	AT5G53250
AT1G73950	AT1G74750	AT5G53460
AT1G73960	AT1G74770	AT5G53588
AT1G73980	AT1G74800	AT5G53590
AT1G73990	AT1G74900	AT5G53820
AT1G74030	AT1G74910	AT5G53980
AT1G74045	AT1G74920	AT5G53990
AT1G74050	AT1G74960	AT5G54160
AT1G74055	AT1G75010	AT5G54530
AT1G74060	AT1G75140	AT5G54585
AT1G74088	AT1G75180	AT5G54710
AT1G74160	AT1G75200	AT5G55050
AT1G74170	AT1G75220	AT5G55120
AT1G74180	AT1G75230	AT5G55620
AT1G74200	AT1G75310	AT5G56040
AT1G74210	AT1G75330	AT5G56320
AT1G74230	AT1G75340	AT5G56540
AT1G74260	AT1G75370	AT5G56544
AT1G74320	AT1G75380	AT5G56550
AT1G74330	AT1G75400	AT5G56720
AT1G74340	AT1G75510	AT5G56870
AT1G74350	AT1G75560	AT5G56890
AT1G74510	AT1G75630	AT5G56970
AT1G74540	AT1G75660	AT5G57130
AT1G74560	AT1G75670	AT5G57350
AT1G74590	AT1G75680	AT5G57620
AT1G74600	AT1G75850	AT5G57660
AT1G74640	AT1G75990	AT5G57785
AT1G74690	AT1G76010	AT5G58650
AT1G74700	AT1G76030	AT5G58784
AT1G74750	AT1G76120	AT5G58900
AT1G74790	AT1G76140	AT5G59020
AT1G74910	AT1G76150	AT5G59080
AT1G74920	AT1G76160	AT5G59090
AT1G74950	AT1G76200	AT5G59450
AT1G75010	AT1G76260	AT5G59590
AT1G75020	AT1G76270	AT5G59630
AT1G75120	AT1G76280	AT5G60710
AT1G75170	AT1G76300	AT5G60800
AT1G75210	AT1G76380	AT5G60890
AT1G75220	AT1G76390	AT5G61260
AT1G75295	AT1G76400	AT5G61270
AT1G75300	AT1G76490	AT5G61440
AT1G75310	AT1G76510	AT5G61480
AT1G75330	AT1G76550	AT5G61590
AT1G75340	AT1G76580	AT5G62070
AT1G75370	AT1G76630	AT5G62170
AT1G75380	AT1G76670	AT5G62630
AT1G75400	AT1G76810	AT5G62920
AT1G75560	AT1G76850	AT5G62940
AT1G75660	AT1G76860	AT5G62960
AT1G75670	AT1G76900	AT5G63160
AT1G75840	AT1G76950	AT5G63450
AT1G75850	AT1G76970	AT5G63600
AT1G75990	AT1G77000	AT5G63660
AT1G76010	AT1G77010	AT5G63800
AT1G76030	AT1G77030	AT5G64110
AT1G76140	AT1G77080	AT5G64120
AT1G76150	AT1G77140	AT5G64410
AT1G76170	AT1G77170	AT5G64570
AT1G76270	AT1G77180	AT5G64620
AT1G76280	AT1G77220	AT5G65140
AT1G76340	AT1G77230	AT5G65230
AT1G76350	AT1G77290	AT5G65380
AT1G76390	AT1G77300	AT5G65390
AT1G76400	AT1G77320	AT5G65530
AT1G76405	AT1G77350	AT5G65640
AT1G76440	AT1G77405	AT5G65690
AT1G76510	AT1G77440	AT5G65790
AT1G76550	AT1G77460	AT5G65800
AT1G76580	AT1G77510	AT5G66050
AT1G76630	AT1G77550	AT5G66052
AT1G76710	AT1G77590	AT5G66170
AT1G76810	AT1G77610	AT5G66390
AT1G76850	AT1G77620	AT5G66440
AT1G76878	AT1G77670	AT5G66560
AT1G76900	AT1G77680	AT5G67050
AT1G76950	AT1G77710	AT5G67430
AT1G76970	AT1G77770	AT5G67620
AT1G77030	AT1G77800	
AT1G77080	AT1G77840	
AT1G77130	AT1G77890	
AT1G77138	AT1G77940	
AT1G77140	AT1G78150	
AT1G77180	AT1G78240	
AT1G77220	AT1G78280	
AT1G77230	AT1G78300	
AT1G77240	AT1G78420	
AT1G77250	AT1G78510	
AT1G77300	AT1G78570	
AT1G77350	AT1G78580	
AT1G77360	AT1G78690	
AT1G77440	AT1G78770	
AT1G77460	AT1G78780	
AT1G77480	AT1G78800	
AT1G77510	AT1G78882	

AT1G77550	AT1G78895
AT1G77580	AT1G78900
AT1G77590	AT1G78910
AT1G77600	AT1G78915
AT1G77620	AT1G78920
AT1G77670	AT1G78950
AT1G77680	AT1G79000
AT1G77720	AT1G79010
AT1G77750	AT1G79020
AT1G77770	AT1G79030
AT1G77800	AT1G79090
AT1G77840	AT1G79150
AT1G77890	AT1G79190
AT1G78130	AT1G79200
AT1G78240	AT1G79210
AT1G78280	AT1G79230
AT1G78420	AT1G79280
AT1G78560	AT1G79340
AT1G78590	AT1G79350
AT1G78650	AT1G79380
AT1G78690	AT1G79400
AT1G78800	AT1G79440
AT1G78870	AT1G79460
AT1G78880	AT1G79490
AT1G78882	AT1G79500
AT1G78895	AT1G79510
AT1G78900	AT1G79530
AT1G78920	AT1G79550
AT1G78922	AT1G79560
AT1G78960	AT1G79570
AT1G79000	AT1G79590
AT1G79010	AT1G79610
AT1G79020	AT1G79650
AT1G79030	AT1G79690
AT1G79050	AT1G79720
AT1G79090	AT1G79730
AT1G79150	AT1G79740
AT1G79190	AT1G79750
AT1G79210	AT1G79780
AT1G79230	AT1G79820
AT1G79280	AT1G79830
AT1G79350	AT1G79870
AT1G79440	AT1G79920
AT1G79460	AT1G79930
AT1G79490	AT1G79940
AT1G79500	AT1G79950
AT1G79510	AT1G79990
AT1G79530	AT1G80000
AT1G79550	AT1G80030
AT1G79570	AT1G80040
AT1G79590	AT1G80070
AT1G79610	AT1G80230
AT1G79650	AT1G80260
AT1G79690	AT1G80350
AT1G79740	AT1G80370
AT1G79750	AT1G80410
AT1G79820	AT1G80480
AT1G79830	AT1G80490
AT1G79920	AT1G80530
AT1G79930	AT1G80560
AT1G79940	AT1G80610
AT1G79950	AT1G80660
AT1G79960	AT1G80670
AT1G79990	AT1G80680
AT1G80020	AT1G80690
AT1G80030	AT1G80710
AT1G80070	AT1G80750
AT1G80200	AT1G80790
AT1G80210	AT1G80900
AT1G80230	AT1G80930
AT1G80260	AT1G80950
AT1G80270	AT2G01060
AT1G80350	AT2G01100
AT1G80360	AT2G01120
AT1G80410	AT2G01130
AT1G80460	AT2G01140
AT1G80490	AT2G01220
AT1G80530	AT2G01250
AT1G80560	AT2G01270
AT1G80570	AT2G01275
AT1G80600	AT2G01390
AT1G80670	AT2G01440
AT1G80680	AT2G01450
AT1G80750	AT2G01460
AT1G80790	AT2G01470
AT1G80910	AT2G01480
AT2G01060	AT2G01490
AT2G01100	AT2G01510
AT2G01130	AT2G01600
AT2G01180	AT2G01690
AT2G01220	AT2G01720
AT2G01250	AT2G01750
AT2G01270	AT2G01910
AT2G01330	AT2G01970
AT2G01440	AT2G02050
AT2G01450	AT2G02160
AT2G01460	AT2G02470
AT2G01470	AT2G02480
AT2G01480	AT2G02510
AT2G01490	AT2G02560
AT2G01600	AT2G02570
AT2G01630	AT2G02720
AT2G01690	AT2G02740
AT2G01720	AT2G02760
AT2G01750	AT2G02790
AT2G01755	AT2G02800
AT2G01910	AT2G02860
AT2G01970	AT2G02870
AT2G01980	AT2G02955
AT2G02040	AT2G02960
AT2G02090	AT2G02980
AT2G02160	AT2G03070
AT2G02180	AT2G03150
AT2G02370	AT2G03240
AT2G02390	AT2G03390
AT2G02470	AT2G03510
AT2G02480	AT2G03880
AT2G02510	AT2G04030
AT2G02560	AT2G04390
AT2G02570	AT2G04520
AT2G02720	AT2G04700
AT2G02730	AT2G04740
AT2G02741	AT2G04940

AT2G02760	AT2G05120
AT2G02790	AT2G05210
AT2G02800	AT2G05590
AT2G02860	AT2G05710
AT2G02870	AT2G05830
AT2G02910	AT2G05840
AT2G02960	AT2G05990
AT2G03060	AT2G06010
AT2G03070	AT2G06025
AT2G03120	AT2G06050
AT2G03140	AT2G06210
AT2G03150	AT2G06520
AT2G03270	AT2G06990
AT2G03340	AT2G07050
AT2G03350	AT2G07360
AT2G03380	AT2G11000
AT2G03390	AT2G11910
AT2G03470	AT2G12400
AT2G03480	AT2G12550
AT2G03510	AT2G13100
AT2G03640	AT2G13370
AT2G03667	AT2G13440
AT2G03670	AT2G13540
AT2G03680	AT2G13560
AT2G03690	AT2G13650
AT2G03770	AT2G13690
AT2G03780	AT2G13790
AT2G03820	AT2G13840
AT2G04030	AT2G14120
AT2G04235	AT2G14170
AT2G04270	AT2G14255
AT2G04305	AT2G14260
AT2G04340	AT2G14285
AT2G04350	AT2G14530
AT2G04390	AT2G14680
AT2G04400	AT2G14690
AT2G04410	AT2G14720
AT2G04560	AT2G14740
AT2G04630	AT2G14835
AT2G04650	AT2G15240
AT2G04660	AT2G15270
AT2G04842	AT2G15290
AT2G04850	AT2G15300
AT2G04852	AT2G15430
AT2G04865	AT2G15570
AT2G04880	AT2G15690
AT2G04910	AT2G15730
AT2G04940	AT2G15790
AT2G05120	AT2G15860
AT2G05170	AT2G15900
AT2G05171	AT2G15910
AT2G05210	AT2G16405
AT2G05220	AT2G16485
AT2G05260	AT2G16500
AT2G05320	AT2G16570
AT2G05590	AT2G16640
AT2G05630	AT2G16710
AT2G05710	AT2G16860
AT2G05715	AT2G16900
AT2G05753	AT2G16910
AT2G05755	AT2G16920
AT2G05830	AT2G16940
AT2G05990	AT2G16950
AT2G06000	AT2G17190
AT2G06005	AT2G17200
AT2G06010	AT2G17210
AT2G06040	AT2G17250
AT2G06050	AT2G17360
AT2G06210	AT2G17380
AT2G06510	AT2G17390
AT2G06520	AT2G17410
AT2G06530	AT2G17420
AT2G06822	AT2G17440
AT2G06990	AT2G17480
AT2G07050	AT2G17510
AT2G07170	AT2G17580
AT2G07340	AT2G17700
AT2G07360	AT2G17760
AT2G07669	AT2G17790
AT2G07674	AT2G17800
AT2G07688	AT2G17840
AT2G07693	AT2G17930
AT2G07722	AT2G18020
AT2G07767	AT2G18040
AT2G07808	AT2G18110
AT2G07835	AT2G18220
AT2G10950	AT2G18230
AT2G11000	AT2G18330
AT2G11115	AT2G18465
AT2G11240	AT2G18510
AT2G11520	AT2G18730
AT2G11891	AT2G18740
AT2G11910	AT2G18750
AT2G12400	AT2G18790
AT2G12550	AT2G18840
AT2G13100	AT2G18876
AT2G13370	AT2G18900
AT2G13440	AT2G18915
AT2G13540	AT2G18960
AT2G13542	AT2G18990
AT2G13560	AT2G19080
AT2G13650	AT2G19160
AT2G13790	AT2G19170
AT2G13800	AT2G19190
AT2G13840	AT2G19260
AT2G13970	AT2G19270
AT2G14045	AT2G19390
AT2G14120	AT2G19440
AT2G14170	AT2G19470
AT2G14255	AT2G19480
AT2G14260	AT2G19520
AT2G14285	AT2G19540
AT2G14530	AT2G19550
AT2G14680	AT2G19560
AT2G14720	AT2G19600
AT2G14740	AT2G19610
AT2G14835	AT2G19620
AT2G14850	AT2G19730
AT2G14910	AT2G19740
AT2G15230	AT2G19750
AT2G15240	AT2G19760
AT2G15530	AT2G19790
AT2G15570	AT2G19880

AT2G15695	AT2G19950
AT2G15730	AT2G20000
AT2G15790	AT2G20020
AT2G15860	AT2G20050
AT2G15880	AT2G20060
AT2G15900	AT2G20120
AT2G16090	AT2G20140
AT2G16245	AT2G20190
AT2G16250	AT2G20230
AT2G16370	AT2G20280
AT2G16390	AT2G20290
AT2G16405	AT2G20300
AT2G16485	AT2G20320
AT2G16510	AT2G20330
AT2G16640	AT2G20360
AT2G16650	AT2G20420
AT2G16710	AT2G20450
AT2G16800	AT2G20580
AT2G16860	AT2G20585
AT2G16900	AT2G20760
AT2G16910	AT2G20770
AT2G16920	AT2G20830
AT2G16940	AT2G20840
AT2G16950	AT2G20850
AT2G17020	AT2G20990
AT2G17033	AT2G21090
AT2G17036	AT2G21120
AT2G17150	AT2G21150
AT2G17190	AT2G21160
AT2G17200	AT2G21170
AT2G17250	AT2G21190
AT2G17260	AT2G21230
AT2G17340	AT2G21240
AT2G17360	AT2G21270
AT2G17380	AT2G21300
AT2G17390	AT2G21340
AT2G17410	AT2G21380
AT2G17480	AT2G21390
AT2G17510	AT2G21410
AT2G17520	AT2G21430
AT2G17530	AT2G21440
AT2G17570	AT2G21470
AT2G17580	AT2G21500
AT2G17700	AT2G21520
AT2G17760	AT2G21560
AT2G17790	AT2G21580
AT2G17800	AT2G21600
AT2G17930	AT2G21630
AT2G17970	AT2G21790
AT2G17975	AT2G21940
AT2G17980	AT2G22010
AT2G17990	AT2G22070
AT2G18020	AT2G22080
AT2G18040	AT2G22088
AT2G18090	AT2G22120
AT2G18100	AT2G22125
AT2G18110	AT2G22230
AT2G18170	AT2G22300
AT2G18220	AT2G22310
AT2G18230	AT2G22360
AT2G18330	AT2G22400
AT2G18400	AT2G22475
AT2G18465	AT2G22490
AT2G18510	AT2G22530
AT2G18530	AT2G22720
AT2G18710	AT2G22840
AT2G18730	AT2G23070
AT2G18740	AT2G23080
AT2G18750	AT2G23140
AT2G18760	AT2G23310
AT2G18790	AT2G23321
AT2G18850	AT2G23350
AT2G18876	AT2G23370
AT2G18900	AT2G23380
AT2G18915	AT2G23420
AT2G18960	AT2G23460
AT2G19045	AT2G23820
AT2G19080	AT2G23890
AT2G19090	AT2G23930
AT2G19120	AT2G23945
AT2G19160	AT2G23980
AT2G19170	AT2G24050
AT2G19250	AT2G24120
AT2G19260	AT2G24200
AT2G19330	AT2G24320
AT2G19385	AT2G24330
AT2G19390	AT2G24420
AT2G19470	AT2G24430
AT2G19480	AT2G24570
AT2G19490	AT2G24640
AT2G19520	AT2G24690
AT2G19540	AT2G24765
AT2G19550	AT2G24970
AT2G19560	AT2G24990
AT2G19600	AT2G25070
AT2G19680	AT2G25080
AT2G19710	AT2G25110
AT2G19730	AT2G25140
AT2G19755	AT2G25170
AT2G19790	AT2G25180
AT2G19830	AT2G25280
AT2G19860	AT2G25320
AT2G19880	AT2G25355
AT2G19950	AT2G25420
AT2G20000	AT2G25430
AT2G20050	AT2G25460
AT2G20060	AT2G25560
AT2G20120	AT2G25570
AT2G20130	AT2G25620
AT2G20190	AT2G25640
AT2G20240	AT2G25660
AT2G20280	AT2G25670
AT2G20290	AT2G25730
AT2G20300	AT2G25740
AT2G20320	AT2G25760
AT2G20330	AT2G25800
AT2G20360	AT2G25840
AT2G20410	AT2G25870
AT2G20420	AT2G25910
AT2G20440	AT2G25930
AT2G20495	AT2G25970
AT2G20500	AT2G26000
AT2G20580	AT2G26060

AT2G20650	AT2G26070
AT2G20760	AT2G26080
AT2G20790	AT2G26100
AT2G20815	AT2G26135
AT2G20840	AT2G26140
AT2G20990	AT2G26240
AT2G21070	AT2G26260
AT2G21080	AT2G26270
AT2G21090	AT2G26280
AT2G21120	AT2G26300
AT2G21150	AT2G26310
AT2G21160	AT2G26430
AT2G21170	AT2G26460
AT2G21190	AT2G26470
AT2G21240	AT2G26510
AT2G21250	AT2G26590
AT2G21270	AT2G26610
AT2G21280	AT2G26650
AT2G21300	AT2G26660
AT2G21370	AT2G26770
AT2G21380	AT2G26780
AT2G21385	AT2G26800
AT2G21390	AT2G26810
AT2G21410	AT2G26830
AT2G21430	AT2G26840
AT2G21440	AT2G26890
AT2G21470	AT2G26900
AT2G21500	AT2G26920
AT2G21520	AT2G26990
AT2G21600	AT2G27020
AT2G21790	AT2G27040
AT2G21870	AT2G27100
AT2G21940	AT2G27110
AT2G22010	AT2G27170
AT2G22070	AT2G27200
AT2G22088	AT2G27210
AT2G22120	AT2G27285
AT2G22125	AT2G27350
AT2G22250	AT2G27460
AT2G22270	AT2G27470
AT2G22300	AT2G27510
AT2G22310	AT2G27530
AT2G22360	AT2G27610
AT2G22400	AT2G27660
AT2G22450	AT2G27720
AT2G22475	AT2G27810
AT2G22480	AT2G27860
AT2G22490	AT2G27900
AT2G22530	AT2G27950
AT2G22560	AT2G27980
AT2G22630	AT2G28000
AT2G22660	AT2G28050
AT2G22670	AT2G28070
AT2G22690	AT2G28220
AT2G22720	AT2G28240
AT2G22740	AT2G28290
AT2G22780	AT2G28310
AT2G22795	AT2G28320
AT2G22840	AT2G28360
AT2G22880	AT2G28370
AT2G22910	AT2G28380
AT2G23070	AT2G28390
AT2G23080	AT2G28450
AT2G23093	AT2G28520
AT2G23140	AT2G28540
AT2G23350	AT2G28600
AT2G23380	AT2G28910
AT2G23420	AT2G29020
AT2G23460	AT2G29070
AT2G23520	AT2G29080
AT2G23550	AT2G29140
AT2G23560	AT2G29180
AT2G23700	AT2G29190
AT2G23740	AT2G29200
AT2G23780	AT2G29210
AT2G23820	AT2G29260
AT2G23890	AT2G29390
AT2G23945	AT2G29530
AT2G23980	AT2G29540
AT2G24020	AT2G29550
AT2G24050	AT2G29560
AT2G24200	AT2G29570
AT2G24300	AT2G29580
AT2G24360	AT2G29640
AT2G24420	AT2G29690
AT2G24520	AT2G29760
AT2G24530	AT2G30000
AT2G24590	AT2G30020
AT2G24592	AT2G30050
AT2G24640	AT2G30060
AT2G24650	AT2G30070
AT2G24690	AT2G30105
AT2G24765	AT2G30110
AT2G24830	AT2G30200
AT2G24960	AT2G30260
AT2G24990	AT2G30280
AT2G25010	AT2G30440
AT2G25050	AT2G30460
AT2G25070	AT2G30470
AT2G25100	AT2G30575
AT2G25140	AT2G30600
AT2G25170	AT2G30700
AT2G25171	AT2G30800
AT2G25180	AT2G30910
AT2G25270	AT2G30920
AT2G25280	AT2G30950
AT2G25290	AT2G30970
AT2G25310	AT2G30980
AT2G25320	AT2G31010
AT2G25350	AT2G31060
AT2G25355	AT2G31140
AT2G25420	AT2G31260
AT2G25430	AT2G31280
AT2G25480	AT2G31300
AT2G25560	AT2G31320
AT2G25620	AT2G31370
AT2G25640	AT2G31380
AT2G25660	AT2G31390
AT2G25670	AT2G31400
AT2G25710	AT2G31440
AT2G25730	AT2G31490
AT2G25740	AT2G31510
AT2G25760	AT2G31530

AT2G25800	AT2G31610
AT2G25850	AT2G31650
AT2G25910	AT2G31660
AT2G25920	AT2G31740
AT2G25930	AT2G31810
AT2G25950	AT2G31820
AT2G25970	AT2G31830
AT2G26000	AT2G31955
AT2G26060	AT2G31960
AT2G26080	AT2G31970
AT2G26100	AT2G31980
AT2G26140	AT2G32000
AT2G26200	AT2G32040
AT2G26210	AT2G32050
AT2G26230	AT2G32060
AT2G26240	AT2G32170
AT2G26260	AT2G32235
AT2G26270	AT2G32250
AT2G26280	AT2G32260
AT2G26300	AT2G32290
AT2G26310	AT2G32300
AT2G26430	AT2G32320
AT2G26460	AT2G32410
AT2G26510	AT2G32520
AT2G26540	AT2G32580
AT2G26570	AT2G32600
AT2G26590	AT2G32630
AT2G26660	AT2G32700
AT2G26770	AT2G32710
AT2G26780	AT2G32720
AT2G26800	AT2G32730
AT2G26810	AT2G32760
AT2G26830	AT2G32810
AT2G26890	AT2G32850
AT2G26900	AT2G32910
AT2G26920	AT2G32920
AT2G26930	AT2G32930
AT2G26970	AT2G32950
AT2G26980	AT2G32970
AT2G26990	AT2G32980
AT2G27020	AT2G33050
AT2G27040	AT2G33120
AT2G27060	AT2G33150
AT2G27090	AT2G33170
AT2G27100	AT2G33210
AT2G27110	AT2G33240
AT2G27170	AT2G33340
AT2G27190	AT2G33370
AT2G27210	AT2G33430
AT2G27228	AT2G33435
AT2G27230	AT2G33440
AT2G27350	AT2G33470
AT2G27450	AT2G33490
AT2G27460	AT2G33500
AT2G27490	AT2G33590
AT2G27510	AT2G33610
AT2G27530	AT2G33620
AT2G27610	AT2G33630
AT2G27710	AT2G33700
AT2G27730	AT2G33720
AT2G27760	AT2G33740
AT2G27810	AT2G33750
AT2G27840	AT2G33760
AT2G27860	AT2G33770
AT2G27900	AT2G33800
AT2G27950	AT2G33840
AT2G27980	AT2G33980
AT2G28000	AT2G34040
AT2G28040	AT2G34090
AT2G28060	AT2G34180
AT2G28070	AT2G34250
AT2G28130	AT2G34260
AT2G28150	AT2G34300
AT2G28240	AT2G34357
AT2G28290	AT2G34400
AT2G28310	AT2G34410
AT2G28320	AT2G34450
AT2G28360	AT2G34480
AT2G28370	AT2G34560
AT2G28380	AT2G34570
AT2G28390	AT2G34590
AT2G28426	AT2G34630
AT2G28440	AT2G34660
AT2G28450	AT2G34670
AT2G28480	AT2G34680
AT2G28520	AT2G34710
AT2G28540	AT2G34730
AT2G28600	AT2G34750
AT2G28620	AT2G34770
AT2G28670	AT2G34780
AT2G28671	AT2G34840
AT2G28800	AT2G34860
AT2G28880	AT2G34900
AT2G28910	AT2G34970
AT2G29020	AT2G35030
AT2G29080	AT2G35040
AT2G29140	AT2G35050
AT2G29190	AT2G35060
AT2G29200	AT2G35080
AT2G29210	AT2G35110
AT2G29263	AT2G35120
AT2G29390	AT2G35190
AT2G29400	AT2G35240
AT2G29510	AT2G35320
AT2G29560	AT2G35330
AT2G29580	AT2G35340
AT2G29760	AT2G35350
AT2G29970	AT2G35510
AT2G29990	AT2G35520
AT2G30050	AT2G35530
AT2G30060	AT2G35605
AT2G30070	AT2G35610
AT2G30110	AT2G35615
AT2G30200	AT2G35620
AT2G30260	AT2G35630
AT2G30280	AT2G35635
AT2G30410	AT2G35650
AT2G30470	AT2G35790
AT2G30575	AT2G35800
AT2G30580	AT2G35920
AT2G30600	AT2G36060
AT2G30615	AT2G36070
AT2G30640	AT2G36130

AT2G30690	AT2G36160
AT2G30700	AT2G36170
AT2G30710	AT2G36200
AT2G30740	AT2G36240
AT2G30780	AT2G36250
AT2G30800	AT2G36290
AT2G30880	AT2G36330
AT2G30910	AT2G36350
AT2G30920	AT2G36355
AT2G30950	AT2G36360
AT2G30970	AT2G36380
AT2G30980	AT2G36390
AT2G31010	AT2G36400
AT2G31060	AT2G36480
AT2G31140	AT2G36530
AT2G31170	AT2G36571
AT2G31190	AT2G36580
AT2G31260	AT2G36620
AT2G31280	AT2G36670
AT2G31300	AT2G36680
AT2G31305	AT2G36720
AT2G31320	AT2G36740
AT2G31350	AT2G36810
AT2G31450	AT2G36835
AT2G31510	AT2G36850
AT2G31530	AT2G36880
AT2G31580	AT2G36900
AT2G31610	AT2G36910
AT2G31660	AT2G36930
AT2G31740	AT2G36960
AT2G31820	AT2G37020
AT2G31830	AT2G37050
AT2G31900	AT2G37080
AT2G31902	AT2G37110
AT2G31955	AT2G37160
AT2G31960	AT2G37200
AT2G31970	AT2G37250
AT2G32000	AT2G37260
AT2G32040	AT2G37270
AT2G32060	AT2G37280
AT2G32170	AT2G37300
AT2G32230	AT2G37340
AT2G32240	AT2G37510
AT2G32250	AT2G37520
AT2G32260	AT2G37550
AT2G32295	AT2G37560
AT2G32320	AT2G37660
AT2G32340	AT2G37680
AT2G32400	AT2G37690
AT2G32410	AT2G37700
AT2G32520	AT2G37790
AT2G32600	AT2G37840
AT2G32700	AT2G37890
AT2G32710	AT2G37940
AT2G32720	AT2G37975
AT2G32730	AT2G37990
AT2G32760	AT2G38020
AT2G32810	AT2G38040
AT2G32850	AT2G38130
AT2G32900	AT2G38195
AT2G32910	AT2G38280
AT2G32920	AT2G38290
AT2G32930	AT2G38370
AT2G32950	AT2G38410
AT2G32970	AT2G38440
AT2G32980	AT2G38550
AT2G33040	AT2G38560
AT2G33050	AT2G38570
AT2G33051	AT2G38580
AT2G33120	AT2G38610
AT2G33150	AT2G38650
AT2G33170	AT2G38670
AT2G33210	AT2G38700
AT2G33240	AT2G38710
AT2G33255	AT2G38770
AT2G33340	AT2G38800
AT2G33360	AT2G38810
AT2G33370	AT2G38840
AT2G33430	AT2G38880
AT2G33435	AT2G38950
AT2G33440	AT2G39080
AT2G33470	AT2G39090
AT2G33490	AT2G39130
AT2G33540	AT2G39170
AT2G33590	AT2G39190
AT2G33610	AT2G39260
AT2G33630	AT2G39270
AT2G33680	AT2G39280
AT2G33700	AT2G39340
AT2G33770	AT2G39350
AT2G33810	AT2G39390
AT2G33815	AT2G39470
AT2G33820	AT2G39480
AT2G33835	AT2G39580
AT2G33840	AT2G39620
AT2G33900	AT2G39630
AT2G33920	AT2G39740
AT2G33930	AT2G39750
AT2G33940	AT2G39770
AT2G33950	AT2G39780
AT2G33960	AT2G39800
AT2G33970	AT2G39805
AT2G33980	AT2G39810
AT2G33990	AT2G39900
AT2G34040	AT2G39930
AT2G34090	AT2G39940
AT2G34250	AT2G39950
AT2G34300	AT2G39960
AT2G34357	AT2G39970
AT2G34410	AT2G39990
AT2G34440	AT2G40095
AT2G34450	AT2G40190
AT2G34480	AT2G40270
AT2G34560	AT2G40316
AT2G34590	AT2G40320
AT2G34630	AT2G40360
AT2G34640	AT2G40430
AT2G34660	AT2G40510
AT2G34670	AT2G40540
AT2G34680	AT2G40550
AT2G34710	AT2G40590
AT2G34720	AT2G40600
AT2G34730	AT2G40620

AT2G34750	AT2G40630
AT2G34770	AT2G40640
AT2G34780	AT2G40650
AT2G34850	AT2G40660
AT2G34900	AT2G40690
AT2G34930	AT2G40700
AT2G34970	AT2G40720
AT2G35035	AT2G40730
AT2G35040	AT2G40765
AT2G35050	AT2G40770
AT2G35060	AT2G40790
AT2G35110	AT2G40810
AT2G35120	AT2G40815
AT2G35155	AT2G40820
AT2G35160	AT2G40840
AT2G35180	AT2G40930
AT2G35230	AT2G40935
AT2G35320	AT2G40940
AT2G35330	AT2G40950
AT2G35340	AT2G41000
AT2G35420	AT2G41020
AT2G35500	AT2G41080
AT2G35510	AT2G41140
AT2G35530	AT2G41150
AT2G35540	AT2G41160
AT2G35610	AT2G41220
AT2G35620	AT2G41250
AT2G35630	AT2G41310
AT2G35650	AT2G41350
AT2G35680	AT2G41420
AT2G35738	AT2G41500
AT2G35740	AT2G41520
AT2G35790	AT2G41530
AT2G35795	AT2G41620
AT2G35880	AT2G41630
AT2G35920	AT2G41700
AT2G36060	AT2G41710
AT2G36070	AT2G41740
AT2G36130	AT2G41760
AT2G36160	AT2G41790
AT2G36250	AT2G41840
AT2G36290	AT2G41890
AT2G36305	AT2G41900
AT2G36310	AT2G41945
AT2G36350	AT2G41950
AT2G36355	AT2G41960
AT2G36360	AT2G42010
AT2G36370	AT2G42040
AT2G36390	AT2G42120
AT2G36480	AT2G42160
AT2G36530	AT2G42190
AT2G36580	AT2G42230
AT2G36610	AT2G42270
AT2G36620	AT2G42280
AT2G36670	AT2G42330
AT2G36680	AT2G42390
AT2G36720	AT2G42400
AT2G36740	AT2G42490
AT2G36810	AT2G42500
AT2G36850	AT2G42520
AT2G36880	AT2G42570
AT2G36910	AT2G42580
AT2G36930	AT2G42600
AT2G36960	AT2G42640
AT2G37020	AT2G42680
AT2G37050	AT2G42710
AT2G37150	AT2G42780
AT2G37160	AT2G42790
AT2G37230	AT2G42810
AT2G37250	AT2G42880
AT2G37270	AT2G42910
AT2G37340	AT2G42920
AT2G37500	AT2G43018
AT2G37520	AT2G43040
AT2G37570	AT2G43070
AT2G37690	AT2G43080
AT2G37840	AT2G43110
AT2G37860	AT2G43160
AT2G37890	AT2G43200
AT2G37940	AT2G43210
AT2G37975	AT2G43230
AT2G37990	AT2G43235
AT2G38000	AT2G43240
AT2G38020	AT2G43320
AT2G38040	AT2G43360
AT2G38130	AT2G43410
AT2G38195	AT2G43420
AT2G38260	AT2G43430
AT2G38280	AT2G43460
AT2G38340	AT2G43480
AT2G38410	AT2G43490
AT2G38440	AT2G43630
AT2G38560	AT2G43640
AT2G38580	AT2G43650
AT2G38610	AT2G43710
AT2G38650	AT2G43720
AT2G38660	AT2G43750
AT2G38700	AT2G43760
AT2G38710	AT2G43770
AT2G38770	AT2G43780
AT2G38840	AT2G43790
AT2G38950	AT2G43810
AT2G38960	AT2G43840
AT2G38970	AT2G43940
AT2G39080	AT2G43945
AT2G39090	AT2G43950
AT2G39170	AT2G43970
AT2G39190	AT2G43990
AT2G39260	AT2G44040
AT2G39280	AT2G44060
AT2G39300	AT2G44065
AT2G39340	AT2G44100
AT2G39380	AT2G44120
AT2G39390	AT2G44140
AT2G39440	AT2G44160
AT2G39445	AT2G44180
AT2G39450	AT2G44200
AT2G39470	AT2G44270
AT2G39480	AT2G44350
AT2G39550	AT2G44520
AT2G39580	AT2G44530
AT2G39630	AT2G44610
AT2G39740	AT2G44660

AT2G39770	AT2G44710
AT2G39780	AT2G44770
AT2G39805	AT2G44820
AT2G39810	AT2G44850
AT2G39840	AT2G44860
AT2G39930	AT2G44880
AT2G39940	AT2G44900
AT2G39950	AT2G44950
AT2G39960	AT2G44970
AT2G39970	AT2G45000
AT2G39990	AT2G45010
AT2G40008	AT2G45030
AT2G40030	AT2G45070
AT2G40060	AT2G45140
AT2G40070	AT2G45240
AT2G40090	AT2G45270
AT2G40160	AT2G45460
AT2G40190	AT2G45530
AT2G40270	AT2G45540
AT2G40280	AT2G45640
AT2G40360	AT2G45695
AT2G40430	AT2G45700
AT2G40490	AT2G45730
AT2G40540	AT2G45790
AT2G40580	AT2G45800
AT2G40590	AT2G45810
AT2G40600	AT2G45820
AT2G40630	AT2G45920
AT2G40650	AT2G45960
AT2G40660	AT2G46020
AT2G40690	AT2G46030
AT2G40700	AT2G46060
AT2G40730	AT2G46080
AT2G40770	AT2G46090
AT2G40790	AT2G46170
AT2G40800	AT2G46180
AT2G40820	AT2G46225
AT2G40830	AT2G46230
AT2G40840	AT2G46260
AT2G40850	AT2G46340
AT2G40930	AT2G46470
AT2G40940	AT2G46500
AT2G40980	AT2G46520
AT2G41020	AT2G46540
AT2G41140	AT2G46560
AT2G41150	AT2G46700
AT2G41160	AT2G46780
AT2G41220	AT2G46800
AT2G41250	AT2G46810
AT2G41490	AT2G46900
AT2G41500	AT2G46910
AT2G41520	AT2G46915
AT2G41530	AT2G46920
AT2G41560	AT2G47000
AT2G41620	AT2G47060
AT2G41700	AT2G47070
AT2G41710	AT2G47115
AT2G41740	AT2G47170
AT2G41790	AT2G47210
AT2G41835	AT2G47230
AT2G41890	AT2G47250
AT2G41900	AT2G47320
AT2G41945	AT2G47350
AT2G41960	AT2G47390
AT2G41980	AT2G47410
AT2G42010	AT2G47430
AT2G42030	AT2G47470
AT2G42080	AT2G47510
AT2G42120	AT2G47580
AT2G42210	AT2G47590
AT2G42230	AT2G47600
AT2G42240	AT2G47610
AT2G42247	AT2G47640
AT2G42250	AT2G47650
AT2G42270	AT2G47680
AT2G42360	AT2G47700
AT2G42400	AT2G47830
AT2G42450	AT2G47900
AT2G42490	AT2G47940
AT2G42500	AT2G47960
AT2G42520	AT2G47980
AT2G42590	AT2G47990
AT2G42600	AT2G48060
AT2G42640	AT2G48080
AT2G42670	AT2G48100
AT2G42680	AT2G48110
AT2G42700	AT2G48121
AT2G42750	AT2G48160
AT2G42780	AT3G01040
AT2G42790	AT3G01050
AT2G42810	AT3G01090
AT2G42880	AT3G01120
AT2G42890	AT3G01150
AT2G42910	AT3G01160
AT2G42930	AT3G01180
AT2G42940	AT3G01210
AT2G42960	AT3G01280
AT2G42980	AT3G01300
AT2G43018	AT3G01310
AT2G43020	AT3G01320
AT2G43040	AT3G01330
AT2G43070	AT3G01350
AT2G43080	AT3G01370
AT2G43100	AT3G01380
AT2G43110	AT3G01390
AT2G43137	AT3G01435
AT2G43138	AT3G01460
AT2G43139	AT3G01520
AT2G43141	AT3G01540
AT2G43160	AT3G01590
AT2G43190	AT3G01610
AT2G43210	AT3G01620
AT2G43240	AT3G01650
AT2G43250	AT3G01690
AT2G43320	AT3G01720
AT2G43330	AT3G01780
AT2G43360	AT3G01810
AT2G43370	AT3G01820
AT2G43410	AT3G01910
AT2G43420	AT3G02010
AT2G43430	AT3G02050
AT2G43490	AT3G02060
AT2G43500	AT3G02090

AT2G43630	AT3G02130
AT2G43640	AT3G02150
AT2G43650	AT3G02210
AT2G43680	AT3G02220
AT2G43710	AT3G02230
AT2G43750	AT3G02260
AT2G43760	AT3G02280
AT2G43770	AT3G02300
AT2G43790	AT3G02320
AT2G43810	AT3G02360
AT2G43950	AT3G02420
AT2G43980	AT3G02470
AT2G44050	AT3G02520
AT2G44060	AT3G02530
AT2G44065	AT3G02540
AT2G44090	AT3G02555
AT2G44100	AT3G02580
AT2G44120	AT3G02600
AT2G44180	AT3G02630
AT2G44200	AT3G02650
AT2G44270	AT3G02660
AT2G44350	AT3G02690
AT2G44420	AT3G02700
AT2G44440	AT3G02710
AT2G44510	AT3G02750
AT2G44520	AT3G02760
AT2G44530	AT3G02780
AT2G44610	AT3G02870
AT2G44640	AT3G02890
AT2G44710	AT3G02900
AT2G44800	AT3G02930
AT2G44820	AT3G02980
AT2G44830	AT3G03050
AT2G44850	AT3G03060
AT2G44860	AT3G03070
AT2G44900	AT3G03100
AT2G44950	AT3G03110
AT2G44970	AT3G03120
AT2G45010	AT3G03250
AT2G45030	AT3G03300
AT2G45070	AT3G03310
AT2G45100	AT3G03340
AT2G45150	AT3G03360
AT2G45200	AT3G03380
AT2G45240	AT3G03510
AT2G45270	AT3G03560
AT2G45320	AT3G03570
AT2G45380	AT3G03580
AT2G45440	AT3G03590
AT2G45460	AT3G03620
AT2G45500	AT3G03710
AT2G45540	AT3G03720
AT2G45640	AT3G03740
AT2G45670	AT3G03750
AT2G45690	AT3G03773
AT2G45700	AT3G03790
AT2G45880	AT3G03800
AT2G45910	AT3G03810
AT2G45920	AT3G03860
AT2G45950	AT3G03890
AT2G45980	AT3G03900
AT2G45990	AT3G03910
AT2G46020	AT3G03920
AT2G46070	AT3G03940
AT2G46080	AT3G03950
AT2G46090	AT3G03960
AT2G46170	AT3G04080
AT2G46180	AT3G04090
AT2G46200	AT3G04120
AT2G46225	AT3G04240
AT2G46240	AT3G04260
AT2G46260	AT3G04300
AT2G46280	AT3G04310
AT2G46320	AT3G04340
AT2G46340	AT3G04350
AT2G46470	AT3G04400
AT2G46500	AT3G04460
AT2G46520	AT3G04480
AT2G46540	AT3G04490
AT2G46550	AT3G04500
AT2G46560	AT3G04520
AT2G46567	AT3G04560
AT2G46570	AT3G04590
AT2G46572	AT3G04600
AT2G46610	AT3G04610
AT2G46700	AT3G04620
AT2G46800	AT3G04670
AT2G46900	AT3G04680
AT2G46910	AT3G04710
AT2G46920	AT3G04740
AT2G46930	AT3G04770
AT2G47000	AT3G04780
AT2G47070	AT3G04810
AT2G47090	AT3G04820
AT2G47170	AT3G04830
AT2G47210	AT3G04840
AT2G47220	AT3G04850
AT2G47320	AT3G04870
AT2G47350	AT3G04910
AT2G47380	AT3G04920
AT2G47390	AT3G04940
AT2G47410	AT3G04980
AT2G47470	AT3G05000
AT2G47510	AT3G05010
AT2G47600	AT3G05030
AT2G47610	AT3G05040
AT2G47630	AT3G05050
AT2G47680	AT3G05060
AT2G47700	AT3G05090
AT2G47820	AT3G05130
AT2G47850	AT3G05250
AT2G47900	AT3G05280
AT2G47960	AT3G05340
AT2G47980	AT3G05350
AT2G48060	AT3G05380
AT2G48075	AT3G05420
AT2G48100	AT3G05430
AT2G48110	AT3G05500
AT2G48160	AT3G05520
AT3G01090	AT3G05530
AT3G01100	AT3G05545
AT3G01120	AT3G05590
AT3G01150	AT3G05630

AT3G01280	AT3G05670
AT3G01310	AT3G05680
AT3G01320	AT3G05700
AT3G01340	AT3G05710
AT3G01350	AT3G05720
AT3G01380	AT3G05760
AT3G01390	AT3G05770
AT3G01450	AT3G05840
AT3G01460	AT3G05910
AT3G01540	AT3G05940
AT3G01560	AT3G05970
AT3G01590	AT3G06010
AT3G01650	AT3G06040
AT3G01690	AT3G06060
AT3G01720	AT3G06080
AT3G01790	AT3G06170
AT3G01810	AT3G06180
AT3G01820	AT3G06240
AT3G01910	AT3G06250
AT3G01930	AT3G06290
AT3G02070	AT3G06340
AT3G02080	AT3G06350
AT3G02090	AT3G06380
AT3G02130	AT3G06400
AT3G02170	AT3G06410
AT3G02200	AT3G06450
AT3G02230	AT3G06480
AT3G02250	AT3G06500
AT3G02260	AT3G06530
AT3G02280	AT3G06540
AT3G02290	AT3G06545
AT3G02300	AT3G06550
AT3G02320	AT3G06560
AT3G02350	AT3G06620
AT3G02360	AT3G06640
AT3G02380	AT3G06650
AT3G02420	AT3G06670
AT3G02450	AT3G06690
AT3G02460	AT3G06720
AT3G02468	AT3G06760
AT3G02470	AT3G06790
AT3G02510	AT3G06810
AT3G02520	AT3G06820
AT3G02530	AT3G06860
AT3G02540	AT3G06930
AT3G02555	AT3G06960
AT3G02560	AT3G07030
AT3G02580	AT3G07040
AT3G02630	AT3G07050
AT3G02660	AT3G07060
AT3G02680	AT3G07080
AT3G02700	AT3G07090
AT3G02720	AT3G07100
AT3G02780	AT3G07110
AT3G02860	AT3G07150
AT3G02890	AT3G07160
AT3G02900	AT3G07170
AT3G02930	AT3G07180
AT3G02950	AT3G07270
AT3G03010	AT3G07300
AT3G03050	AT3G07330
AT3G03060	AT3G07400
AT3G03070	AT3G07420
AT3G03100	AT3G07530
AT3G03110	AT3G07560
AT3G03120	AT3G07565
AT3G03140	AT3G07600
AT3G03250	AT3G07610
AT3G03300	AT3G07630
AT3G03305	AT3G07640
AT3G03310	AT3G07650
AT3G03340	AT3G07660
AT3G03350	AT3G07680
AT3G03360	AT3G07690
AT3G03380	AT3G07700
AT3G03560	AT3G07740
AT3G03570	AT3G07750
AT3G03610	AT3G07760
AT3G03710	AT3G07770
AT3G03773	AT3G07780
AT3G03790	AT3G07880
AT3G03810	AT3G07890
AT3G03860	AT3G07900
AT3G03900	AT3G07950
AT3G03940	AT3G07980
AT3G03950	AT3G08000
AT3G03960	AT3G08020
AT3G03970	AT3G08510
AT3G04080	AT3G08530
AT3G04090	AT3G08580
AT3G04120	AT3G08590
AT3G04240	AT3G08610
AT3G04300	AT3G08620
AT3G04340	AT3G08650
AT3G04350	AT3G08670
AT3G04460	AT3G08680
AT3G04480	AT3G08730
AT3G04490	AT3G08760
AT3G04500	AT3G08800
AT3G04580	AT3G08820
AT3G04590	AT3G08840
AT3G04600	AT3G08850
AT3G04605	AT3G08930
AT3G04610	AT3G08943
AT3G04680	AT3G08950
AT3G04700	AT3G08960
AT3G04710	AT3G08980
AT3G04740	AT3G08990
AT3G04780	AT3G09000
AT3G04810	AT3G09060
AT3G04820	AT3G09090
AT3G04830	AT3G09100
AT3G04840	AT3G09190
AT3G04850	AT3G09200
AT3G04870	AT3G09230
AT3G04910	AT3G09300
AT3G04920	AT3G09350
AT3G04970	AT3G09360
AT3G04980	AT3G09370
AT3G05000	AT3G09410
AT3G05010	AT3G09440
AT3G05040	AT3G09470
AT3G05050	AT3G09660

AT3G05060	AT3G09670
AT3G05070	AT3G09690
AT3G05090	AT3G09720
AT3G05120	AT3G09735
AT3G05210	AT3G09740
AT3G05250	AT3G09810
AT3G05270	AT3G09820
AT3G05280	AT3G09840
AT3G05350	AT3G09850
AT3G05360	AT3G09880
AT3G05380	AT3G09890
AT3G05420	AT3G09980
AT3G05500	AT3G10070
AT3G05510	AT3G10090
AT3G05520	AT3G10160
AT3G05530	AT3G10180
AT3G05545	AT3G10220
AT3G05570	AT3G10230
AT3G05590	AT3G10240
AT3G05670	AT3G10250
AT3G05680	AT3G10270
AT3G05700	AT3G10330
AT3G05710	AT3G10370
AT3G05750	AT3G10380
AT3G05840	AT3G10410
AT3G05850	AT3G10430
AT3G05870	AT3G10490
AT3G05900	AT3G10500
AT3G05910	AT3G10530
AT3G05930	AT3G10550
AT3G05932	AT3G10610
AT3G05940	AT3G10650
AT3G05970	AT3G10670
AT3G06010	AT3G10680
AT3G06040	AT3G10690
AT3G06080	AT3G10700
AT3G06170	AT3G10770
AT3G06250	AT3G10810
AT3G06290	AT3G10820
AT3G06330	AT3G10845
AT3G06340	AT3G10850
AT3G06350	AT3G10915
AT3G06400	AT3G10920
AT3G06440	AT3G10970
AT3G06450	AT3G11040
AT3G06480	AT3G11070
AT3G06500	AT3G11080
AT3G06510	AT3G11130
AT3G06530	AT3G11200
AT3G06550	AT3G11220
AT3G06580	AT3G11230
AT3G06620	AT3G11290
AT3G06650	AT3G11320
AT3G06660	AT3G11330
AT3G06665	AT3G11397
AT3G06670	AT3G11400
AT3G06690	AT3G11440
AT3G06720	AT3G11450
AT3G06810	AT3G11460
AT3G06820	AT3G11490
AT3G06860	AT3G11510
AT3G06920	AT3G11530
AT3G06930	AT3G11540
AT3G06940	AT3G11560
AT3G07020	AT3G11570
AT3G07030	AT3G11590
AT3G07050	AT3G11630
AT3G07060	AT3G11670
AT3G07080	AT3G11700
AT3G07090	AT3G11710
AT3G07100	AT3G11730
AT3G07110	AT3G11760
AT3G07140	AT3G11800
AT3G07160	AT3G11830
AT3G07170	AT3G11840
AT3G07215	AT3G11890
AT3G07273	AT3G11910
AT3G07274	AT3G11940
AT3G07280	AT3G11960
AT3G07300	AT3G11964
AT3G07330	AT3G12010
AT3G07370	AT3G12020
AT3G07400	AT3G12050
AT3G07530	AT3G12100
AT3G07560	AT3G12120
AT3G07600	AT3G12130
AT3G07610	AT3G12140
AT3G07630	AT3G12150
AT3G07640	AT3G12200
AT3G07650	AT3G12220
AT3G07660	AT3G12250
AT3G07700	AT3G12270
AT3G07720	AT3G12280
AT3G07740	AT3G12290
AT3G07760	AT3G12340
AT3G07770	AT3G12360
AT3G07780	AT3G12380
AT3G07790	AT3G12390
AT3G07890	AT3G12480
AT3G07930	AT3G12520
AT3G07950	AT3G12560
AT3G07980	AT3G12570
AT3G08510	AT3G12580
AT3G08530	AT3G12590
AT3G08550	AT3G12600
AT3G08580	AT3G12640
AT3G08620	AT3G12660
AT3G08650	AT3G12670
AT3G08670	AT3G12680
AT3G08680	AT3G12685
AT3G08720	AT3G12740
AT3G08730	AT3G12760
AT3G08760	AT3G12770
AT3G08840	AT3G12780
AT3G08850	AT3G12810
AT3G08930	AT3G12930
AT3G08943	AT3G12950
AT3G08960	AT3G12960
AT3G08980	AT3G12980
AT3G08990	AT3G13000
AT3G09000	AT3G13030
AT3G09040	AT3G13040
AT3G09050	AT3G13060

AT3G09090	AT3G13062
AT3G09100	AT3G13065
AT3G09180	AT3G13070
AT3G09200	AT3G13080
AT3G09300	AT3G13180
AT3G09320	AT3G13190
AT3G09350	AT3G13200
AT3G09360	AT3G13222
AT3G09370	AT3G13225
AT3G09410	AT3G13230
AT3G09440	AT3G13235
AT3G09470	AT3G13290
AT3G09560	AT3G13300
AT3G09670	AT3G13320
AT3G09710	AT3G13330
AT3G09720	AT3G13340
AT3G09735	AT3G13360
AT3G09740	AT3G13410
AT3G09810	AT3G13440
AT3G09820	AT3G13450
AT3G09830	AT3G13460
AT3G09840	AT3G13470
AT3G09863	AT3G13490
AT3G09880	AT3G13530
AT3G09890	AT3G13570
AT3G09900	AT3G13580
AT3G09980	AT3G13590
AT3G10050	AT3G13610
AT3G10140	AT3G13672
AT3G10160	AT3G13750
AT3G10210	AT3G13770
AT3G10250	AT3G13772
AT3G10270	AT3G13800
AT3G10380	AT3G13845
AT3G10410	AT3G13860
AT3G10440	AT3G13870
AT3G10480	AT3G13880
AT3G10490	AT3G13882
AT3G10530	AT3G13900
AT3G10540	AT3G13920
AT3G10550	AT3G13930
AT3G10640	AT3G13940
AT3G10650	AT3G13990
AT3G10700	AT3G14000
AT3G10770	AT3G14010
AT3G10810	AT3G14030
AT3G10850	AT3G14040
AT3G10915	AT3G14050
AT3G10920	AT3G14075
AT3G11040	AT3G14080
AT3G11130	AT3G14100
AT3G11200	AT3G14110
AT3G11220	AT3G14120
AT3G11230	AT3G14130
AT3G11240	AT3G14172
AT3G11270	AT3G14200
AT3G11290	AT3G14205
AT3G11400	AT3G14290
AT3G11440	AT3G14330
AT3G11450	AT3G14340
AT3G11510	AT3G14350
AT3G11530	AT3G14390
AT3G11540	AT3G14400
AT3G11560	AT3G14470
AT3G11590	AT3G14570
AT3G11630	AT3G14580
AT3G11710	AT3G14600
AT3G11730	AT3G14610
AT3G11760	AT3G14630
AT3G11770	AT3G14650
AT3G11830	AT3G14720
AT3G11890	AT3G14730
AT3G11910	AT3G14790
AT3G11940	AT3G14830
AT3G11945	AT3G14840
AT3G11960	AT3G14870
AT3G11964	AT3G14890
AT3G12010	AT3G14910
AT3G12012	AT3G14930
AT3G12020	AT3G14940
AT3G12130	AT3G14980
AT3G12140	AT3G15000
AT3G12180	AT3G15070
AT3G12200	AT3G15080
AT3G12250	AT3G15090
AT3G12260	AT3G15120
AT3G12280	AT3G15140
AT3G12290	AT3G15160
AT3G12360	AT3G15180
AT3G12380	AT3G15190
AT3G12480	AT3G15220
AT3G12490	AT3G15260
AT3G12520	AT3G15350
AT3G12550	AT3G15354
AT3G12560	AT3G15380
AT3G12570	AT3G15410
AT3G12590	AT3G15430
AT3G12600	AT3G15460
AT3G12620	AT3G15480
AT3G12640	AT3G15580
AT3G12670	AT3G15605
AT3G12680	AT3G15610
AT3G12690	AT3G15640
AT3G12740	AT3G15660
AT3G12760	AT3G15730
AT3G12810	AT3G15740
AT3G12950	AT3G15750
AT3G12980	AT3G15880
AT3G13000	AT3G15920
AT3G13040	AT3G15930
AT3G13050	AT3G15940
AT3G13060	AT3G15950
AT3G13061	AT3G15970
AT3G13080	AT3G15980
AT3G13180	AT3G16000
AT3G13200	AT3G16060
AT3G13205	AT3G16090
AT3G13222	AT3G16110
AT3G13225	AT3G16170
AT3G13226	AT3G16200
AT3G13230	AT3G16230
AT3G13235	AT3G16260
AT3G13270	AT3G16270

AT3G13290	AT3G16290
AT3G13300	AT3G16310
AT3G13320	AT3G16610
AT3G13330	AT3G16630
AT3G13340	AT3G16640
AT3G13350	AT3G16700
AT3G13360	AT3G16780
AT3G13410	AT3G16785
AT3G13440	AT3G16810
AT3G13460	AT3G16830
AT3G13470	AT3G16840
AT3G13490	AT3G16857
AT3G13530	AT3G16910
AT3G13570	AT3G16940
AT3G13580	AT3G16950
AT3G13670	AT3G17000
AT3G13672	AT3G17090
AT3G13740	AT3G17170
AT3G13750	AT3G17205
AT3G13772	AT3G17240
AT3G13800	AT3G17300
AT3G13860	AT3G17310
AT3G13870	AT3G17330
AT3G13920	AT3G17360
AT3G13930	AT3G17365
AT3G13990	AT3G17430
AT3G14010	AT3G17450
AT3G14075	AT3G17650
AT3G14100	AT3G17675
AT3G14110	AT3G17740
AT3G14120	AT3G17750
AT3G14172	AT3G17760
AT3G14200	AT3G17770
AT3G14205	AT3G17810
AT3G14270	AT3G17820
AT3G14290	AT3G17840
AT3G14350	AT3G17850
AT3G14400	AT3G17880
AT3G14410	AT3G17900
AT3G14470	AT3G17910
AT3G14600	AT3G17920
AT3G14610	AT3G17970
AT3G14650	AT3G18000
AT3G14720	AT3G18020
AT3G14790	AT3G18035
AT3G14800	AT3G18060
AT3G14840	AT3G18090
AT3G14860	AT3G18140
AT3G14870	AT3G18160
AT3G14930	AT3G18165
AT3G14980	AT3G18190
AT3G15070	AT3G18210
AT3G15080	AT3G18230
AT3G15090	AT3G18250
AT3G15120	AT3G18290
AT3G15160	AT3G18310
AT3G15180	AT3G18350
AT3G15220	AT3G18370
AT3G15260	AT3G18380
AT3G15270	AT3G18390
AT3G15290	AT3G18420
AT3G15350	AT3G18480
AT3G15351	AT3G18520
AT3G15354	AT3G18524
AT3G15355	AT3G18600
AT3G15380	AT3G18620
AT3G15400	AT3G18640
AT3G15410	AT3G18680
AT3G15460	AT3G18760
AT3G15518	AT3G18780
AT3G15578	AT3G18790
AT3G15590	AT3G18820
AT3G15610	AT3G18860
AT3G15640	AT3G18870
AT3G15660	AT3G18990
AT3G15690	AT3G19050
AT3G15730	AT3G19080
AT3G15790	AT3G19100
AT3G15880	AT3G19130
AT3G15920	AT3G19170
AT3G15930	AT3G19180
AT3G15940	AT3G19190
AT3G15970	AT3G19210
AT3G15980	AT3G19250
AT3G16010	AT3G19300
AT3G16060	AT3G19330
AT3G16090	AT3G19340
AT3G16170	AT3G19420
AT3G16200	AT3G19440
AT3G16230	AT3G19450
AT3G16260	AT3G19460
AT3G16270	AT3G19480
AT3G16290	AT3G19490
AT3G16310	AT3G19515
AT3G16480	AT3G19590
AT3G16565	AT3G19620
AT3G16620	AT3G19630
AT3G16630	AT3G19640
AT3G16640	AT3G19650
AT3G16710	AT3G19670
AT3G16712	AT3G19720
AT3G16730	AT3G19740
AT3G16780	AT3G19770
AT3G16785	AT3G19780
AT3G16810	AT3G19790
AT3G16830	AT3G19820
AT3G16840	AT3G19840
AT3G16857	AT3G19860
AT3G16910	AT3G19895
AT3G16940	AT3G19910
AT3G17000	AT3G19920
AT3G17090	AT3G19950
AT3G17205	AT3G19960
AT3G17220	AT3G19970
AT3G17240	AT3G19980
AT3G17250	AT3G20000
AT3G17300	AT3G20010
AT3G17310	AT3G20020
AT3G17330	AT3G20040
AT3G17340	AT3G20050
AT3G17365	AT3G20130
AT3G17410	AT3G20240
AT3G17430	AT3G20250

AT3G17440	AT3G20290
AT3G17450	AT3G20320
AT3G17465	AT3G20350
AT3G17510	AT3G20390
AT3G17590	AT3G20430
AT3G17630	AT3G20440
AT3G17650	AT3G20510
AT3G17675	AT3G20540
AT3G17680	AT3G20550
AT3G17740	AT3G20560
AT3G17750	AT3G20620
AT3G17770	AT3G20630
AT3G17820	AT3G20650
AT3G17840	AT3G20720
AT3G17850	AT3G20730
AT3G17880	AT3G20740
AT3G17900	AT3G20770
AT3G17970	AT3G20780
AT3G17998	AT3G20800
AT3G18000	AT3G20810
AT3G18040	AT3G20840
AT3G18060	AT3G20910
AT3G18090	AT3G20920
AT3G18145	AT3G20970
AT3G18165	AT3G21070
AT3G18190	AT3G21100
AT3G18290	AT3G21140
AT3G18300	AT3G21160
AT3G18350	AT3G21175
AT3G18370	AT3G21200
AT3G18380	AT3G21230
AT3G18440	AT3G21295
AT3G18480	AT3G21350
AT3G18520	AT3G21390
AT3G18524	AT3G21400
AT3G18600	AT3G21430
AT3G18640	AT3G21470
AT3G18750	AT3G21480
AT3G18770	AT3G21540
AT3G18780	AT3G21610
AT3G18820	AT3G21640
AT3G18860	AT3G21820
AT3G18890	AT3G21865
AT3G18940	AT3G22110
AT3G18990	AT3G22150
AT3G19050	AT3G22180
AT3G19100	AT3G22200
AT3G19130	AT3G22290
AT3G19170	AT3G22310
AT3G19180	AT3G22330
AT3G19190	AT3G22380
AT3G19260	AT3G22436
AT3G19290	AT3G22470
AT3G19300	AT3G22480
AT3G19330	AT3G22520
AT3G19360	AT3G22630
AT3G19420	AT3G22650
AT3G19460	AT3G22660
AT3G19490	AT3G22690
AT3G19510	AT3G22780
AT3G19515	AT3G22790
AT3G19630	AT3G22845
AT3G19650	AT3G22880
AT3G19670	AT3G22900
AT3G19720	AT3G22942
AT3G19740	AT3G22950
AT3G19760	AT3G22960
AT3G19770	AT3G22961
AT3G19780	AT3G22980
AT3G19820	AT3G22990
AT3G19840	AT3G23150
AT3G19860	AT3G23280
AT3G19900	AT3G23300
AT3G19910	AT3G23310
AT3G19960	AT3G23330
AT3G19970	AT3G23480
AT3G19980	AT3G23490
AT3G20000	AT3G23540
AT3G20010	AT3G23550
AT3G20020	AT3G23580
AT3G20050	AT3G23590
AT3G20060	AT3G23620
AT3G20083	AT3G23640
AT3G20090	AT3G23660
AT3G20130	AT3G23690
AT3G20250	AT3G23750
AT3G20290	AT3G23780
AT3G20320	AT3G23830
AT3G20330	AT3G23890
AT3G20430	AT3G23920
AT3G20500	AT3G23940
AT3G20540	AT3G23980
AT3G20541	AT3G23990
AT3G20550	AT3G24000
AT3G20560	AT3G24010
AT3G20570	AT3G24040
AT3G20620	AT3G24120
AT3G20630	AT3G24160
AT3G20650	AT3G24170
AT3G20720	AT3G24180
AT3G20730	AT3G24190
AT3G20740	AT3G24200
AT3G20770	AT3G24240
AT3G20780	AT3G24320
AT3G20800	AT3G24350
AT3G20870	AT3G24360
AT3G20890	AT3G24440
AT3G20910	AT3G24503
AT3G20920	AT3G24530
AT3G20935	AT3G24550
AT3G21100	AT3G24560
AT3G21160	AT3G24570
AT3G21175	AT3G24740
AT3G21190	AT3G24830
AT3G21215	AT3G24870
AT3G21250	AT3G24900
AT3G21280	AT3G25020
AT3G21290	AT3G25040
AT3G21351	AT3G25110
AT3G21430	AT3G25150
AT3G21465	AT3G25220
AT3G21480	AT3G25221
AT3G21540	AT3G25230

AT3G21640	AT3G25430
AT3G21740	AT3G25470
AT3G21810	AT3G25500
AT3G21820	AT3G25520
AT3G21865	AT3G25680
AT3G22110	AT3G25800
AT3G22170	AT3G25840
AT3G22180	AT3G25940
AT3G22183	AT3G26000
AT3G22190	AT3G26090
AT3G22200	AT3G26480
AT3G22220	AT3G26540
AT3G22270	AT3G26670
AT3G22290	AT3G26730
AT3G22330	AT3G26780
AT3G22380	AT3G26782
AT3G22436	AT3G26810
AT3G22440	AT3G26840
AT3G22480	AT3G26890
AT3G22520	AT3G26910
AT3G22620	AT3G26935
AT3G22660	AT3G26980
AT3G22780	AT3G26990
AT3G22790	AT3G27110
AT3G22850	AT3G27190
AT3G22942	AT3G27240
AT3G22945	AT3G27260
AT3G22950	AT3G27300
AT3G22960	AT3G27320
AT3G22980	AT3G27325
AT3G22990	AT3G27380
AT3G23070	AT3G27430
AT3G23210	AT3G27460
AT3G23280	AT3G27530
AT3G23300	AT3G27560
AT3G23310	AT3G27570
AT3G23326	AT3G27670
AT3G23330	AT3G27700
AT3G23540	AT3G27740
AT3G23580	AT3G27770
AT3G23590	AT3G27820
AT3G23600	AT3G27890
AT3G23620	AT3G27980
AT3G23640	AT3G28370
AT3G23660	AT3G28380
AT3G23780	AT3G28430
AT3G23830	AT3G28480
AT3G23910	AT3G28690
AT3G23940	AT3G28700
AT3G23980	AT3G28710
AT3G23990	AT3G28715
AT3G24005	AT3G28730
AT3G24010	AT3G28740
AT3G24020	AT3G28860
AT3G24040	AT3G28900
AT3G24120	AT3G28970
AT3G24160	AT3G29160
AT3G24170	AT3G29170
AT3G24180	AT3G29180
AT3G24190	AT3G29200
AT3G24200	AT3G29270
AT3G24320	AT3G29280
AT3G24350	AT3G29320
AT3G24440	AT3G29390
AT3G24530	AT3G30300
AT3G24550	AT3G30380
AT3G24570	AT3G32940
AT3G24740	AT3G33530
AT3G24800	AT3G42050
AT3G24820	AT3G42150
AT3G24830	AT3G42170
AT3G24870	AT3G42660
AT3G24880	AT3G42670
AT3G24890	AT3G42790
AT3G24900	AT3G42860
AT3G24929	AT3G42950
AT3G24982	AT3G43190
AT3G25010	AT3G43210
AT3G25013	AT3G43220
AT3G25014	AT3G43230
AT3G25020	AT3G43240
AT3G25040	AT3G43300
AT3G25110	AT3G43440
AT3G25120	AT3G43610
AT3G25150	AT3G43810
AT3G25220	AT3G43980
AT3G25230	AT3G44110
AT3G25430	AT3G44200
AT3G25440	AT3G44330
AT3G25470	AT3G44340
AT3G25520	AT3G44370
AT3G25545	AT3G44530
AT3G25585	AT3G44590
AT3G25680	AT3G44600
AT3G25690	AT3G44680
AT3G25800	AT3G44850
AT3G25840	AT3G45040
AT3G25910	AT3G45090
AT3G26000	AT3G45190
AT3G26020	AT3G45240
AT3G26040	AT3G45600
AT3G26090	AT3G45620
AT3G26100	AT3G45630
AT3G26340	AT3G45740
AT3G26350	AT3G45750
AT3G26370	AT3G45850
AT3G26539	AT3G46040
AT3G26670	AT3G46060
AT3G26720	AT3G46100
AT3G26810	AT3G46180
AT3G26890	AT3G46210
AT3G26900	AT3G46440
AT3G26910	AT3G46450
AT3G26920	AT3G46510
AT3G26935	AT3G46560
AT3G26950	AT3G46590
AT3G26990	AT3G46680
AT3G27000	AT3G46740
AT3G27025	AT3G46790
AT3G27080	AT3G46830
AT3G27090	AT3G46870
AT3G27120	AT3G46920
AT3G27190	AT3G46970

AT3G27210	AT3G47160
AT3G27240	AT3G47370
AT3G27260	AT3G47390
AT3G27300	AT3G47530
AT3G27310	AT3G47550
AT3G27320	AT3G47680
AT3G27325	AT3G47730
AT3G27390	AT3G47780
AT3G27420	AT3G47810
AT3G27430	AT3G47833
AT3G27470	AT3G47836
AT3G27530	AT3G47840
AT3G27570	AT3G47910
AT3G27610	AT3G47990
AT3G27670	AT3G48000
AT3G27700	AT3G48030
AT3G27740	AT3G48040
AT3G27820	AT3G48080
AT3G27925	AT3G48110
AT3G27980	AT3G48140
AT3G28030	AT3G48150
AT3G28150	AT3G48160
AT3G28370	AT3G48170
AT3G28430	AT3G48190
AT3G28630	AT3G48195
AT3G28660	AT3G48320
AT3G28670	AT3G48380
AT3G28690	AT3G48400
AT3G28700	AT3G48430
AT3G28710	AT3G48530
AT3G28715	AT3G48560
AT3G28730	AT3G48670
AT3G28750	AT3G48680
AT3G28760	AT3G48710
AT3G28860	AT3G48730
AT3G28900	AT3G48750
AT3G28956	AT3G48760
AT3G28970	AT3G48780
AT3G29095	AT3G48820
AT3G29100	AT3G48860
AT3G29120	AT3G48870
AT3G29130	AT3G48880
AT3G29160	AT3G48890
AT3G29170	AT3G49000
AT3G29180	AT3G49060
AT3G29270	AT3G49080
AT3G29320	AT3G49100
AT3G29360	AT3G49130
AT3G29390	AT3G49140
AT3G29400	AT3G49142
AT3G29760	AT3G49290
AT3G29810	AT3G49310
AT3G30300	AT3G49320
AT3G30380	AT3G49390
AT3G30390	AT3G49430
AT3G30841	AT3G49470
AT3G32940	AT3G49490
AT3G33154	AT3G49500
AT3G33530	AT3G49530
AT3G39935	AT3G49590
AT3G40640	AT3G49600
AT3G42050	AT3G49630
AT3G42052	AT3G49640
AT3G42150	AT3G49680
AT3G42170	AT3G49720
AT3G42253	AT3G49725
AT3G42630	AT3G49740
AT3G42640	AT3G49800
AT3G42670	AT3G49990
AT3G42730	AT3G50000
AT3G42790	AT3G50050
AT3G42950	AT3G50080
AT3G43210	AT3G50110
AT3G43220	AT3G50230
AT3G43230	AT3G50240
AT3G43240	AT3G50360
AT3G43300	AT3G50370
AT3G43440	AT3G50380
AT3G43510	AT3G50420
AT3G43590	AT3G50440
AT3G43600	AT3G50500
AT3G43610	AT3G50530
AT3G43810	AT3G50590
AT3G43955	AT3G50670
AT3G44010	AT3G50910
AT3G44110	AT3G50920
AT3G44160	AT3G51010
AT3G44200	AT3G51030
AT3G44280	AT3G51050
AT3G44330	AT3G51100
AT3G44340	AT3G51120
AT3G44370	AT3G51130
AT3G44480	AT3G51260
AT3G44530	AT3G51270
AT3G44600	AT3G51290
AT3G44670	AT3G51310
AT3G44680	AT3G51370
AT3G44740	AT3G51400
AT3G44750	AT3G51460
AT3G44850	AT3G51480
AT3G45020	AT3G51490
AT3G45040	AT3G51530
AT3G45090	AT3G51550
AT3G45100	AT3G51570
AT3G45190	AT3G51580
AT3G45240	AT3G51610
AT3G45242	AT3G51620
AT3G45300	AT3G51770
AT3G45400	AT3G51800
AT3G45420	AT3G51840
AT3G45620	AT3G51850
AT3G45630	AT3G51950
AT3G45650	AT3G52030
AT3G45740	AT3G52100
AT3G45750	AT3G52120
AT3G45830	AT3G52140
AT3G45850	AT3G52170
AT3G46000	AT3G52180
AT3G46010	AT3G52190
AT3G46040	AT3G52200
AT3G46060	AT3G52210
AT3G46100	AT3G52240
AT3G46180	AT3G52250

AT3G46200
AT3G46220
AT3G46290
AT3G46440
AT3G46450
AT3G46460
AT3G46510
AT3G46590
AT3G46660
AT3G46668
AT3G46670
AT3G46680
AT3G46830
AT3G46920
AT3G46930
AT3G46960
AT3G46970
AT3G46980
AT3G47060
AT3G47070
AT3G47110
AT3G47160
AT3G47370
AT3G47390
AT3G47520
AT3G47550
AT3G47610
AT3G47630
AT3G47640
AT3G47680
AT3G47690
AT3G47700
AT3G47730
AT3G47810
AT3G47833
AT3G47836
AT3G47890
AT3G47910
AT3G47990
AT3G48030
AT3G48050
AT3G48060
AT3G48110
AT3G48140
AT3G48150
AT3G48170
AT3G48190
AT3G48195
AT3G48380
AT3G48430
AT3G48470
AT3G48530
AT3G48560
AT3G48580
AT3G48670
AT3G48730
AT3G48750
AT3G48760
AT3G48780
AT3G48820
AT3G48860
AT3G48870
AT3G48880
AT3G48890
AT3G48930
AT3G49000
AT3G49010
AT3G49050
AT3G49060
AT3G49100
AT3G49140
AT3G49250
AT3G49310
AT3G49350
AT3G49390
AT3G49400
AT3G49420
AT3G49430
AT3G49490
AT3G49500
AT3G49530
AT3G49590
AT3G49600
AT3G49601
AT3G49640
AT3G49645
AT3G49650
AT3G49720
AT3G49725
AT3G49870
AT3G49880
AT3G49890
AT3G49920
AT3G49990
AT3G50000
AT3G50050
AT3G50110
AT3G50240
AT3G50360
AT3G50370
AT3G50380
AT3G50430
AT3G50500
AT3G50520
AT3G50530
AT3G50590
AT3G50670
AT3G50910
AT3G50950
AT3G50960
AT3G51010
AT3G51040
AT3G51050
AT3G51100
AT3G51120
AT3G51130
AT3G51140
AT3G51180
AT3G51260
AT3G51270
AT3G51310
AT3G51370
AT3G51390
AT3G52260
AT3G52280
AT3G52300
AT3G52560
AT3G52580
AT3G52610
AT3G52640
AT3G52660
AT3G52730
AT3G52850
AT3G52890
AT3G52990
AT3G53000
AT3G53090
AT3G53100
AT3G53110
AT3G53120
AT3G53190
AT3G53220
AT3G53270
AT3G53390
AT3G53490
AT3G53520
AT3G53540
AT3G53570
AT3G53580
AT3G53590
AT3G53610
AT3G53620
AT3G53630
AT3G53670
AT3G53710
AT3G53740
AT3G53750
AT3G53760
AT3G53780
AT3G53870
AT3G53890
AT3G53900
AT3G53930
AT3G53940
AT3G53970
AT3G54010
AT3G54100
AT3G54110
AT3G54130
AT3G54170
AT3G54190
AT3G54230
AT3G54240
AT3G54280
AT3G54300
AT3G54350
AT3G54360
AT3G54440
AT3G54480
AT3G54540
AT3G54560
AT3G54610
AT3G54620
AT3G54660
AT3G54670
AT3G54780
AT3G54790
AT3G54830
AT3G54840
AT3G54850
AT3G54860
AT3G54880
AT3G54920
AT3G55000
AT3G55005
AT3G55010
AT3G55020
AT3G55030
AT3G55070
AT3G55080
AT3G55140
AT3G55170
AT3G55260
AT3G55280
AT3G55320
AT3G55360
AT3G55380
AT3G55400
AT3G55410
AT3G55430
AT3G55440
AT3G55460
AT3G55480
AT3G55510
AT3G55530
AT3G55605
AT3G55610
AT3G55620
AT3G55640
AT3G55760
AT3G55770
AT3G55790
AT3G55840
AT3G55850
AT3G55960
AT3G56040
AT3G56050
AT3G56120
AT3G56130
AT3G56140
AT3G56150
AT3G56160
AT3G56190
AT3G56310
AT3G56320
AT3G56370
AT3G56410
AT3G56450
AT3G56460
AT3G56480
AT3G56550
AT3G56570
AT3G56590
AT3G56680
AT3G56690
AT3G56720

AT3G51460	AT3G56740
AT3G51490	AT3G56800
AT3G51550	AT3G56820
AT3G51620	AT3G56840
AT3G51640	AT3G56850
AT3G51650	AT3G56910
AT3G51770	AT3G56990
AT3G51800	AT3G57030
AT3G51830	AT3G57050
AT3G51840	AT3G57090
AT3G51850	AT3G57140
AT3G51880	AT3G57150
AT3G51970	AT3G57170
AT3G52050	AT3G57230
AT3G52100	AT3G57280
AT3G52120	AT3G57300
AT3G52130	AT3G57350
AT3G52140	AT3G57410
AT3G52160	AT3G57470
AT3G52170	AT3G57480
AT3G52180	AT3G57570
AT3G52190	AT3G57610
AT3G52200	AT3G57630
AT3G52220	AT3G57650
AT3G52240	AT3G57660
AT3G52250	AT3G57700
AT3G52260	AT3G57870
AT3G52280	AT3G57880
AT3G52300	AT3G57890
AT3G52560	AT3G57940
AT3G52580	AT3G58030
AT3G52640	AT3G58040
AT3G52660	AT3G58090
AT3G52730	AT3G58110
AT3G52750	AT3G58180
AT3G52880	AT3G58430
AT3G52890	AT3G58450
AT3G52900	AT3G58460
AT3G52930	AT3G58470
AT3G52940	AT3G58500
AT3G52950	AT3G58510
AT3G52990	AT3G58530
AT3G53020	AT3G58560
AT3G53030	AT3G58570
AT3G53090	AT3G58580
AT3G53100	AT3G58590
AT3G53120	AT3G58600
AT3G53240	AT3G58610
AT3G53270	AT3G58630
AT3G53360	AT3G58640
AT3G53365	AT3G58660
AT3G53390	AT3G58680
AT3G53500	AT3G58690
AT3G53540	AT3G58750
AT3G53570	AT3G58790
AT3G53610	AT3G58910
AT3G53750	AT3G58970
AT3G53760	AT3G59020
AT3G53930	AT3G59090
AT3G54010	AT3G59110
AT3G54110	AT3G59120
AT3G54130	AT3G59180
AT3G54230	AT3G59280
AT3G54240	AT3G59290
AT3G54280	AT3G59300
AT3G54300	AT3G59360
AT3G54350	AT3G59500
AT3G54360	AT3G59600
AT3G54440	AT3G59630
AT3G54480	AT3G59660
AT3G54540	AT3G59670
AT3G54610	AT3G59750
AT3G54660	AT3G59770
AT3G54670	AT3G59780
AT3G54760	AT3G59800
AT3G54780	AT3G59820
AT3G54790	AT3G59870
AT3G54840	AT3G59910
AT3G54860	AT3G59920
AT3G55000	AT3G59950
AT3G55005	AT3G59970
AT3G55010	AT3G59980
AT3G55020	AT3G60030
AT3G55050	AT3G60130
AT3G55070	AT3G60190
AT3G55140	AT3G60240
AT3G55160	AT3G60260
AT3G55260	AT3G60300
AT3G55270	AT3G60318
AT3G55320	AT3G60320
AT3G55350	AT3G60340
AT3G55360	AT3G60350
AT3G55380	AT3G60360
AT3G55410	AT3G60450
AT3G55430	AT3G60500
AT3G55440	AT3G60740
AT3G55460	AT3G60750
AT3G55480	AT3G60800
AT3G55510	AT3G60810
AT3G55520	AT3G60820
AT3G55530	AT3G60850
AT3G55600	AT3G60860
AT3G55610	AT3G60910
AT3G55620	AT3G61050
AT3G55770	AT3G61060
AT3G55830	AT3G61070
AT3G55850	AT3G61110
AT3G55960	AT3G61130
AT3G56040	AT3G61140
AT3G56050	AT3G61240
AT3G56120	AT3G61380
AT3G56130	AT3G61410
AT3G56150	AT3G61415
AT3G56190	AT3G61420
AT3G56260	AT3G61480
AT3G56300	AT3G61570
AT3G56310	AT3G61600
AT3G56370	AT3G61620
AT3G56408	AT3G61690
AT3G56410	AT3G61710
AT3G56450	AT3G61730
AT3G56490	AT3G61770
AT3G56500	AT3G61790

AT3G56510	AT3G61960
AT3G56580	AT3G62010
AT3G56590	AT3G62040
AT3G56640	AT3G62120
AT3G56720	AT3G62130
AT3G56740	AT3G62150
AT3G56760	AT3G62190
AT3G56830	AT3G62240
AT3G56960	AT3G62290
AT3G56990	AT3G62360
AT3G57000	AT3G62560
AT3G57030	AT3G62830
AT3G57050	AT3G62870
AT3G57060	AT3G62890
AT3G57090	AT3G62900
AT3G57140	AT3G62940
AT3G57170	AT3G62980
AT3G57230	AT3G63030
AT3G57280	AT3G63070
AT3G57290	AT3G63130
AT3G57300	AT3G63140
AT3G57350	AT3G63150
AT3G57410	AT3G63170
AT3G57470	AT3G63180
AT3G57520	AT3G63220
AT3G57570	AT3G63250
AT3G57610	AT3G63260
AT3G57650	AT3G63300
AT3G57660	AT3G63340
AT3G57700	AT3G63400
AT3G57890	AT3G63410
AT3G57940	AT3G63460
AT3G57965	AT3G63470
AT3G57970	AT3G63500
AT3G58030	AT3G63530
AT3G58040	AT3G66654
AT3G58050	AT3G66658
AT3G58110	AT4G00026
AT3G58130	AT4G00040
AT3G58170	AT4G00200
AT3G58180	AT4G00300
AT3G58460	AT4G00350
AT3G58500	AT4G00420
AT3G58510	AT4G00450
AT3G58560	AT4G00500
AT3G58570	AT4G00550
AT3G58580	AT4G00570
AT3G58600	AT4G00630
AT3G58610	AT4G00660
AT3G58640	AT4G00720
AT3G58670	AT4G00730
AT3G58680	AT4G00740
AT3G58730	AT4G00750
AT3G58760	AT4G00752
AT3G58810	AT4G00800
AT3G58940	AT4G00830
AT3G59000	AT4G00990
AT3G59020	AT4G01100
AT3G59090	AT4G01120
AT3G59110	AT4G01210
AT3G59210	AT4G01250
AT3G59280	AT4G01290
AT3G59290	AT4G01320
AT3G59300	AT4G01370
AT3G59320	AT4G01380
AT3G59360	AT4G01400
AT3G59410	AT4G01560
AT3G59430	AT4G01610
AT3G59470	AT4G01650
AT3G59500	AT4G01690
AT3G59530	AT4G01850
AT3G59600	AT4G01860
AT3G59630	AT4G01910
AT3G59660	AT4G02010
AT3G59670	AT4G02020
AT3G59750	AT4G02030
AT3G59760	AT4G02070
AT3G59770	AT4G02220
AT3G59800	AT4G02260
AT3G59820	AT4G02400
AT3G59870	AT4G02480
AT3G59890	AT4G02560
AT3G59910	AT4G02570
AT3G59920	AT4G02580
AT3G59950	AT4G02600
AT3G59970	AT4G02610
AT3G59990	AT4G02620
AT3G60030	AT4G02640
AT3G60190	AT4G02680
AT3G60240	AT4G02840
AT3G60290	AT4G02880
AT3G60300	AT4G02920
AT3G60310	AT4G02930
AT3G60340	AT4G03020
AT3G60350	AT4G03080
AT3G60360	AT4G03090
AT3G60380	AT4G03120
AT3G60500	AT4G03180
AT3G60590	AT4G03190
AT3G60600	AT4G03220
AT3G60620	AT4G03320
AT3G60740	AT4G03390
AT3G60750	AT4G03415
AT3G60770	AT4G03520
AT3G60820	AT4G03560
AT3G60860	AT4G04040
AT3G60880	AT4G04320
AT3G61010	AT4G04340
AT3G61050	AT4G04350
AT3G61070	AT4G04360
AT3G61130	AT4G04370
AT3G61140	AT4G04460
AT3G61240	AT4G04780
AT3G61350	AT4G04800
AT3G61370	AT4G04860
AT3G61410	AT4G04885
AT3G61415	AT4G04890
AT3G61480	AT4G04910
AT3G61570	AT4G04920
AT3G61600	AT4G04940
AT3G61620	AT4G04950
AT3G61670	AT4G05071
AT3G61690	AT4G05420

AT3G61710	AT4G06634
AT3G61790	AT4G06676
AT3G61860	AT4G07410
AT3G61960	AT4G07990
AT3G61990	AT4G08170
AT3G62010	AT4G08180
AT3G62040	AT4G08210
AT3G62090	AT4G08230
AT3G62120	AT4G08310
AT3G62240	AT4G08350
AT3G62260	AT4G08390
AT3G62290	AT4G08460
AT3G62310	AT4G08470
AT3G62330	AT4G08480
AT3G62360	AT4G08500
AT3G62400	AT4G08520
AT3G62455	AT4G08580
AT3G62470	AT4G08790
AT3G62600	AT4G08810
AT3G62620	AT4G08960
AT3G62800	AT4G09020
AT3G62840	AT4G09150
AT3G62870	AT4G09320
AT3G62900	AT4G09510
AT3G62930	AT4G09570
AT3G62940	AT4G09630
AT3G62980	AT4G09640
AT3G63030	AT4G09680
AT3G63070	AT4G09720
AT3G63130	AT4G09730
AT3G63150	AT4G09800
AT3G63180	AT4G09830
AT3G63220	AT4G09980
AT3G63250	AT4G10050
AT3G63340	AT4G10070
AT3G63375	AT4G10140
AT3G63400	AT4G10180
AT3G63460	AT4G10300
AT3G63530	AT4G10320
AT3G66654	AT4G10430
AT3G66658	AT4G10450
AT4G00026	AT4G10480
AT4G00040	AT4G10570
AT4G00060	AT4G10590
AT4G00200	AT4G10610
AT4G00335	AT4G10650
AT4G00342	AT4G10710
AT4G00355	AT4G10730
AT4G00420	AT4G10760
AT4G00450	AT4G10790
AT4G00550	AT4G10840
AT4G00560	AT4G10890
AT4G00570	AT4G10970
AT4G00590	AT4G11010
AT4G00630	AT4G11110
AT4G00660	AT4G11150
AT4G00720	AT4G11160
AT4G00740	AT4G11240
AT4G00752	AT4G11250
AT4G00755	AT4G11260
AT4G00760	AT4G11270
AT4G00770	AT4G11380
AT4G00800	AT4G11420
AT4G00830	AT4G11560
AT4G00840	AT4G11600
AT4G00860	AT4G11670
AT4G00910	AT4G11690
AT4G00975	AT4G11740
AT4G00990	AT4G11790
AT4G01040	AT4G11800
AT4G01050	AT4G11820
AT4G01100	AT4G11830
AT4G01120	AT4G11840
AT4G01210	AT4G11860
AT4G01290	AT4G11876
AT4G01310	AT4G11920
AT4G01320	AT4G11970
AT4G01560	AT4G11980
AT4G01570	AT4G12230
AT4G01610	AT4G12590
AT4G01660	AT4G12600
AT4G01690	AT4G12640
AT4G01700	AT4G12680
AT4G01800	AT4G12750
AT4G01810	AT4G12770
AT4G01850	AT4G12780
AT4G01860	AT4G12790
AT4G01880	AT4G13010
AT4G01960	AT4G13020
AT4G02010	AT4G13040
AT4G02020	AT4G13170
AT4G02030	AT4G13270
AT4G02080	AT4G13280
AT4G02120	AT4G13350
AT4G02150	AT4G13400
AT4G02195	AT4G13430
AT4G02220	AT4G13540
AT4G02230	AT4G13550
AT4G02260	AT4G13590
AT4G02400	AT4G13720
AT4G02430	AT4G13730
AT4G02450	AT4G13750
AT4G02480	AT4G13780
AT4G02510	AT4G13850
AT4G02520	AT4G13930
AT4G02560	AT4G13940
AT4G02570	AT4G13970
AT4G02580	AT4G14070
AT4G02600	AT4G14104
AT4G02620	AT4G14110
AT4G02680	AT4G14145
AT4G02740	AT4G14160
AT4G02880	AT4G14170
AT4G02900	AT4G14200
AT4G02920	AT4G14220
AT4G02930	AT4G14290
AT4G02980	AT4G14310
AT4G02990	AT4G14320
AT4G03000	AT4G14340
AT4G03020	AT4G14342
AT4G03080	AT4G14350
AT4G03090	AT4G14360
AT4G03110	AT4G14385

AT4G03115	AT4G14420
AT4G03120	AT4G14500
AT4G03140	AT4G14520
AT4G03180	AT4G14615
AT4G03230	AT4G14713
AT4G03250	AT4G14720
AT4G03260	AT4G14800
AT4G03390	AT4G14820
AT4G03400	AT4G14850
AT4G03520	AT4G14880
AT4G03550	AT4G14890
AT4G03560	AT4G14920
AT4G03820	AT4G14950
AT4G03930	AT4G14960
AT4G04040	AT4G14990
AT4G04180	AT4G15010
AT4G04190	AT4G15030
AT4G04210	AT4G15080
AT4G04293	AT4G15180
AT4G04320	AT4G15200
AT4G04340	AT4G15410
AT4G04350	AT4G15470
AT4G04360	AT4G15480
AT4G04370	AT4G15520
AT4G04470	AT4G15570
AT4G04614	AT4G15640
AT4G04770	AT4G15730
AT4G04780	AT4G15790
AT4G04790	AT4G15850
AT4G04800	AT4G15880
AT4G04860	AT4G15900
AT4G04880	AT4G15930
AT4G04885	AT4G16070
AT4G04910	AT4G16100
AT4G04920	AT4G16110
AT4G04940	AT4G16120
AT4G05000	AT4G16130
AT4G05020	AT4G16143
AT4G05040	AT4G16144
AT4G05060	AT4G16150
AT4G05090	AT4G16180
AT4G05091	AT4G16250
AT4G05390	AT4G16270
AT4G05410	AT4G16280
AT4G05420	AT4G16310
AT4G05505	AT4G16330
AT4G05530	AT4G16340
AT4G05631	AT4G16360
AT4G06598	AT4G16400
AT4G06634	AT4G16420
AT4G06676	AT4G16440
AT4G07410	AT4G16530
AT4G07990	AT4G16630
AT4G08131	AT4G16650
AT4G08170	AT4G16660
AT4G08180	AT4G16700
AT4G08210	AT4G16720
AT4G08230	AT4G16760
AT4G08240	AT4G16830
AT4G08250	AT4G16845
AT4G08280	AT4G16850
AT4G08310	AT4G16970
AT4G08350	AT4G17010
AT4G08351	AT4G17040
AT4G08390	AT4G17060
AT4G08460	AT4G17080
AT4G08470	AT4G17140
AT4G08480	AT4G17150
AT4G08500	AT4G17170
AT4G08510	AT4G17190
AT4G08520	AT4G17230
AT4G08540	AT4G17270
AT4G08580	AT4G17300
AT4G08690	AT4G17310
AT4G08790	AT4G17330
AT4G08850	AT4G17390
AT4G08920	AT4G17410
AT4G08980	AT4G17486
AT4G08991	AT4G17520
AT4G09000	AT4G17610
AT4G09020	AT4G17620
AT4G09130	AT4G17650
AT4G09140	AT4G17720
AT4G09150	AT4G17740
AT4G09320	AT4G17770
AT4G09340	AT4G17830
AT4G09510	AT4G17910
AT4G09520	AT4G17960
AT4G09560	AT4G18030
AT4G09580	AT4G18040
AT4G09620	AT4G18100
AT4G09630	AT4G18120
AT4G09680	AT4G18140
AT4G09750	AT4G18260
AT4G09800	AT4G18300
AT4G09810	AT4G18520
AT4G09830	AT4G18530
AT4G09980	AT4G18593
AT4G10030	AT4G18710
AT4G10050	AT4G18890
AT4G10080	AT4G18905
AT4G10090	AT4G18975
AT4G10110	AT4G19000
AT4G10120	AT4G19020
AT4G10140	AT4G19040
AT4G10320	AT4G19090
AT4G10430	AT4G19110
AT4G10480	AT4G19112
AT4G10550	AT4G19191
AT4G10570	AT4G19210
AT4G10590	AT4G19350
AT4G10610	AT4G19450
AT4G10650	AT4G19490
AT4G10710	AT4G19540
AT4G10730	AT4G19550
AT4G10760	AT4G19600
AT4G10790	AT4G19610
AT4G10890	AT4G19640
AT4G10930	AT4G19670
AT4G10940	AT4G19710
AT4G10970	AT4G19860
AT4G11010	AT4G20010
AT4G11060	AT4G20170

AT4G11110	AT4G20330
AT4G11120	AT4G20360
AT4G11130	AT4G20380
AT4G11150	AT4G20400
AT4G11160	AT4G20410
AT4G11220	AT4G20480
AT4G11260	AT4G20770
AT4G11270	AT4G20850
AT4G11380	AT4G20900
AT4G11410	AT4G20910
AT4G11420	AT4G20930
AT4G11440	AT4G20940
AT4G11450	AT4G20960
AT4G11560	AT4G21060
AT4G11570	AT4G21090
AT4G11640	AT4G21100
AT4G11670	AT4G21120
AT4G11740	AT4G21150
AT4G11790	AT4G21160
AT4G11800	AT4G21520
AT4G11820	AT4G21580
AT4G11830	AT4G21660
AT4G11840	AT4G21670
AT4G11845	AT4G21700
AT4G11860	AT4G21710
AT4G11970	AT4G21790
AT4G12010	AT4G21810
AT4G12020	AT4G21860
AT4G12060	AT4G22000
AT4G12230	AT4G22130
AT4G12340	AT4G22140
AT4G12420	AT4G22220
AT4G12460	AT4G22290
AT4G12570	AT4G22330
AT4G12590	AT4G22340
AT4G12610	AT4G22370
AT4G12640	AT4G22540
AT4G12650	AT4G22670
AT4G12670	AT4G22720
AT4G12680	AT4G22770
AT4G12710	AT4G22940
AT4G12750	AT4G22970
AT4G12760	AT4G23000
AT4G12770	AT4G23060
AT4G12780	AT4G23100
AT4G12790	AT4G23440
AT4G13010	AT4G23460
AT4G13020	AT4G23470
AT4G13215	AT4G23490
AT4G13320	AT4G23540
AT4G13350	AT4G23620
AT4G13360	AT4G23630
AT4G13400	AT4G23640
AT4G13430	AT4G23650
AT4G13540	AT4G23710
AT4G13550	AT4G23730
AT4G13580	AT4G23840
AT4G13630	AT4G23850
AT4G13640	AT4G23890
AT4G13720	AT4G23895
AT4G13730	AT4G23910
AT4G13750	AT4G23940
AT4G13780	AT4G23980
AT4G13860	AT4G24020
AT4G13880	AT4G24100
AT4G13918	AT4G24160
AT4G13920	AT4G24175
AT4G13930	AT4G24180
AT4G13970	AT4G24190
AT4G14000	AT4G24240
AT4G14050	AT4G24270
AT4G14070	AT4G24280
AT4G14103	AT4G24290
AT4G14110	AT4G24320
AT4G14145	AT4G24330
AT4G14147	AT4G24350
AT4G14160	AT4G24380
AT4G14200	AT4G24400
AT4G14210	AT4G24440
AT4G14220	AT4G24470
AT4G14290	AT4G24480
AT4G14300	AT4G24490
AT4G14301	AT4G24510
AT4G14320	AT4G24520
AT4G14340	AT4G24530
AT4G14342	AT4G24550
AT4G14350	AT4G24580
AT4G14360	AT4G24590
AT4G14415	AT4G24600
AT4G14420	AT4G24610
AT4G14500	AT4G24620
AT4G14504	AT4G24680
AT4G14510	AT4G24690
AT4G14570	AT4G24710
AT4G14600	AT4G24730
AT4G14605	AT4G24740
AT4G14720	AT4G24760
AT4G14760	AT4G24770
AT4G14880	AT4G24790
AT4G14905	AT4G24800
AT4G14920	AT4G24820
AT4G14950	AT4G24830
AT4G14960	AT4G24840
AT4G14990	AT4G24880
AT4G15010	AT4G24900
AT4G15020	AT4G24920
AT4G15030	AT4G24940
AT4G15080	AT4G24970
AT4G15110	AT4G24990
AT4G15165	AT4G25030
AT4G15180	AT4G25100
AT4G15215	AT4G25120
AT4G15410	AT4G25130
AT4G15415	AT4G25230
AT4G15470	AT4G25240
AT4G15475	AT4G25270
AT4G15520	AT4G25315
AT4G15540	AT4G25320
AT4G15560	AT4G25340
AT4G15570	AT4G25360
AT4G15640	AT4G25370
AT4G15780	AT4G25450
AT4G15790	AT4G25500

AT4G15802	AT4G25510
AT4G15850	AT4G25515
AT4G15900	AT4G25520
AT4G16070	AT4G25530
AT4G16110	AT4G25540
AT4G16130	AT4G25550
AT4G16143	AT4G25570
AT4G16144	AT4G25600
AT4G16150	AT4G25610
AT4G16180	AT4G25630
AT4G16280	AT4G25670
AT4G16310	AT4G25680
AT4G16340	AT4G25730
AT4G16390	AT4G25740
AT4G16420	AT4G25840
AT4G16440	AT4G25870
AT4G16520	AT4G25880
AT4G16570	AT4G25970
AT4G16580	AT4G25980
AT4G16630	AT4G26000
AT4G16650	AT4G26100
AT4G16660	AT4G26110
AT4G16680	AT4G26190
AT4G16720	AT4G26210
AT4G16760	AT4G26240
AT4G16800	AT4G26270
AT4G16830	AT4G26300
AT4G16845	AT4G26400
AT4G16850	AT4G26430
AT4G16960	AT4G26440
AT4G16970	AT4G26455
AT4G17010	AT4G26480
AT4G17040	AT4G26510
AT4G17050	AT4G26550
AT4G17140	AT4G26570
AT4G17170	AT4G26600
AT4G17230	AT4G26620
AT4G17270	AT4G26630
AT4G17330	AT4G26640
AT4G17390	AT4G26700
AT4G17420	AT4G26720
AT4G17486	AT4G26750
AT4G17510	AT4G26840
AT4G17520	AT4G26870
AT4G17530	AT4G26900
AT4G17540	AT4G26910
AT4G17570	AT4G26940
AT4G17610	AT4G26970
AT4G17616	AT4G26980
AT4G17620	AT4G26990
AT4G17640	AT4G27010
AT4G17720	AT4G27020
AT4G17770	AT4G27030
AT4G17830	AT4G27040
AT4G17890	AT4G27060
AT4G17910	AT4G27080
AT4G18030	AT4G27090
AT4G18040	AT4G27120
AT4G18060	AT4G27180
AT4G18120	AT4G27220
AT4G18130	AT4G27380
AT4G18140	AT4G27430
AT4G18220	AT4G27500
AT4G18240	AT4G27585
AT4G18270	AT4G27630
AT4G18395	AT4G27640
AT4G18465	AT4G27680
AT4G18530	AT4G27690
AT4G18593	AT4G27700
AT4G18596	AT4G27720
AT4G18600	AT4G27745
AT4G18730	AT4G27760
AT4G18820	AT4G27780
AT4G18830	AT4G27870
AT4G18890	AT4G27910
AT4G18905	AT4G27960
AT4G18975	AT4G28060
AT4G19006	AT4G28070
AT4G19020	AT4G28080
AT4G19040	AT4G28200
AT4G19060	AT4G28390
AT4G19070	AT4G28400
AT4G19080	AT4G28430
AT4G19090	AT4G28450
AT4G19110	AT4G28470
AT4G19112	AT4G28510
AT4G19160	AT4G28540
AT4G19180	AT4G28550
AT4G19185	AT4G28600
AT4G19190	AT4G28706
AT4G19210	AT4G28710
AT4G19350	AT4G28730
AT4G19440	AT4G28760
AT4G19490	AT4G28830
AT4G19500	AT4G28860
AT4G19550	AT4G28880
AT4G19600	AT4G28910
AT4G19610	AT4G28990
AT4G19640	AT4G29040
AT4G19670	AT4G29130
AT4G19710	AT4G29160
AT4G19829	AT4G29210
AT4G19850	AT4G29330
AT4G19860	AT4G29380
AT4G19880	AT4G29410
AT4G19990	AT4G29440
AT4G20060	AT4G29480
AT4G20325	AT4G29490
AT4G20330	AT4G29560
AT4G20380	AT4G29730
AT4G20400	AT4G29735
AT4G20410	AT4G29790
AT4G20720	AT4G29810
AT4G20850	AT4G29830
AT4G20870	AT4G29890
AT4G20910	AT4G29900
AT4G20930	AT4G29940
AT4G20940	AT4G29950
AT4G21060	AT4G29960
AT4G21100	AT4G29990
AT4G21105	AT4G30010
AT4G21110	AT4G30020
AT4G21150	AT4G30100

AT4G21160	AT4G30110
AT4G21180	AT4G30160
AT4G21323	AT4G30200
AT4G21400	AT4G30210
AT4G21450	AT4G30220
AT4G21460	AT4G30260
AT4G21470	AT4G30310
AT4G21530	AT4G30340
AT4G21550	AT4G30360
AT4G21570	AT4G30550
AT4G21580	AT4G30580
AT4G21585	AT4G30600
AT4G21660	AT4G30610
AT4G21670	AT4G30680
AT4G21710	AT4G30700
AT4G21720	AT4G30710
AT4G21790	AT4G30780
AT4G21800	AT4G30790
AT4G21810	AT4G30920
AT4G21880	AT4G30950
AT4G21900	AT4G30990
AT4G22000	AT4G31010
AT4G22120	AT4G31070
AT4G22130	AT4G31080
AT4G22140	AT4G31100
AT4G22150	AT4G31120
AT4G22217	AT4G31160
AT4G22220	AT4G31170
AT4G22280	AT4G31180
AT4G22285	AT4G31200
AT4G22310	AT4G31210
AT4G22330	AT4G31230
AT4G22340	AT4G31240
AT4G22370	AT4G31290
AT4G22540	AT4G31340
AT4G22545	AT4G31350
AT4G22670	AT4G31390
AT4G22720	AT4G31430
AT4G22740	AT4G31480
AT4G22750	AT4G31490
AT4G22760	AT4G31530
AT4G22850	AT4G31540
AT4G22930	AT4G31550
AT4G23000	AT4G31570
AT4G23040	AT4G31580
AT4G23050	AT4G31590
AT4G23100	AT4G31600
AT4G23330	AT4G31670
AT4G23430	AT4G31700
AT4G23440	AT4G31720
AT4G23460	AT4G31750
AT4G23470	AT4G31770
AT4G23630	AT4G31790
AT4G23640	AT4G31810
AT4G23650	AT4G31860
AT4G23730	AT4G31880
AT4G23790	AT4G31980
AT4G23840	AT4G31990
AT4G23850	AT4G32010
AT4G23885	AT4G32030
AT4G23890	AT4G32050
AT4G23895	AT4G32060
AT4G23900	AT4G32130
AT4G23910	AT4G32140
AT4G23980	AT4G32160
AT4G24020	AT4G32175
AT4G24100	AT4G32180
AT4G24190	AT4G32250
AT4G24200	AT4G32272
AT4G24270	AT4G32285
AT4G24280	AT4G32320
AT4G24290	AT4G32330
AT4G24400	AT4G32360
AT4G24490	AT4G32410
AT4G24520	AT4G32420
AT4G24530	AT4G32520
AT4G24550	AT4G32530
AT4G24560	AT4G32551
AT4G24590	AT4G32560
AT4G24610	AT4G32570
AT4G24620	AT4G32600
AT4G24630	AT4G32605
AT4G24680	AT4G32610
AT4G24690	AT4G32620
AT4G24740	AT4G32640
AT4G24760	AT4G32660
AT4G24790	AT4G32690
AT4G24800	AT4G32700
AT4G24830	AT4G32720
AT4G24840	AT4G32730
AT4G24900	AT4G32750
AT4G24910	AT4G32760
AT4G24940	AT4G32790
AT4G24950	AT4G32810
AT4G24990	AT4G32840
AT4G25030	AT4G32850
AT4G25120	AT4G32880
AT4G25230	AT4G32910
AT4G25240	AT4G32920
AT4G25280	AT4G32930
AT4G25315	AT4G32940
AT4G25360	AT4G32970
AT4G25450	AT4G33030
AT4G25500	AT4G33060
AT4G25515	AT4G33080
AT4G25520	AT4G33090
AT4G25550	AT4G33100
AT4G25610	AT4G33140
AT4G25631	AT4G33145
AT4G25660	AT4G33150
AT4G25670	AT4G33170
AT4G25672	AT4G33200
AT4G25680	AT4G33210
AT4G25690	AT4G33220
AT4G25692	AT4G33240
AT4G25707	AT4G33250
AT4G25719	AT4G33350
AT4G25730	AT4G33380
AT4G25740	AT4G33430
AT4G25840	AT4G33440
AT4G25870	AT4G33480
AT4G25880	AT4G33510
AT4G25970	AT4G33530

AT4G25980	AT4G33540
AT4G26000	AT4G33560
AT4G26090	AT4G33625
AT4G26095	AT4G33630
AT4G26100	AT4G33650
AT4G26110	AT4G33670
AT4G26190	AT4G33680
AT4G26210	AT4G33690
AT4G26288	AT4G33700
AT4G26300	AT4G33760
AT4G26310	AT4G33780
AT4G26400	AT4G33910
AT4G26410	AT4G33940
AT4G26455	AT4G33945
AT4G26480	AT4G34020
AT4G26510	AT4G34030
AT4G26550	AT4G34040
AT4G26570	AT4G34100
AT4G26582	AT4G34110
AT4G26590	AT4G34120
AT4G26610	AT4G34150
AT4G26620	AT4G34200
AT4G26630	AT4G34260
AT4G26640	AT4G34270
AT4G26650	AT4G34290
AT4G26690	AT4G34310
AT4G26720	AT4G34350
AT4G26750	AT4G34412
AT4G26760	AT4G34430
AT4G26840	AT4G34450
AT4G26900	AT4G34490
AT4G26910	AT4G34570
AT4G26940	AT4G34640
AT4G26965	AT4G34670
AT4G26970	AT4G34700
AT4G27010	AT4G34860
AT4G27020	AT4G34890
AT4G27030	AT4G34910
AT4G27040	AT4G34960
AT4G27080	AT4G35000
AT4G27090	AT4G35050
AT4G27130	AT4G35080
AT4G27180	AT4G35130
AT4G27380	AT4G35220
AT4G27430	AT4G35260
AT4G27470	AT4G35310
AT4G27500	AT4G35335
AT4G27585	AT4G35450
AT4G27630	AT4G35530
AT4G27640	AT4G35580
AT4G27650	AT4G35740
AT4G27680	AT4G35785
AT4G27690	AT4G35790
AT4G27720	AT4G35800
AT4G27760	AT4G35830
AT4G27870	AT4G35850
AT4G27900	AT4G35860
AT4G27910	AT4G35870
AT4G28080	AT4G35890
AT4G28220	AT4G35910
AT4G28260	AT4G35940
AT4G28300	AT4G36080
AT4G28360	AT4G36130
AT4G28370	AT4G36140
AT4G28380	AT4G36170
AT4G28390	AT4G36180
AT4G28400	AT4G36195
AT4G28405	AT4G36290
AT4G28450	AT4G36360
AT4G28470	AT4G36400
AT4G28480	AT4G36440
AT4G28510	AT4G36480
AT4G28540	AT4G36580
AT4G28550	AT4G36630
AT4G28600	AT4G36650
AT4G28610	AT4G36660
AT4G28710	AT4G36690
AT4G28760	AT4G36720
AT4G28820	AT4G36730
AT4G28860	AT4G36760
AT4G28880	AT4G36770
AT4G28910	AT4G36780
AT4G28990	AT4G36800
AT4G29010	AT4G36860
AT4G29060	AT4G36890
AT4G29130	AT4G36940
AT4G29160	AT4G36945
AT4G29330	AT4G36960
AT4G29380	AT4G36980
AT4G29410	AT4G37090
AT4G29440	AT4G37095
AT4G29480	AT4G37100
AT4G29490	AT4G37120
AT4G29730	AT4G37130
AT4G29790	AT4G37170
AT4G29810	AT4G37280
AT4G29860	AT4G37440
AT4G29880	AT4G37460
AT4G29900	AT4G37490
AT4G29940	AT4G37510
AT4G29950	AT4G37590
AT4G29990	AT4G37608
AT4G30000	AT4G37670
AT4G30020	AT4G37740
AT4G30090	AT4G37830
AT4G30100	AT4G37870
AT4G30130	AT4G37910
AT4G30150	AT4G37940
AT4G30160	AT4G38010
AT4G30190	AT4G38040
AT4G30200	AT4G38070
AT4G30210	AT4G38120
AT4G30220	AT4G38130
AT4G30240	AT4G38200
AT4G30310	AT4G38210
AT4G30360	AT4G38240
AT4G30510	AT4G38270
AT4G30560	AT4G38290
AT4G30570	AT4G38350
AT4G30580	AT4G38360
AT4G30610	AT4G38430
AT4G30620	AT4G38440
AT4G30680	AT4G38460

AT4G30710	AT4G38470
AT4G30720	AT4G38500
AT4G30780	AT4G38510
AT4G30790	AT4G38520
AT4G30810	AT4G38560
AT4G30820	AT4G38570
AT4G30890	AT4G38580
AT4G30910	AT4G38600
AT4G30920	AT4G38630
AT4G30935	AT4G38710
AT4G30950	AT4G38760
AT4G30990	AT4G38790
AT4G31030	AT4G38800
AT4G31060	AT4G38880
AT4G31120	AT4G38920
AT4G31160	AT4G39050
AT4G31170	AT4G39080
AT4G31180	AT4G39090
AT4G31200	AT4G39100
AT4G31210	AT4G39120
AT4G31230	AT4G39140
AT4G31240	AT4G39150
AT4G31265	AT4G39170
AT4G31270	AT4G39180
AT4G31300	AT4G39190
AT4G31340	AT4G39200
AT4G31350	AT4G39220
AT4G31351	AT4G39280
AT4G31370	AT4G39300
AT4G31390	AT4G39420
AT4G31420	AT4G39500
AT4G31430	AT4G39510
AT4G31440	AT4G39520
AT4G31441	AT4G39530
AT4G31450	AT4G39680
AT4G31480	AT4G39690
AT4G31490	AT4G39850
AT4G31570	AT4G39900
AT4G31580	AT4G39910
AT4G31600	AT4G39952
AT4G31700	AT4G39955
AT4G31720	AT4G39960
AT4G31740	AT4G39990
AT4G31750	AT4G40000
AT4G31770	AT4G40040
AT4G31790	AT4G40050
AT4G31810	AT5G01010
AT4G31860	AT5G01020
AT4G31880	AT5G01090
AT4G31920	AT5G01270
AT4G31990	AT5G01290
AT4G32010	AT5G01350
AT4G32050	AT5G01400
AT4G32060	AT5G01450
AT4G32070	AT5G01460
AT4G32120	AT5G01480
AT4G32130	AT5G01500
AT4G32140	AT5G01620
AT4G32160	AT5G01690
AT4G32180	AT5G01720
AT4G32250	AT5G01770
AT4G32272	AT5G01800
AT4G32285	AT5G01890
AT4G32330	AT5G01950
AT4G32360	AT5G01960
AT4G32410	AT5G01980
AT4G32420	AT5G01990
AT4G32440	AT5G02250
AT4G32470	AT5G02280
AT4G32520	AT5G02290
AT4G32530	AT5G02310
AT4G32551	AT5G02320
AT4G32560	AT5G02370
AT4G32570	AT5G02450
AT4G32600	AT5G02480
AT4G32620	AT5G02500
AT4G32640	AT5G02610
AT4G32660	AT5G02670
AT4G32680	AT5G02690
AT4G32700	AT5G02760
AT4G32720	AT5G02790
AT4G32730	AT5G02870
AT4G32760	AT5G02880
AT4G32790	AT5G02960
AT4G32820	AT5G03030
AT4G32840	AT5G03040
AT4G32850	AT5G03070
AT4G32900	AT5G03160
AT4G32910	AT5G03270
AT4G32920	AT5G03280
AT4G32930	AT5G03290
AT4G32940	AT5G03300
AT4G32970	AT5G03330
AT4G33030	AT5G03340
AT4G33060	AT5G03415
AT4G33080	AT5G03420
AT4G33090	AT5G03430
AT4G33200	AT5G03450
AT4G33210	AT5G03455
AT4G33240	AT5G03470
AT4G33250	AT5G03500
AT4G33355	AT5G03520
AT4G33410	AT5G03540
AT4G33430	AT5G03650
AT4G33440	AT5G03660
AT4G33470	AT5G03730
AT4G33520	AT5G03740
AT4G33530	AT5G03800
AT4G33540	AT5G03900
AT4G33580	AT5G03910
AT4G33620	AT5G03970
AT4G33650	AT5G04040
AT4G33680	AT5G04080
AT4G33690	AT5G04090
AT4G33700	AT5G04130
AT4G33740	AT5G04170
AT4G33760	AT5G04180
AT4G33890	AT5G04240
AT4G33910	AT5G04250
AT4G33920	AT5G04260
AT4G33940	AT5G04280
AT4G33980	AT5G04290
AT4G34020	AT5G04320

AT4G34030	AT5G04410
AT4G34040	AT5G04420
AT4G34080	AT5G04430
AT4G34100	AT5G04440
AT4G34110	AT5G04460
AT4G34140	AT5G04480
AT4G34200	AT5G04510
AT4G34270	AT5G04540
AT4G34280	AT5G04560
AT4G34310	AT5G04590
AT4G34350	AT5G04600
AT4G34370	AT5G04710
AT4G34390	AT5G04720
AT4G34430	AT5G04740
AT4G34450	AT5G04750
AT4G34460	AT5G04780
AT4G34490	AT5G04885
AT4G34570	AT5G04920
AT4G34640	AT5G04930
AT4G34660	AT5G04940
AT4G34670	AT5G04990
AT4G34700	AT5G05000
AT4G34720	AT5G05010
AT4G34860	AT5G05080
AT4G34890	AT5G05100
AT4G34960	AT5G05170
AT4G35000	AT5G05210
AT4G35080	AT5G05230
AT4G35090	AT5G05240
AT4G35140	AT5G05310
AT4G35270	AT5G05370
AT4G35335	AT5G05450
AT4G35410	AT5G05460
AT4G35500	AT5G05480
AT4G35560	AT5G05540
AT4G35740	AT5G05560
AT4G35785	AT5G05570
AT4G35790	AT5G05580
AT4G35800	AT5G05600
AT4G35830	AT5G05610
AT4G35850	AT5G05660
AT4G35860	AT5G05670
AT4G35870	AT5G05680
AT4G35880	AT5G05690
AT4G35890	AT5G05730
AT4G35910	AT5G05780
AT4G35920	AT5G05820
AT4G35970	AT5G05840
AT4G36050	AT5G05920
AT4G36052	AT5G05930
AT4G36080	AT5G05960
AT4G36090	AT5G05970
AT4G36140	AT5G05980
AT4G36180	AT5G05987
AT4G36190	AT5G06000
AT4G36195	AT5G06050
AT4G36210	AT5G06110
AT4G36290	AT5G06120
AT4G36360	AT5G06140
AT4G36390	AT5G06160
AT4G36400	AT5G06220
AT4G36440	AT5G06265
AT4G36480	AT5G06350
AT4G36520	AT5G06370
AT4G36630	AT5G06390
AT4G36650	AT5G06400
AT4G36690	AT5G06410
AT4G36720	AT5G06440
AT4G36730	AT5G06460
AT4G36760	AT5G06470
AT4G36770	AT5G06540
AT4G36800	AT5G06600
AT4G36860	AT5G06680
AT4G36940	AT5G06780
AT4G36960	AT5G06810
AT4G36980	AT5G06820
AT4G37090	AT5G06830
AT4G37095	AT5G06905
AT4G37100	AT5G06910
AT4G37120	AT5G06930
AT4G37130	AT5G06950
AT4G37190	AT5G06960
AT4G37270	AT5G06970
AT4G37280	AT5G07090
AT4G37440	AT5G07120
AT4G37460	AT5G07130
AT4G37480	AT5G07140
AT4G37483	AT5G07270
AT4G37550	AT5G07290
AT4G37553	AT5G07350
AT4G37560	AT5G07730
AT4G37640	AT5G07740
AT4G37685	AT5G07830
AT4G37820	AT5G07910
AT4G37830	AT5G07920
AT4G37880	AT5G07940
AT4G37910	AT5G07960
AT4G38080	AT5G07970
AT4G38120	AT5G07980
AT4G38130	AT5G08060
AT4G38200	AT5G08080
AT4G38230	AT5G08130
AT4G38240	AT5G08160
AT4G38270	AT5G08180
AT4G38290	AT5G08190
AT4G38320	AT5G08200
AT4G38350	AT5G08230
AT4G38360	AT5G08280
AT4G38430	AT5G08290
AT4G38440	AT5G08300
AT4G38470	AT5G08305
AT4G38480	AT5G08380
AT4G38510	AT5G08390
AT4G38520	AT5G08400
AT4G38600	AT5G08415
AT4G38760	AT5G08420
AT4G38790	AT5G08430
AT4G38890	AT5G08440
AT4G38920	AT5G08470
AT4G38930	AT5G08520
AT4G38980	AT5G08530
AT4G39050	AT5G08550
AT4G39080	AT5G08560

AT4G39100	AT5G08570
AT4G39110	AT5G08590
AT4G39120	AT5G08610
AT4G39140	AT5G08620
AT4G39150	AT5G08630
AT4G39160	AT5G08660
AT4G39170	AT5G08670
AT4G39210	AT5G08680
AT4G39220	AT5G08690
AT4G39280	AT5G08710
AT4G39350	AT5G08740
AT4G39370	AT5G09290
AT4G39390	AT5G09300
AT4G39420	AT5G09320
AT4G39520	AT5G09330
AT4G39680	AT5G09350
AT4G39690	AT5G09380
AT4G39850	AT5G09390
AT4G39860	AT5G09400
AT4G39890	AT5G09410
AT4G39900	AT5G09590
AT4G39910	AT5G09680
AT4G39960	AT5G09730
AT4G40000	AT5G09740
AT4G40050	AT5G09770
AT5G01230	AT5G09810
AT5G01270	AT5G09850
AT5G01290	AT5G09860
AT5G01400	AT5G09870
AT5G01460	AT5G09880
AT5G01510	AT5G09890
AT5G01595	AT5G09900
AT5G01600	AT5G10010
AT5G01715	AT5G10020
AT5G01770	AT5G10050
AT5G01800	AT5G10060
AT5G01810	AT5G10200
AT5G01930	AT5G10240
AT5G01950	AT5G10270
AT5G02010	AT5G10290
AT5G02040	AT5G10340
AT5G02070	AT5G10350
AT5G02130	AT5G10360
AT5G02150	AT5G10450
AT5G02290	AT5G10460
AT5G02310	AT5G10470
AT5G02320	AT5G10480
AT5G02410	AT5G10490
AT5G02450	AT5G10510
AT5G02470	AT5G10540
AT5G02480	AT5G10560
AT5G02502	AT5G10630
AT5G02680	AT5G10710
AT5G02690	AT5G10720
AT5G02720	AT5G10730
AT5G02740	AT5G10780
AT5G02800	AT5G10790
AT5G02840	AT5G10800
AT5G02860	AT5G10810
AT5G02880	AT5G10820
AT5G02960	AT5G10840
AT5G03040	AT5G10860
AT5G03070	AT5G10920
AT5G03160	AT5G10940
AT5G03280	AT5G11010
AT5G03290	AT5G11030
AT5G03300	AT5G11040
AT5G03320	AT5G11110
AT5G03330	AT5G11180
AT5G03340	AT5G11200
AT5G03415	AT5G11240
AT5G03420	AT5G11250
AT5G03430	AT5G11280
AT5G03440	AT5G11340
AT5G03450	AT5G11350
AT5G03455	AT5G11380
AT5G03460	AT5G11410
AT5G03500	AT5G11430
AT5G03520	AT5G11470
AT5G03540	AT5G11480
AT5G03630	AT5G11490
AT5G03650	AT5G11500
AT5G03660	AT5G11520
AT5G03690	AT5G11560
AT5G03730	AT5G11650
AT5G03740	AT5G11670
AT5G03780	AT5G11680
AT5G03830	AT5G11700
AT5G03900	AT5G11710
AT5G03930	AT5G11750
AT5G03940	AT5G11770
AT5G03970	AT5G11790
AT5G04040	AT5G11800
AT5G04050	AT5G11850
AT5G04060	AT5G11860
AT5G04090	AT5G11880
AT5G04110	AT5G11900
AT5G04130	AT5G11960
AT5G04235	AT5G11980
AT5G04240	AT5G12040
AT5G04280	AT5G12080
AT5G04290	AT5G12120
AT5G04320	AT5G12130
AT5G04420	AT5G12150
AT5G04430	AT5G12200
AT5G04460	AT5G12210
AT5G04480	AT5G12220
AT5G04510	AT5G12240
AT5G04540	AT5G12250
AT5G04550	AT5G12260
AT5G04560	AT5G12290
AT5G04590	AT5G12310
AT5G04670	AT5G12350
AT5G04740	AT5G12360
AT5G04750	AT5G12370
AT5G04800	AT5G12400
AT5G04885	AT5G12430
AT5G04895	AT5G12440
AT5G04910	AT5G12470
AT5G04920	AT5G12480
AT5G04930	AT5G12840
AT5G04940	AT5G12850
AT5G05000	AT5G12860

AT5G05010	AT5G12970
AT5G05080	AT5G12980
AT5G05100	AT5G13000
AT5G05170	AT5G13010
AT5G05210	AT5G13020
AT5G05310	AT5G13030
AT5G05450	AT5G13070
AT5G05520	AT5G13110
AT5G05560	AT5G13160
AT5G05570	AT5G13190
AT5G05580	AT5G13230
AT5G05610	AT5G13240
AT5G05670	AT5G13260
AT5G05680	AT5G13270
AT5G05700	AT5G13280
AT5G05780	AT5G13290
AT5G05800	AT5G13300
AT5G05930	AT5G13360
AT5G05960	AT5G13390
AT5G05970	AT5G13400
AT5G05980	AT5G13410
AT5G06060	AT5G13420
AT5G06100	AT5G13430
AT5G06110	AT5G13440
AT5G06120	AT5G13450
AT5G06140	AT5G13460
AT5G06160	AT5G13480
AT5G06180	AT5G13490
AT5G06220	AT5G13530
AT5G06240	AT5G13550
AT5G06260	AT5G13560
AT5G06265	AT5G13570
AT5G06350	AT5G13590
AT5G06440	AT5G13610
AT5G06460	AT5G13640
AT5G06560	AT5G13650
AT5G06600	AT5G13680
AT5G06670	AT5G13690
AT5G06680	AT5G13710
AT5G06770	AT5G13740
AT5G06780	AT5G13760
AT5G06830	AT5G13770
AT5G06950	AT5G13780
AT5G06960	AT5G13820
AT5G06970	AT5G13840
AT5G07020	AT5G13850
AT5G07070	AT5G13950
AT5G07090	AT5G13960
AT5G07130	AT5G13980
AT5G07270	AT5G13990
AT5G07290	AT5G14030
AT5G07300	AT5G14040
AT5G07340	AT5G14060
AT5G07350	AT5G14150
AT5G07580	AT5G14160
AT5G07590	AT5G14170
AT5G07610	AT5G14210
AT5G07630	AT5G14230
AT5G07740	AT5G14240
AT5G07890	AT5G14250
AT5G07910	AT5G14260
AT5G07920	AT5G14270
AT5G07940	AT5G14310
AT5G07970	AT5G14390
AT5G07980	AT5G14520
AT5G08010	AT5G14540
AT5G08080	AT5G14580
AT5G08100	AT5G14590
AT5G08110	AT5G14600
AT5G08120	AT5G14610
AT5G08160	AT5G14640
AT5G08170	AT5G14710
AT5G08180	AT5G14720
AT5G08190	AT5G14760
AT5G08200	AT5G14790
AT5G08210	AT5G14800
AT5G08220	AT5G14850
AT5G08230	AT5G14980
AT5G08270	AT5G15020
AT5G08280	AT5G15070
AT5G08290	AT5G15080
AT5G08300	AT5G15090
AT5G08320	AT5G15120
AT5G08340	AT5G15200
AT5G08390	AT5G15300
AT5G08420	AT5G15320
AT5G08440	AT5G15390
AT5G08450	AT5G15400
AT5G08470	AT5G15440
AT5G08530	AT5G15450
AT5G08560	AT5G15470
AT5G08570	AT5G15530
AT5G08590	AT5G15540
AT5G08630	AT5G15550
AT5G08650	AT5G15610
AT5G08660	AT5G15650
AT5G08670	AT5G15680
AT5G08680	AT5G15700
AT5G08690	AT5G15720
AT5G08720	AT5G15730
AT5G08740	AT5G15750
AT5G08780	AT5G15810
AT5G09225	AT5G15860
AT5G09230	AT5G15890
AT5G09270	AT5G16070
AT5G09290	AT5G16130
AT5G09300	AT5G16210
AT5G09330	AT5G16260
AT5G09350	AT5G16270
AT5G09380	AT5G16280
AT5G09390	AT5G16290
AT5G09400	AT5G16300
AT5G09410	AT5G16390
AT5G09420	AT5G16420
AT5G09590	AT5G16440
AT5G09650	AT5G16520
AT5G09670	AT5G16620
AT5G09672	AT5G16630
AT5G09680	AT5G16640
AT5G09740	AT5G16660
AT5G09770	AT5G16680
AT5G09810	AT5G16690
AT5G09870	AT5G16710

AT5G09880	AT5G16750
AT5G09900	AT5G16780
AT5G09920	AT5G16830
AT5G09990	AT5G16840
AT5G10010	AT5G16850
AT5G10020	AT5G16880
AT5G10060	AT5G16890
AT5G10070	AT5G16910
AT5G10190	AT5G16930
AT5G10200	AT5G17020
AT5G10240	AT5G17070
AT5G10270	AT5G17210
AT5G10290	AT5G17240
AT5G10350	AT5G17250
AT5G10360	AT5G17270
AT5G10460	AT5G17290
AT5G10470	AT5G17410
AT5G10480	AT5G17420
AT5G10490	AT5G17430
AT5G10560	AT5G17440
AT5G10630	AT5G17520
AT5G10680	AT5G17530
AT5G10710	AT5G17620
AT5G10720	AT5G17680
AT5G10730	AT5G17770
AT5G10780	AT5G17790
AT5G10800	AT5G17840
AT5G10810	AT5G17890
AT5G10840	AT5G17920
AT5G10910	AT5G17930
AT5G10940	AT5G18040
AT5G10950	AT5G18070
AT5G11010	AT5G18100
AT5G11030	AT5G18110
AT5G11040	AT5G18120
AT5G11170	AT5G18180
AT5G11200	AT5G18190
AT5G11240	AT5G18230
AT5G11242	AT5G18280
AT5G11250	AT5G18362
AT5G11270	AT5G18400
AT5G11340	AT5G18410
AT5G11380	AT5G18420
AT5G11390	AT5G18440
AT5G11430	AT5G18480
AT5G11470	AT5G18525
AT5G11490	AT5G18580
AT5G11500	AT5G18590
AT5G11520	AT5G18610
AT5G11530	AT5G18620
AT5G11560	AT5G18630
AT5G11580	AT5G18640
AT5G11640	AT5G18650
AT5G11670	AT5G18680
AT5G11680	AT5G18830
AT5G11700	AT5G18890
AT5G11710	AT5G18900
AT5G11730	AT5G19000
AT5G11760	AT5G19020
AT5G11790	AT5G19030
AT5G11800	AT5G19130
AT5G11810	AT5G19160
AT5G11850	AT5G19180
AT5G11900	AT5G19280
AT5G11910	AT5G19300
AT5G11960	AT5G19320
AT5G11980	AT5G19330
AT5G12080	AT5G19360
AT5G12120	AT5G19370
AT5G12130	AT5G19380
AT5G12150	AT5G19390
AT5G12200	AT5G19400
AT5G12210	AT5G19410
AT5G12220	AT5G19420
AT5G12230	AT5G19430
AT5G12240	AT5G19450
AT5G12250	AT5G19480
AT5G12290	AT5G19485
AT5G12310	AT5G19500
AT5G12350	AT5G19550
AT5G12370	AT5G19590
AT5G12380	AT5G19600
AT5G12400	AT5G19620
AT5G12410	AT5G19630
AT5G12430	AT5G19660
AT5G12480	AT5G19690
AT5G12840	AT5G19750
AT5G12850	AT5G19760
AT5G12970	AT5G19790
AT5G12980	AT5G19820
AT5G13000	AT5G19860
AT5G13010	AT5G19910
AT5G13020	AT5G19920
AT5G13030	AT5G19990
AT5G13070	AT5G20010
AT5G13110	AT5G20020
AT5G13160	AT5G20060
AT5G13240	AT5G20070
AT5G13260	AT5G20080
AT5G13300	AT5G20090
AT5G13310	AT5G20120
AT5G13340	AT5G20165
AT5G13360	AT5G20200
AT5G13420	AT5G20280
AT5G13430	AT5G20290
AT5G13440	AT5G20320
AT5G13450	AT5G20350
AT5G13460	AT5G20380
AT5G13480	AT5G20490
AT5G13490	AT5G20510
AT5G13500	AT5G20520
AT5G13530	AT5G20570
AT5G13550	AT5G20580
AT5G13560	AT5G20590
AT5G13590	AT5G20600
AT5G13650	AT5G20610
AT5G13680	AT5G20620
AT5G13710	AT5G20680
AT5G13800	AT5G20720
AT5G13850	AT5G20730
AT5G13950	AT5G20830
AT5G13980	AT5G20840
AT5G13990	AT5G20890

AT5G14030	AT5G20900
AT5G14040	AT5G20920
AT5G14060	AT5G20930
AT5G14090	AT5G20950
AT5G14140	AT5G20960
AT5G14170	AT5G20990
AT5G14220	AT5G21010
AT5G14240	AT5G21040
AT5G14250	AT5G21060
AT5G14270	AT5G21070
AT5G14390	AT5G21080
AT5G14420	AT5G21090
AT5G14430	AT5G21140
AT5G14440	AT5G21160
AT5G14480	AT5G21170
AT5G14500	AT5G21274
AT5G14520	AT5G21970
AT5G14540	AT5G21990
AT5G14580	AT5G22000
AT5G14590	AT5G22010
AT5G14600	AT5G22030
AT5G14610	AT5G22060
AT5G14640	AT5G22080
AT5G14710	AT5G22100
AT5G14720	AT5G22120
AT5G14790	AT5G22280
AT5G14850	AT5G22320
AT5G14950	AT5G22330
AT5G15020	AT5G22350
AT5G15070	AT5G22355
AT5G15080	AT5G22400
AT5G15090	AT5G22440
AT5G15170	AT5G22450
AT5G15220	AT5G22480
AT5G15270	AT5G22510
AT5G15280	AT5G22620
AT5G15400	AT5G22640
AT5G15440	AT5G22650
AT5G15450	AT5G22700
AT5G15470	AT5G22750
AT5G15540	AT5G22760
AT5G15550	AT5G22770
AT5G15570	AT5G22780
AT5G15610	AT5G22790
AT5G15640	AT5G22820
AT5G15650	AT5G22840
AT5G15680	AT5G22950
AT5G15700	AT5G23050
AT5G15720	AT5G23080
AT5G15730	AT5G23110
AT5G15740	AT5G23150
AT5G15810	AT5G23290
AT5G15840	AT5G23340
AT5G15890	AT5G23380
AT5G15920	AT5G23390
AT5G15930	AT5G23430
AT5G16040	AT5G23450
AT5G16050	AT5G23490
AT5G16070	AT5G23510
AT5G16120	AT5G23535
AT5G16130	AT5G23550
AT5G16150	AT5G23575
AT5G16210	AT5G23580
AT5G16260	AT5G23610
AT5G16270	AT5G23630
AT5G16280	AT5G23670
AT5G16290	AT5G23700
AT5G16300	AT5G23720
AT5G16310	AT5G23740
AT5G16390	AT5G23880
AT5G16440	AT5G23890
AT5G16505	AT5G23900
AT5G16510	AT5G24060
AT5G16520	AT5G24320
AT5G16610	AT5G24340
AT5G16620	AT5G24350
AT5G16630	AT5G24430
AT5G16650	AT5G24450
AT5G16660	AT5G24470
AT5G16680	AT5G24490
AT5G16715	AT5G24590
AT5G16730	AT5G24620
AT5G16750	AT5G24650
AT5G16780	AT5G24670
AT5G16810	AT5G24680
AT5G16820	AT5G24690
AT5G16830	AT5G24710
AT5G16840	AT5G24740
AT5G16880	AT5G24840
AT5G16890	AT5G24970
AT5G16910	AT5G25060
AT5G16940	AT5G25100
AT5G17010	AT5G25150
AT5G17020	AT5G25265
AT5G17060	AT5G25270
AT5G17070	AT5G25340
AT5G17250	AT5G25360
AT5G17270	AT5G25475
AT5G17290	AT5G25520
AT5G17300	AT5G25540
AT5G17410	AT5G25560
AT5G17440	AT5G25590
AT5G17520	AT5G25750
AT5G17530	AT5G25752
AT5G17620	AT5G25780
AT5G17680	AT5G25900
AT5G17710	AT5G26030
AT5G17770	AT5G26040
AT5G17790	AT5G26110
AT5G17890	AT5G26180
AT5G17910	AT5G26210
AT5G17920	AT5G26240
AT5G17930	AT5G26360
AT5G18040	AT5G26570
AT5G18065	AT5G26610
AT5G18110	AT5G26667
AT5G18120	AT5G26680
AT5G18180	AT5G26700
AT5G18190	AT5G26710
AT5G18230	AT5G26742
AT5G18245	AT5G26751
AT5G18280	AT5G26780
AT5G18360	AT5G26800

AT5G18362	AT5G26830
AT5G18400	AT5G26850
AT5G18410	AT5G26860
AT5G18420	AT5G26880
AT5G18480	AT5G26910
AT5G18500	AT5G27020
AT5G18525	AT5G27030
AT5G18540	AT5G27120
AT5G18550	AT5G27200
AT5G18580	AT5G27210
AT5G18610	AT5G27270
AT5G18620	AT5G27380
AT5G18630	AT5G27395
AT5G18640	AT5G27430
AT5G18650	AT5G27470
AT5G18680	AT5G27540
AT5G18750	AT5G27550
AT5G18760	AT5G27600
AT5G18830	AT5G27620
AT5G18900	AT5G27640
AT5G18960	AT5G27700
AT5G19000	AT5G27720
AT5G19010	AT5G27740
AT5G19020	AT5G27770
AT5G19050	AT5G27820
AT5G19070	AT5G27850
AT5G19130	AT5G27860
AT5G19180	AT5G27950
AT5G19220	AT5G27970
AT5G19221	AT5G27990
AT5G19280	AT5G28050
AT5G19320	AT5G28060
AT5G19330	AT5G28220
AT5G19350	AT5G28290
AT5G19370	AT5G28350
AT5G19390	AT5G28740
AT5G19400	AT5G28750
AT5G19420	AT5G28830
AT5G19430	AT5G28850
AT5G19450	AT5G28900
AT5G19510	AT5G30490
AT5G19550	AT5G30495
AT5G19660	AT5G30510
AT5G19680	AT5G32460
AT5G19690	AT5G32470
AT5G19750	AT5G33320
AT5G19760	AT5G34850
AT5G19770	AT5G34940
AT5G19780	AT5G35080
AT5G19820	AT5G35160
AT5G19840	AT5G35180
AT5G19960	AT5G35200
AT5G19990	AT5G35210
AT5G20020	AT5G35320
AT5G20040	AT5G35360
AT5G20060	AT5G35400
AT5G20080	AT5G35430
AT5G20090	AT5G35460
AT5G20130	AT5G35530
AT5G20165	AT5G35560
AT5G20170	AT5G35570
AT5G20200	AT5G35590
AT5G20280	AT5G35620
AT5G20290	AT5G35640
AT5G20300	AT5G35690
AT5G20320	AT5G35700
AT5G20350	AT5G35730
AT5G20380	AT5G35910
AT5G20450	AT5G35930
AT5G20480	AT5G35970
AT5G20490	AT5G35980
AT5G20500	AT5G36210
AT5G20510	AT5G36230
AT5G20520	AT5G36290
AT5G20580	AT5G36880
AT5G20600	AT5G36940
AT5G20610	AT5G36950
AT5G20620	AT5G37020
AT5G20660	AT5G37130
AT5G20680	AT5G37310
AT5G20720	AT5G37340
AT5G20730	AT5G37350
AT5G20840	AT5G37360
AT5G20890	AT5G37370
AT5G20920	AT5G37380
AT5G20950	AT5G37475
AT5G20960	AT5G37510
AT5G20970	AT5G37570
AT5G20980	AT5G37720
AT5G20990	AT5G37780
AT5G21010	AT5G37850
AT5G21040	AT5G37930
AT5G21060	AT5G38280
AT5G21070	AT5G38470
AT5G21090	AT5G38480
AT5G21160	AT5G38510
AT5G21170	AT5G38560
AT5G21274	AT5G38600
AT5G21326	AT5G38630
AT5G21910	AT5G38660
AT5G21930	AT5G38720
AT5G21970	AT5G38830
AT5G21990	AT5G38840
AT5G22000	AT5G38880
AT5G22010	AT5G39340
AT5G22030	AT5G39350
AT5G22040	AT5G39500
AT5G22060	AT5G39510
AT5G22080	AT5G39520
AT5G22100	AT5G39570
AT5G22110	AT5G39590
AT5G22120	AT5G39600
AT5G22130	AT5G39680
AT5G22220	AT5G39740
AT5G22280	AT5G39990
AT5G22320	AT5G40200
AT5G22330	AT5G40280
AT5G22340	AT5G40300
AT5G22350	AT5G40410
AT5G22370	AT5G40450
AT5G22440	AT5G40480
AT5G22450	AT5G40490
AT5G22480	AT5G40530

AT5G22510	AT5G40580
AT5G22640	AT5G40595
AT5G22650	AT5G40650
AT5G22700	AT5G40670
AT5G22730	AT5G40740
AT5G22760	AT5G40760
AT5G22770	AT5G40770
AT5G22780	AT5G40810
AT5G22820	AT5G40850
AT5G22850	AT5G40870
AT5G22950	AT5G40880
AT5G23040	AT5G40930
AT5G23050	AT5G41010
AT5G23080	AT5G41100
AT5G23110	AT5G41180
AT5G23140	AT5G41260
AT5G23150	AT5G41320
AT5G23200	AT5G41340
AT5G23250	AT5G41360
AT5G23300	AT5G41370
AT5G23310	AT5G41580
AT5G23320	AT5G41620
AT5G23330	AT5G41650
AT5G23350	AT5G41680
AT5G23380	AT5G41770
AT5G23390	AT5G41940
AT5G23395	AT5G41950
AT5G23430	AT5G41970
AT5G23450	AT5G42080
AT5G23520	AT5G42100
AT5G23535	AT5G42140
AT5G23540	AT5G42190
AT5G23550	AT5G42220
AT5G23570	AT5G42270
AT5G23575	AT5G42310
AT5G23590	AT5G42320
AT5G23630	AT5G42390
AT5G23670	AT5G42400
AT5G23720	AT5G42450
AT5G23740	AT5G42470
AT5G23880	AT5G42480
AT5G23890	AT5G42490
AT5G23900	AT5G42520
AT5G24060	AT5G42540
AT5G24120	AT5G42560
AT5G24170	AT5G42570
AT5G24260	AT5G42620
AT5G24280	AT5G42740
AT5G24300	AT5G42790
AT5G24310	AT5G42870
AT5G24316	AT5G42940
AT5G24320	AT5G42950
AT5G24340	AT5G42960
AT5G24350	AT5G42965
AT5G24360	AT5G42970
AT5G24430	AT5G42980
AT5G24450	AT5G43010
AT5G24470	AT5G43060
AT5G24590	AT5G43100
AT5G24620	AT5G43130
AT5G24670	AT5G43140
AT5G24690	AT5G43270
AT5G24710	AT5G43310
AT5G24740	AT5G43320
AT5G24750	AT5G43430
AT5G24810	AT5G43460
AT5G24840	AT5G43560
AT5G24970	AT5G43720
AT5G25060	AT5G43740
AT5G25080	AT5G43745
AT5G25090	AT5G43790
AT5G25100	AT5G43810
AT5G25150	AT5G43830
AT5G25170	AT5G43870
AT5G25270	AT5G43900
AT5G25360	AT5G43930
AT5G25475	AT5G43940
AT5G25480	AT5G43960
AT5G25510	AT5G43990
AT5G25520	AT5G44000
AT5G25540	AT5G44090
AT5G25560	AT5G44180
AT5G25580	AT5G44200
AT5G25640	AT5G44230
AT5G25750	AT5G44240
AT5G25757	AT5G44250
AT5G25780	AT5G44320
AT5G25800	AT5G44330
AT5G25900	AT5G44340
AT5G26030	AT5G44500
AT5G26038	AT5G44510
AT5G26110	AT5G44560
AT5G26160	AT5G44635
AT5G26170	AT5G44710
AT5G26180	AT5G44730
AT5G26200	AT5G44750
AT5G26210	AT5G44790
AT5G26240	AT5G44800
AT5G26360	AT5G45010
AT5G26570	AT5G45030
AT5G26610	AT5G45130
AT5G26667	AT5G45140
AT5G26680	AT5G45160
AT5G26690	AT5G45190
AT5G26700	AT5G45275
AT5G26710	AT5G45290
AT5G26740	AT5G45300
AT5G26742	AT5G45410
AT5G26751	AT5G45420
AT5G26780	AT5G45430
AT5G26800	AT5G45440
AT5G26820	AT5G45510
AT5G26830	AT5G45550
AT5G26850	AT5G45560
AT5G26860	AT5G45620
AT5G26910	AT5G45740
AT5G26940	AT5G45750
AT5G26980	AT5G45775
AT5G26990	AT5G45900
AT5G27030	AT5G46020
AT5G27100	AT5G46040
AT5G27120	AT5G46070
AT5G27240	AT5G46150

AT5G27270	AT5G46210
AT5G27320	AT5G46250
AT5G27380	AT5G46290
AT5G27395	AT5G46400
AT5G27400	AT5G46420
AT5G27410	AT5G46430
AT5G27430	AT5G46470
AT5G27450	AT5G46550
AT5G27470	AT5G46620
AT5G27490	AT5G46630
AT5G27520	AT5G46640
AT5G27540	AT5G46680
AT5G27600	AT5G46750
AT5G27620	AT5G46840
AT5G27640	AT5G46910
AT5G27680	AT5G47010
AT5G27700	AT5G47020
AT5G27720	AT5G47030
AT5G27740	AT5G47040
AT5G27760	AT5G47080
AT5G27770	AT5G47100
AT5G27771	AT5G47120
AT5G27820	AT5G47200
AT5G27830	AT5G47210
AT5G27840	AT5G47310
AT5G27860	AT5G47320
AT5G27970	AT5G47400
AT5G27980	AT5G47430
AT5G28050	AT5G47455
AT5G28060	AT5G47460
AT5G28130	AT5G47480
AT5G28220	AT5G47490
AT5G28290	AT5G47520
AT5G28350	AT5G47540
AT5G28390	AT5G47570
AT5G28530	AT5G47660
AT5G28540	AT5G47670
AT5G28622	AT5G47690
AT5G28740	AT5G47700
AT5G28840	AT5G47710
AT5G28850	AT5G47730
AT5G28900	AT5G47750
AT5G29000	AT5G47780
AT5G30510	AT5G47820
AT5G32440	AT5G47840
AT5G32450	AT5G47860
AT5G32470	AT5G47880
AT5G33257	AT5G47890
AT5G33280	AT5G47970
AT5G33320	AT5G48020
AT5G34834	AT5G48030
AT5G34850	AT5G48120
AT5G34853	AT5G48240
AT5G34940	AT5G48385
AT5G35080	AT5G48440
AT5G35160	AT5G48520
AT5G35180	AT5G48530
AT5G35200	AT5G48545
AT5G35210	AT5G48580
AT5G35330	AT5G48610
AT5G35360	AT5G48640
AT5G35400	AT5G48650
AT5G35410	AT5G48655
AT5G35430	AT5G48720
AT5G35460	AT5G48760
AT5G35530	AT5G48810
AT5G35560	AT5G48830
AT5G35570	AT5G48940
AT5G35590	AT5G48960
AT5G35620	AT5G48970
AT5G35640	AT5G49010
AT5G35650	AT5G49020
AT5G35670	AT5G49030
AT5G35690	AT5G49210
AT5G35700	AT5G49220
AT5G35730	AT5G49400
AT5G35738	AT5G49430
AT5G35740	AT5G49460
AT5G35750	AT5G49470
AT5G35753	AT5G49500
AT5G35840	AT5G49555
AT5G35910	AT5G49570
AT5G35930	AT5G49580
AT5G35980	AT5G49650
AT5G35995	AT5G49720
AT5G36210	AT5G49810
AT5G36230	AT5G49830
AT5G36290	AT5G49840
AT5G36880	AT5G49880
AT5G36890	AT5G49890
AT5G36930	AT5G49920
AT5G36940	AT5G49930
AT5G37020	AT5G49940
AT5G37050	AT5G49950
AT5G37130	AT5G49970
AT5G37190	AT5G49980
AT5G37310	AT5G49990
AT5G37340	AT5G50000
AT5G37350	AT5G50170
AT5G37370	AT5G50310
AT5G37380	AT5G50320
AT5G37475	AT5G50330
AT5G37480	AT5G50340
AT5G37510	AT5G50380
AT5G37530	AT5G50400
AT5G37590	AT5G50410
AT5G37680	AT5G50420
AT5G37720	AT5G50430
AT5G37780	AT5G50460
AT5G37830	AT5G50780
AT5G37850	AT5G50850
AT5G37930	AT5G50860
AT5G38070	AT5G50920
AT5G38140	AT5G50960
AT5G38220	AT5G51060
AT5G38280	AT5G51070
AT5G38380	AT5G51080
AT5G38470	AT5G51100
AT5G38480	AT5G51130
AT5G38510	AT5G51140
AT5G38560	AT5G51150
AT5G38600	AT5G51200
AT5G38640	AT5G51220

AT5G38690	AT5G51230
AT5G38720	AT5G51280
AT5G38830	AT5G51290
AT5G38840	AT5G51300
AT5G38860	AT5G51340
AT5G38870	AT5G51350
AT5G38880	AT5G51410
AT5G38895	AT5G51420
AT5G39040	AT5G51430
AT5G39350	AT5G51510
AT5G39380	AT5G51540
AT5G39500	AT5G51560
AT5G39510	AT5G51570
AT5G39590	AT5G51590
AT5G39600	AT5G51660
AT5G39660	AT5G51710
AT5G39740	AT5G51820
AT5G39830	AT5G51830
AT5G39840	AT5G51840
AT5G39900	AT5G51940
AT5G39960	AT5G51970
AT5G40100	AT5G51980
AT5G40180	AT5G52040
AT5G40200	AT5G52070
AT5G40240	AT5G52100
AT5G40270	AT5G52180
AT5G40280	AT5G52200
AT5G40300	AT5G52210
AT5G40340	AT5G52240
AT5G40440	AT5G52290
AT5G40480	AT5G52380
AT5G40490	AT5G52470
AT5G40530	AT5G52530
AT5G40550	AT5G52580
AT5G40580	AT5G52600
AT5G40600	AT5G52630
AT5G40670	AT5G52650
AT5G40700	AT5G52660
AT5G40740	AT5G52820
AT5G40760	AT5G52840
AT5G40810	AT5G52850
AT5G40820	AT5G52920
AT5G40840	AT5G52960
AT5G40850	AT5G53000
AT5G40870	AT5G53010
AT5G40910	AT5G53045
AT5G40920	AT5G53050
AT5G40980	AT5G53070
AT5G41060	AT5G53090
AT5G41100	AT5G53120
AT5G41109	AT5G53130
AT5G41140	AT5G53140
AT5G41150	AT5G53150
AT5G41180	AT5G53170
AT5G41190	AT5G53180
AT5G41210	AT5G53280
AT5G41220	AT5G53300
AT5G41240	AT5G53320
AT5G41260	AT5G53350
AT5G41320	AT5G53360
AT5G41330	AT5G53400
AT5G41340	AT5G53430
AT5G41350	AT5G53440
AT5G41360	AT5G53450
AT5G41370	AT5G53460
AT5G41480	AT5G53480
AT5G41580	AT5G53520
AT5G41760	AT5G53530
AT5G41770	AT5G53570
AT5G41790	AT5G53620
AT5G41940	AT5G53650
AT5G41950	AT5G53760
AT5G41970	AT5G53770
AT5G41990	AT5G53860
AT5G41992	AT5G53890
AT5G42020	AT5G53895
AT5G42080	AT5G53900
AT5G42140	AT5G53950
AT5G42150	AT5G54080
AT5G42220	AT5G54090
AT5G42240	AT5G54170
AT5G42320	AT5G54200
AT5G42390	AT5G54260
AT5G42400	AT5G54270
AT5G42470	AT5G54280
AT5G42520	AT5G54310
AT5G42540	AT5G54430
AT5G42560	AT5G54440
AT5G42568	AT5G54540
AT5G42620	AT5G54580
AT5G42660	AT5G54590
AT5G42740	AT5G54600
AT5G42760	AT5G54670
AT5G42770	AT5G54680
AT5G42810	AT5G54750
AT5G42820	AT5G54760
AT5G42870	AT5G54800
AT5G42940	AT5G54810
AT5G42950	AT5G54830
AT5G42965	AT5G54855
AT5G42970	AT5G54870
AT5G42980	AT5G54900
AT5G42990	AT5G54910
AT5G43010	AT5G54930
AT5G43060	AT5G55040
AT5G43100	AT5G55060
AT5G43130	AT5G55070
AT5G43140	AT5G55100
AT5G43160	AT5G55120
AT5G43270	AT5G55125
AT5G43310	AT5G55130
AT5G43320	AT5G55190
AT5G43460	AT5G55230
AT5G43560	AT5G55260
AT5G43670	AT5G55280
AT5G43710	AT5G55290
AT5G43720	AT5G55300
AT5G43820	AT5G55310
AT5G43822	AT5G55340
AT5G43830	AT5G55390
AT5G43850	AT5G55400
AT5G43880	AT5G55530
AT5G43900	AT5G55590

AT5G43920	AT5G55600
AT5G43930	AT5G55610
AT5G43940	AT5G55660
AT5G43960	AT5G55700
AT5G43990	AT5G55760
AT5G44070	AT5G55850
AT5G44090	AT5G55860
AT5G44100	AT5G55900
AT5G44150	AT5G55920
AT5G44180	AT5G56010
AT5G44200	AT5G56030
AT5G44240	AT5G56100
AT5G44250	AT5G56140
AT5G44270	AT5G56170
AT5G44340	AT5G56190
AT5G44370	AT5G56220
AT5G44450	AT5G56280
AT5G44510	AT5G56290
AT5G44560	AT5G56310
AT5G44562	AT5G56350
AT5G44660	AT5G56420
AT5G44710	AT5G56500
AT5G44720	AT5G56530
AT5G44750	AT5G56630
AT5G44790	AT5G56660
AT5G44800	AT5G56680
AT5G45030	AT5G56710
AT5G45050	AT5G56720
AT5G45130	AT5G56730
AT5G45140	AT5G56750
AT5G45160	AT5G56890
AT5G45190	AT5G56900
AT5G45275	AT5G56950
AT5G45300	AT5G57015
AT5G45330	AT5G57020
AT5G45360	AT5G57080
AT5G45410	AT5G57100
AT5G45428	AT5G57110
AT5G45430	AT5G57150
AT5G45475	AT5G57170
AT5G45480	AT5G57230
AT5G45500	AT5G57250
AT5G45510	AT5G57270
AT5G45550	AT5G57280
AT5G45560	AT5G57290
AT5G45590	AT5G57330
AT5G45600	AT5G57350
AT5G45610	AT5G57360
AT5G45620	AT5G57370
AT5G45710	AT5G57410
AT5G45750	AT5G57490
AT5G45780	AT5G57580
AT5G45900	AT5G57590
AT5G46020	AT5G57840
AT5G46070	AT5G57870
AT5G46090	AT5G57880
AT5G46150	AT5G57887
AT5G46180	AT5G57940
AT5G46190	AT5G57960
AT5G46210	AT5G57970
AT5G46280	AT5G57990
AT5G46290	AT5G58003
AT5G46340	AT5G58030
AT5G46400	AT5G58040
AT5G46410	AT5G58050
AT5G46420	AT5G58100
AT5G46430	AT5G58110
AT5G46450	AT5G58120
AT5G46470	AT5G58130
AT5G46550	AT5G58140
AT5G46560	AT5G58160
AT5G46570	AT5G58190
AT5G46630	AT5G58200
AT5G46750	AT5G58210
AT5G46800	AT5G58230
AT5G46910	AT5G58270
AT5G47010	AT5G58290
AT5G47020	AT5G58320
AT5G47030	AT5G58410
AT5G47040	AT5G58420
AT5G47080	AT5G58440
AT5G47090	AT5G58450
AT5G47120	AT5G58470
AT5G47140	AT5G58510
AT5G47180	AT5G58540
AT5G47200	AT5G58550
AT5G47210	AT5G58600
AT5G47320	AT5G58620
AT5G47420	AT5G58640
AT5G47430	AT5G58690
AT5G47480	AT5G58700
AT5G47490	AT5G58710
AT5G47520	AT5G58720
AT5G47580	AT5G58740
AT5G47670	AT5G58760
AT5G47690	AT5G58787
AT5G47720	AT5G58800
AT5G47750	AT5G58870
AT5G47780	AT5G58950
AT5G47820	AT5G58970
AT5G47840	AT5G58980
AT5G47860	AT5G59140
AT5G47880	AT5G59160
AT5G47940	AT5G59210
AT5G47970	AT5G59260
AT5G48020	AT5G59290
AT5G48030	AT5G59300
AT5G48120	AT5G59420
AT5G48150	AT5G59440
AT5G48160	AT5G59480
AT5G48230	AT5G59613
AT5G48250	AT5G59660
AT5G48335	AT5G59680
AT5G48380	AT5G59710
AT5G48385	AT5G59770
AT5G48520	AT5G59880
AT5G48600	AT5G59950
AT5G48610	AT5G59960
AT5G48655	AT5G60040
AT5G48680	AT5G60120
AT5G48720	AT5G60170
AT5G48790	AT5G60190
AT5G48830	AT5G60210

AT5G48960	AT5G60310
AT5G48965	AT5G60320
AT5G48970	AT5G60340
AT5G49020	AT5G60360
AT5G49030	AT5G60390
AT5G49160	AT5G60410
AT5G49220	AT5G60540
AT5G49380	AT5G60550
AT5G49390	AT5G60580
AT5G49400	AT5G60600
AT5G49430	AT5G60620
AT5G49460	AT5G60640
AT5G49470	AT5G60690
AT5G49510	AT5G60700
AT5G49530	AT5G60710
AT5G49540	AT5G60750
AT5G49555	AT5G60790
AT5G49570	AT5G60920
AT5G49580	AT5G60980
AT5G49600	AT5G60990
AT5G49640	AT5G61020
AT5G49720	AT5G61030
AT5G49810	AT5G61060
AT5G49830	AT5G61140
AT5G49880	AT5G61150
AT5G49910	AT5G61210
AT5G49930	AT5G61230
AT5G49980	AT5G61240
AT5G50000	AT5G61300
AT5G50020	AT5G61330
AT5G50110	AT5G61360
AT5G50170	AT5G61460
AT5G50230	AT5G61500
AT5G50310	AT5G61510
AT5G50315	AT5G61570
AT5G50320	AT5G61770
AT5G50340	AT5G61780
AT5G50370	AT5G61790
AT5G50380	AT5G61800
AT5G50400	AT5G61840
AT5G50430	AT5G61880
AT5G50780	AT5G61910
AT5G50840	AT5G61960
AT5G50850	AT5G61970
AT5G50920	AT5G62000
AT5G50960	AT5G62040
AT5G50990	AT5G62050
AT5G51050	AT5G62060
AT5G51070	AT5G62090
AT5G51080	AT5G62190
AT5G51150	AT5G62270
AT5G51200	AT5G62390
AT5G51230	AT5G62410
AT5G51290	AT5G62440
AT5G51340	AT5G62500
AT5G51390	AT5G62530
AT5G51400	AT5G62540
AT5G51410	AT5G62570
AT5G51430	AT5G62600
AT5G51450	AT5G62640
AT5G51460	AT5G62650
AT5G51510	AT5G62670
AT5G51540	AT5G62690
AT5G51560	AT5G62700
AT5G51620	AT5G62710
AT5G51630	AT5G62750
AT5G51640	AT5G62760
AT5G51660	AT5G62790
AT5G51680	AT5G62810
AT5G51690	AT5G62890
AT5G51700	AT5G62990
AT5G51710	AT5G63020
AT5G51820	AT5G63080
AT5G51830	AT5G63110
AT5G51840	AT5G63190
AT5G51890	AT5G63200
AT5G51970	AT5G63260
AT5G52040	AT5G63280
AT5G52070	AT5G63290
AT5G52100	AT5G63310
AT5G52200	AT5G63320
AT5G52210	AT5G63400
AT5G52230	AT5G63420
AT5G52280	AT5G63440
AT5G52430	AT5G63460
AT5G52470	AT5G63490
AT5G52471	AT5G63510
AT5G52520	AT5G63550
AT5G52530	AT5G63610
AT5G52540	AT5G63620
AT5G52545	AT5G63640
AT5G52550	AT5G63670
AT5G52552	AT5G63680
AT5G52560	AT5G63770
AT5G52580	AT5G63780
AT5G52600	AT5G63810
AT5G52650	AT5G63820
AT5G52660	AT5G63840
AT5G52790	AT5G63870
AT5G52820	AT5G63890
AT5G52880	AT5G63920
AT5G53000	AT5G63940
AT5G53010	AT5G63950
AT5G53048	AT5G63960
AT5G53060	AT5G63970
AT5G53070	AT5G63990
AT5G53080	AT5G64000
AT5G53120	AT5G64020
AT5G53140	AT5G64030
AT5G53150	AT5G64050
AT5G53170	AT5G64070
AT5G53180	AT5G64130
AT5G53300	AT5G64140
AT5G53310	AT5G64160
AT5G53330	AT5G64170
AT5G53340	AT5G64190
AT5G53350	AT5G64220
AT5G53430	AT5G64240
AT5G53440	AT5G64250
AT5G53450	AT5G64270
AT5G53460	AT5G64290
AT5G53480	AT5G64340
AT5G53530	AT5G64350

AT5G53570
AT5G53620
AT5G53760
AT5G53770
AT5G53850
AT5G53860
AT5G53890
AT5G53895
AT5G53930
AT5G53940
AT5G54080
AT5G54165
AT5G54170
AT5G54200
AT5G54260
AT5G54280
AT5G54310
AT5G54440
AT5G54520
AT5G54569
AT5G54570
AT5G54590
AT5G54600
AT5G54650
AT5G54670
AT5G54680
AT5G54730
AT5G54750
AT5G54760
AT5G54780
AT5G54800
AT5G54810
AT5G54830
AT5G54850
AT5G54855
AT5G54860
AT5G54870
AT5G54900
AT5G54930
AT5G55000
AT5G55040
AT5G55060
AT5G55070
AT5G55100
AT5G55130
AT5G55160
AT5G55190
AT5G55230
AT5G55300
AT5G55310
AT5G55390
AT5G55460
AT5G55480
AT5G55530
AT5G55600
AT5G55610
AT5G55660
AT5G55700
AT5G55760
AT5G55810
AT5G55820
AT5G55840
AT5G55850
AT5G55860
AT5G55896
AT5G55910
AT5G55920
AT5G55940
AT5G55990
AT5G56000
AT5G56020
AT5G56100
AT5G56130
AT5G56140
AT5G56180
AT5G56190
AT5G56210
AT5G56240
AT5G56250
AT5G56280
AT5G56290
AT5G56360
AT5G56420
AT5G56500
AT5G56530
AT5G56630
AT5G56730
AT5G56740
AT5G56890
AT5G56900
AT5G56930
AT5G56940
AT5G56950
AT5G57015
AT5G57020
AT5G57110
AT5G57120
AT5G57210
AT5G57230
AT5G57250
AT5G57280
AT5G57300
AT5G57330
AT5G57360
AT5G57410
AT5G57440
AT5G57460
AT5G57490
AT5G57580
AT5G57610
AT5G57655
AT5G57840
AT5G57860
AT5G57870
AT5G57880
AT5G57887
AT5G57930
AT5G57940
AT5G57990
AT5G58003
AT5G58030
AT5G58060
AT5G58100
AT5G64370
AT5G64390
AT5G64420
AT5G64440
AT5G64460
AT5G64500
AT5G64600
AT5G64610
AT5G64630
AT5G64670
AT5G64740
AT5G64760
AT5G64813
AT5G64840
AT5G64940
AT5G64950
AT5G64960
AT5G64970
AT5G65000
AT5G65010
AT5G65050
AT5G65060
AT5G65205
AT5G65240
AT5G65260
AT5G65270
AT5G65290
AT5G65430
AT5G65440
AT5G65460
AT5G65470
AT5G65490
AT5G65500
AT5G65540
AT5G65550
AT5G65620
AT5G65650
AT5G65670
AT5G65687
AT5G65720
AT5G65750
AT5G65760
AT5G65770
AT5G65810
AT5G65900
AT5G65910
AT5G65920
AT5G65930
AT5G65940
AT5G65950
AT5G65960
AT5G66010
AT5G66020
AT5G66030
AT5G66050
AT5G66060
AT5G66100
AT5G66130
AT5G66140
AT5G66180
AT5G66240
AT5G66280
AT5G66310
AT5G66410
AT5G66420
AT5G66500
AT5G66510
AT5G66530
AT5G66540
AT5G66610
AT5G66680
AT5G66730
AT5G66750
AT5G66760
AT5G66810
AT5G66860
AT5G66880
AT5G66950
AT5G67100
AT5G67110
AT5G67130
AT5G67170
AT5G67220
AT5G67230
AT5G67240
AT5G67250
AT5G67320
AT5G67380
AT5G67500
AT5G67530
AT5G67540
AT5G67560
AT5G67570
AT5G67590
AT5G67610
AT5G67630
AT5G67640

AT5G58130
AT5G58160
AT5G58190
AT5G58270
AT5G58350
AT5G58380
AT5G58410
AT5G58420
AT5G58450
AT5G58470
AT5G58510
AT5G58540
AT5G58550
AT5G58560
AT5G58570
AT5G58575
AT5G58620
AT5G58640
AT5G58690
AT5G58700
AT5G58710
AT5G58740
AT5G58760
AT5G58787
AT5G58800
AT5G58940
AT5G58950
AT5G59020
AT5G59160
AT5G59210
AT5G59300
AT5G59420
AT5G59440
AT5G59460
AT5G59480
AT5G59560
AT5G59610
AT5G59650
AT5G59710
AT5G59830
AT5G59840
AT5G59850
AT5G59880
AT5G59950
AT5G59960
AT5G59980
AT5G60020
AT5G60022
AT5G60040
AT5G60170
AT5G60190
AT5G60210
AT5G60310
AT5G60370
AT5G60390
AT5G60410
AT5G60548
AT5G60550
AT5G60570
AT5G60580
AT5G60590
AT5G60600
AT5G60620
AT5G60640
AT5G60690
AT5G60700
AT5G60710
AT5G60750
AT5G60820
AT5G60830
AT5G60920
AT5G60940
AT5G60980
AT5G60990
AT5G61060
AT5G61140
AT5G61150
AT5G61170
AT5G61190
AT5G61210
AT5G61220
AT5G61228
AT5G61230
AT5G61240
AT5G61300
AT5G61330
AT5G61380
AT5G61410
AT5G61450
AT5G61460
AT5G61500
AT5G61510
AT5G61530
AT5G61540
AT5G61580
AT5G61770
AT5G61780
AT5G61790
AT5G61830
AT5G61840
AT5G61900
AT5G61910
AT5G61960
AT5G61970
AT5G61980
AT5G61990
AT5G62000
AT5G62030
AT5G62050
AT5G62090
AT5G62130
AT5G62190
AT5G62300
AT5G62410
AT5G62460
AT5G62530
AT5G62540
AT5G62570
AT5G62600
AT5G62640
AT5G62650
AT5G62670
AT5G62690

AT5G62700
AT5G62710
AT5G62760
AT5G62790
AT5G62810
AT5G62880
AT5G62890
AT5G62930
AT5G62950
AT5G62998
AT5G63020
AT5G63040
AT5G63050
AT5G63080
AT5G63135
AT5G63190
AT5G63195
AT5G63200
AT5G63220
AT5G63260
AT5G63280
AT5G63320
AT5G63400
AT5G63420
AT5G63440
AT5G63460
AT5G63490
AT5G63510
AT5G63520
AT5G63570
AT5G63610
AT5G63620
AT5G63640
AT5G63650
AT5G63680
AT5G63770
AT5G63780
AT5G63810
AT5G63840
AT5G63860
AT5G63870
AT5G63890
AT5G63920
AT5G63930
AT5G63940
AT5G63960
AT5G63980
AT5G64000
AT5G64020
AT5G64030
AT5G64050
AT5G64070
AT5G64130
AT5G64160
AT5G64170
AT5G64200
AT5G64220
AT5G64240
AT5G64300
AT5G64340
AT5G64341
AT5G64342
AT5G64343
AT5G64350
AT5G64370
AT5G64390
AT5G64400
AT5G64420
AT5G64440
AT5G64460
AT5G64500
AT5G64550
AT5G64552
AT5G64560
AT5G64580
AT5G64610
AT5G64685
AT5G64730
AT5G64740
AT5G64760
AT5G64813
AT5G64820
AT5G64830
AT5G64880
AT5G64930
AT5G64960
AT5G65000
AT5G65010
AT5G65050
AT5G65060
AT5G65180
AT5G65210
AT5G65290
AT5G65440
AT5G65450
AT5G65460
AT5G65470
AT5G65540
AT5G65550
AT5G65670
AT5G65685
AT5G65687
AT5G65740
AT5G65750
AT5G65760
AT5G65770
AT5G65780
AT5G65810
AT5G65930
AT5G65950
AT5G65960
AT5G66010
AT5G66030
AT5G66050
AT5G66060
AT5G66120
AT5G66180
AT5G66210
AT5G66280
AT5G66290
AT5G66310
AT5G66410
AT5G66420

AT5G66510
AT5G66520
AT5G66600
AT5G66680
AT5G66720
AT5G66750
AT5G66760
AT5G66810
AT5G66880
AT5G66930
AT5G67030
AT5G67100
AT5G67130
AT5G67170
AT5G67240
AT5G67320
AT5G67380
AT5G67385
AT5G67500
AT5G67530
AT5G67540
AT5G67560
AT5G67580
AT5G67610
