# Supplementary material for: Primed histone demethylation regulates shoot regenerative competency
Source: Nat Commun. 2019 Apr 16;10:1786. doi: 10.1038/s41467-019-09386-5 (PMC6467990; doi:10.1038/s41467-019-09386-5)
Supplement: Supplementary file 9 — Supplementary Data 6 [file 41467_2019_9386_MOESM9_ESM.pdf]

Supplementary Data 6. Gene expression & H3K4me2 methylation levels(RPM) in LDL3 target candidate genes

	RNA-seq(RPM)						ChIP-seq(RPM)				
	**Col_C14**	**ldl3_C14**	**Col_C14S1**	**ldl3_C14S1**	**Col_C14S7**	**ldl3_C14S7**	**Col_C14_K4me2**	**ldl3_C14_K4me2**	**Col_C14S1_K4me2**	**ldl3_C14S1_K4me2**	
**AT1G18460**	33.76612508	30.15766451	47.69969756	36.12534407	39.28840563	37.52780882	40.22680802	74.45865594	36.6168201	72.77988361	alpha/beta-Hydrolases superfamily protein
**AT1G30270**	102.8721286	79.46123669	144.0898201	97.2697969	119.2796629	95.23733368	44.01487731	70.48844381	36.79860454	65.25555144	CBL-interacting protein kinase 23
**AT1G76550**	52.01135721	36.48785906	71.19985488	46.4670273	80.66234492	72.37789281	68.40171335	103.2900283	58.42231435	95.77276108	Phosphofructokinase family protein
**AT2G30600**	41.82607063	38.8639592	70.471934	45.58091477	76.46197727	55.50600002	46.71422481	91.03571656	45.26980281	93.12841294	BTB/POZ domain-containing protein
**AT2G34750**	213.4169672	171.2000019	285.1558574	214.825399	200.1620371	197.2164335	37.29393108	80.74257505	32.70741995	78.80188156	RNA polymerase I specific transcription initiation factor RRN3 protein
**AT3G02470**	445.7669499	356.523196	647.5197746	418.8443135	935.6100064	824.0563652	37.91627784	62.2553911	32.67582428	57.72253299	S-adenosylmethionine decarboxylase
**AT3G22200**	130.6000461	140.8496993	184.579169	137.8795891	114.8386831	84.2093252	42.96125635	77.14397899	45.331032	82.19764591	Pyridoxal phosphate (PLP)-dependent transferases superfamily protein
**AT3G24180**	47.28947405	46.95617916	65.50849208	46.07246704	48.26279634	42.5082483	61.02150702	135.9800516	51.58089963	136.0881553	Beta-glucosidase, GBA2 type family protein
**AT3G55610**	94.40899007	79.157906	128.3945438	91.06799721	133.8826213	113.7348207	47.92871045	90.08063331	43.85072801	92.8400558	delta 1-pyrroline-5-carboxylate synthase 2
**AT4G38470**	25.3944425	20.18517921	52.7517905	31.54443655	24.18795281	15.32848462	62.76028705	90.27121117	55.58786458	88.26555134	ACT-like protein tyrosine kinase family protein
**AT4G39850**	54.71627254	54.50026074	74.59084284	55.32295146	49.72436598	45.12302575	57.23056309	111.2819037	48.91728864	109.8064687	peroxisomal ABC transporter 1
**AT5G02880**	88.475927	81.41209461	123.5073342	90.88860237	86.51450425	77.88490443	64.27573507	116.5072313	55.44552442	115.1333826	ubiquitin-protein ligase 4
**AT5G13710**	184.6141113	130.5212232	317.3854893	232.2105942	288.4195704	257.6286891	45.23295472	64.43301624	39.95548863	60.10681405	sterol methyltransferase 1
**AT5G23380**	31.14943571	25.73978895	58.48262479	38.87657872	34.89409516	36.93728401	40.90546741	61.11186588	40.27754464	58.55211728	Protein of unknown function (DUF789)
**AT5G53460**	597.385394	479.180418	816.6936425	506.4696424	526.1238021	378.9741431	111.5137762	225.5554981	109.1524239	216.9291337	NADH-dependent glutamate synthase 1
**AT5G56890**	28.77057816	30.90673231	41.69223724	30.90551745	22.8926666	18.24230662	52.31989971	100.4572077	51.46802754	97.87364876	Protein kinase superfamily protein
