# Supplementary material for: Primed histone demethylation regulates shoot regenerative competency
Source: Nat Commun. 2019 Apr 16;10:1786. doi: 10.1038/s41467-019-09386-5 (PMC6467990; doi:10.1038/s41467-019-09386-5)
Supplement: Supplementary file 5 — Supplementary Data 2 [file 41467_2019_9386_MOESM5_ESM.pdf]

Supplemantary Data 2. Pearson correlation between biological replicates in RNA-seq and ChIP-seq experiments.

	Col_C0_H3_1	Col_C0_H3_2	Col_C0_K4me1_1	Col_C0_K4me1_2	Col_C0_K4me2_1	Col_C0_K4me2_2	Col_C0_K4me3_1	Col_C0_K4me3_2	Col_C14_H3_1	Col_C14_H3_2	Col_C14_K4me1_1
Col_C0_H3_1	1	0.995082712	0.881693797	0.881693252	0.801381058	0.832358019	0.809568166	0.790567383	0.987160837	0.990323223	0.810174653
Col_C0_H3_2	0.995082712	1	0.886439552	0.886603774	0.806744478	0.838351565	0.817111241	0.797978106	0.98644229	0.988580346	0.815450805
Col_C0_K4me1_1	0.881693797	0.886439552	1	0.987676633	0.870777958	0.905571749	0.868017526	0.851091538	0.878548643	0.878973838	0.981238997
Col_C0_K4me1_2	0.881693252	0.886603774	0.987676633	1	0.868249079	0.907450045	0.869575997	0.881298292	0.877714535	0.879193445	0.966550024
Col_C0_K4me2_1	0.801381058	0.806744478	0.870777958	0.868249079	1	0.989327125	0.912098791	0.891945331	0.781900763	0.77659732	0.797857119
Col_C0_K4me2_2	0.832358019	0.838351565	0.905571749	0.907450045	0.989327125	1	0.928729611	0.916323625	0.814927428	0.811336902	0.837788353
Col_C0_K4me3_1	0.809568166	0.817111241	0.868017526	0.869575997	0.912098791	0.928729611	1	0.969641152	0.790786879	0.791041811	0.801563762
Col_C0_K4me3_2	0.790567383	0.797978106	0.851091538	0.881298292	0.891945331	0.916323625	0.969641152	1	0.771276766	0.773947396	0.786225488
Col_C14_H3_1	0.987160837	0.98644229	0.878548643	0.877714535	0.781900763	0.814927428	0.790786879	0.771276766	1	0.990455581	0.811911729
Col_C14_H3_2	0.990323223	0.988580346	0.878973838	0.879193445	0.77659732	0.811336902	0.791041811	0.773947396	0.990455581	1	0.814313187
Col_C14_K4me1_1	0.810174653	0.815450805	0.981238997	0.966550024	0.797857119	0.837788353	0.801563762	0.786225488	0.811911729	0.814313187	1
Col_C14_K4me1_2	0.811560506	0.816546305	0.980604775	0.966589829	0.797039531	0.837243422	0.803205173	0.78889766	0.811694141	0.815732482	0.998517544
Col_C14_K4me2_1	0.517066899	0.522876559	0.589377588	0.58733533	0.860978392	0.824703133	0.67763633	0.658345326	0.49482979	0.480832747	0.512977619
Col_C14_K4me2_2	0.584214409	0.589890488	0.669704684	0.667370898	0.896957099	0.869179474	0.727990012	0.710302151	0.561761016	0.550874427	0.600313828
Col_C14_K4me3_1	0.504073348	0.514830865	0.58693533	0.587822868	0.738903941	0.734597663	0.860630889	0.832259492	0.475399204	0.476673704	0.51535201
Col_C14_K4me3_2	0.463181648	0.474141767	0.553406703	0.55809099	0.705020158	0.701117501	0.834807243	0.813344288	0.432730094	0.435682248	0.485289836
ldl3_C0_H3_1	0.992539688	0.99155776	0.869603894	0.869784115	0.785119328	0.816863848	0.794910257	0.775302475	0.992632235	0.990859808	0.797819618
ldl3_C0_H3_2	0.992453204	0.991946832	0.886051843	0.887663563	0.801880251	0.834606102	0.812603156	0.795878804	0.991378002	0.991109046	0.816238269
ldl3_C0_K4me1_1	0.849130696	0.854260801	0.992014382	0.979383122	0.849018778	0.88368942	0.82896022	0.81352394	0.848802021	0.847853513	0.988358687
ldl3_C0_K4me1_2	0.848563696	0.853505605	0.991656538	0.981393227	0.849612854	0.885539316	0.832853521	0.820440547	0.846847999	0.847924733	0.98725519
ldl3_C0_K4me2_1	0.778044404	0.786862068	0.909959228	0.903206	0.939364436	0.95113815	0.941288287	0.917580718	0.764416826	0.762056065	0.867465142
ldl3_C0_K4me2_2	0.783798036	0.792387634	0.919089235	0.913018705	0.93768387	0.952356259	0.942493199	0.920747885	0.769125211	0.768674774	0.878334857
ldl3_C0_K4me3_1	0.6870769	0.697559728	0.774598307	0.777622482	0.833154603	0.848305705	0.969037001	0.942000871	0.668840854	0.66848206	0.716764152
ldl3_C0_K4me3_2	0.67686992	0.687366941	0.770858652	0.77636617	0.833970763	0.849590713	0.967003989	0.945787688	0.655840784	0.657662047	0.712884527
ldl3_C14_H3_1	0.979231857	0.978306183	0.857333651	0.856709542	0.760523641	0.79310328	0.771466387	0.751308837	0.99326687	0.983987457	0.789326689
ldl3_C14_H3_2	0.984196971	0.982775143	0.864085635	0.87768758	0.767710799	0.804462929	0.781492592	0.786175864	0.987349965	0.987619741	0.796580858
ldl3_C14_K4me1_1	0.801666211	0.805818773	0.965902316	0.951077369	0.780945014	0.819119486	0.75660215	0.743467245	0.8043762	0.805152369	0.986489544
ldl3_C14_K4me1_2	0.808936516	0.812810538	0.968839376	0.955305552	0.789207428	0.827344688	0.76497865	0.753020879	0.810041132	0.812120226	0.986318775
ldl3_C14_K4me2_1	0.737574408	0.746095619	0.896428063	0.886229025	0.912575876	0.923281334	0.891263646	0.868520781	0.722808008	0.72208803	0.869088253
ldl3_C14_K4me2_2	0.758335749	0.766400818	0.919108782	0.910679945	0.911237889	0.926409262	0.895361775	0.876836261	0.744879677	0.745037232	0.895870648
ldl3_C14_K4me3_1	0.519500814	0.530979835	0.614833106	0.614606998	0.732717686	0.73434332	0.863116648	0.835352838	0.493425627	0.495132062	0.550111397
ldl3_C14_K4me3_2	0.516761957	0.528059797	0.618850965	0.624360738	0.729694128	0.734056314	0.86221549	0.845187897	0.489935162	0.493097367	0.556611152
Col_C14S1_K4me2_1	0.524951586	0.53037859	0.596442523	0.594221193	0.858648797	0.823766547	0.670130444	0.65152361	0.503056768	0.489234108	0.521142874
Col_C14S1_K4me2_2	0.517100537	0.522424393	0.583604964	0.581413347	0.847476683	0.811783636	0.65158092	0.633648078	0.495857768	0.481565002	0.508569515
Col_C14_K27me3_1	0.394402456	0.385722337	0.27836459	0.284485033	0.290070627	0.295267425	0.237322284	0.229816518	0.394884514	0.399053739	0.235233367
Col_C14_K27me3_2	0.400582307	0.391308331	0.290091842	0.295765715	0.303131652	0.308390724	0.252778726	0.244775483	0.399084896	0.405613617	0.246995086
ldl3_C14_K27me3_1	0.514561947	0.50607281	0.385328421	0.391036542	0.377768648	0.388103795	0.33834994	0.328128697	0.513819284	0.518519266	0.335239255
ldl3_C14_K27me3_2	0.34920484	0.339934668	0.244319947	0.250343444	0.258799562	0.263261739	0.218994498	0.211652842	0.346179695	0.354702985	0.205664851
Col_C14S1_K27me3_1	0.353567415	0.344868497	0.247609392	0.253175232	0.26498788	0.268747633	0.212644975	0.205405413	0.352371624	0.358257017	0.207866691
Col_C14S1_K27me3_2	0.503897381	0.495942311	0.38892269	0.393502023	0.397508932	0.40504103	0.347914716	0.336480433	0.501404322	0.505621492	0.338545424
ldl3_C14S1_K27me3_1	0.556168142	0.547705526	0.43308958	0.438019817	0.424578312	0.436059437	0.387780848	0.376836267	0.550361788	0.559025586	0.381554783
ldl3_C14S1_K27me3_2	0.687959832	0.679824066	0.561992055	0.566578571	0.542509164	0.558628294	0.513905133	0.500763833	0.676802761	0.687972979	0.502076275

	Col_C14_K4me1_2	Col_C14_K4me2_1	Col_C14_K4me2_2	Col_C14_K4me3_1	Col_C14_K4me3_2	ldl3_C0_H3_1	ldl3_C0_H3_2	ldl3_C0_K4me1_1	ldl3_C0_K4me1_2	ldl3_C0_K4me2_1	ldl3_C0_K4me2_2
Col_C0_H3_1	0.811560506	0.517066899	0.584214409	0.504073348	0.463181648	0.992539688	0.992453204	0.849130696	0.848563696	0.778044404	0.783798036
Col_C0_H3_2	0.816546305	0.522876559	0.589890488	0.514830865	0.474141767	0.99155776	0.991946832	0.854260801	0.853505605	0.786862068	0.792387634
Col_C0_K4me1_1	0.980604775	0.589377588	0.669704684	0.58693533	0.553406703	0.869603894	0.886051843	0.992014382	0.991656538	0.909959228	0.919089235
Col_C0_K4me1_2	0.966589829	0.58733533	0.667370898	0.587822868	0.55809099	0.869784115	0.887663563	0.979383122	0.981393227	0.903206	0.913018705
Col_C0_K4me2_1	0.797039531	0.860978392	0.896957099	0.738903941	0.705020158	0.785119328	0.801880251	0.849018778	0.849612854	0.939364436	0.93768387
Col_C0_K4me2_2	0.837243422	0.824703133	0.869179474	0.734597663	0.701117501	0.816863848	0.834606102	0.88368942	0.885539316	0.95113815	0.952356259
Col_C0_K4me3_1	0.803205173	0.67763633	0.727990012	0.860630889	0.834807243	0.794910257	0.812603156	0.82896022	0.832853521	0.941288287	0.942493199
Col_C0_K4me3_2	0.78889766	0.658345326	0.710302151	0.832259492	0.813344288	0.775302475	0.795878804	0.81352394	0.820440547	0.917580718	0.920747885
Col_C14_H3_1	0.811694141	0.49482979	0.561761016	0.475399204	0.432730094	0.992632235	0.991378002	0.848802021	0.846847999	0.764416826	0.769125211
Col_C14_H3_2	0.815732482	0.480832747	0.550874427	0.476673704	0.435682248	0.990859808	0.991109046	0.847853513	0.847924733	0.762056065	0.768674774
Col_C14_K4me1_1	0.998517544	0.512977619	0.600313828	0.51535201	0.485289836	0.797819618	0.816238269	0.988358687	0.98725519	0.867465142	0.878334857
Col_C14_K4me1_2	1	0.509494379	0.597567772	0.518562221	0.489144598	0.798650668	0.81728693	0.987004788	0.98644448	0.868018691	0.879354073
Col_C14_K4me2_1	0.509494379	1	0.989683622	0.583696579	0.553075163	0.502541688	0.516691732	0.579986351	0.577486614	0.737117974	0.726937208
Col_C14_K4me2_2	0.597567772	0.989683622	1	0.600513668	0.569559666	0.568490665	0.584323809	0.661114698	0.659634955	0.790609794	0.783479513
Col_C14_K4me3_1	0.518562221	0.583696579	0.600513668	1	0.994716241	0.48775847	0.50521513	0.545746572	0.548404892	0.782150934	0.77762145
Col_C14_K4me3_2	0.489144598	0.553075163	0.569559666	0.994716241	1	0.446213275	0.464529632	0.512430888	0.516011296	0.756976064	0.75281519
ldl3_C0_H3_1	0.798650668	0.502541688	0.568490665	0.48775847	0.446213275	1	0.993920251	0.837748805	0.836257831	0.762784446	0.767662158
ldl3_C0_H3_2	0.81728693	0.516691732	0.584323809	0.50521513	0.464529632	0.993920251	1	0.854656821	0.854325584	0.782865078	0.788542225
ldl3_C0_K4me1_1	0.987004788	0.579986351	0.661114698	0.545746572	0.512430888	0.837748805	0.854656821	1	0.996771599	0.881388386	0.891801795
ldl3_C0_K4me1_2	0.98644448	0.577486614	0.659634955	0.548404892	0.516011296	0.836257831	0.854325584	0.996771599	1	0.882947255	0.894214329
ldl3_C0_K4me2_1	0.868018691	0.737117974	0.790609794	0.782150934	0.756976064	0.762784446	0.782865078	0.881388386	0.882947255	1	0.996125715
ldl3_C0_K4me2_2	0.879354073	0.726937208	0.783479513	0.77762145	0.75281519	0.767662158	0.788542225	0.891801795	0.894214329	0.996125715	1
ldl3_C0_K4me3_1	0.718990846	0.61778016	0.660820207	0.910433847	0.893930123	0.673225199	0.692642204	0.729105224	0.733467623	0.909116236	0.906999764
ldl3_C0_K4me3_2	0.71553833	0.619719934	0.663188868	0.913244859	0.898624982	0.661139938	0.682075931	0.725471506	0.731034456	0.909043757	0.907954241
ldl3_C14_H3_1	0.788989227	0.47726004	0.54231141	0.460482925	0.41786395	0.989774688	0.986187204	0.827822328	0.824919325	0.742042544	0.74587033
ldl3_C14_H3_2	0.798027386	0.477682909	0.545883754	0.469913841	0.431624332	0.988058314	0.987317707	0.832835498	0.833009538	0.749438704	0.755498104
ldl3_C14_K4me1_1	0.984840001	0.51956385	0.605180234	0.445961817	0.412341527	0.790094995	0.80729632	0.984056196	0.98184113	0.815625146	0.827685813
ldl3_C14_K4me1_2	0.985190826	0.526095719	0.611914	0.452718965	0.419086762	0.796688945	0.814272255	0.985060997	0.983719008	0.821246403	0.833697214
ldl3_C14_K4me2_1	0.870432969	0.753510171	0.807935752	0.733861864	0.71115364	0.721124166	0.741583491	0.876551109	0.876968209	0.976884152	0.976862653
ldl3_C14_K4me2_2	0.897385651	0.731475799	0.791653787	0.719440231	0.696263941	0.741966337	0.762782926	0.901362202	0.902251116	0.976982948	0.978658077
ldl3_C14_K4me3_1	0.553444265	0.554249064	0.578640366	0.984822544	0.983810584	0.503988187	0.521865574	0.570261048	0.573069044	0.808694859	0.804468114
ldl3_C14_K4me3_2	0.56050912	0.54507818	0.571872	0.9821072	0.983764886	0.500412919	0.519511302	0.575087615	0.578962057	0.80831044	0.805311016
Col_C14S1_K4me2_1	0.51738046	0.991613527	0.986787956	0.565152062	0.534475696	0.510469084	0.524626334	0.589134986	0.586703481	0.729432351	0.720368961
Col_C14S1_K4me2_2	0.504552951	0.990959504	0.984847116	0.540706885	0.508740933	0.503068235	0.516611595	0.577023951	0.574239405	0.713559083	0.70410648
Col_C14_K27me3_1	0.23116302	0.238574251	0.252703766	0.000459106	-0.030115841	0.389763757	0.389966223	0.258048299	0.25971803	0.188247658	0.189905215
Col_C14_K27me3_2	0.243333018	0.246732249	0.262375697	0.01844868	-0.01163969	0.394709963	0.395839038	0.268998198	0.271517999	0.203382875	0.205539926
ldl3_C14_K27me3_1	0.331904198	0.278486512	0.301878511	0.078354836	0.044751488	0.510391461	0.509854198	0.360870129	0.362241516	0.286105632	0.288565237
ldl3_C14_K27me3_2	0.202655335	0.206800792	0.219273969	0.008501009	-0.018116646	0.342461581	0.344109389	0.222850412	0.225837273	0.169513631	0.171604392
Col_C14S1_K27me3_1	0.204047431	0.223987145	0.236187519	-0.009291939	-0.037929482	0.347785614	0.348655506	0.227934717	0.23007758	0.165305544	0.167099098
Col_C14S1_K27me3_2	0.334866004	0.315706219	0.337161032	0.090873032	0.056661712	0.497788231	0.49867026	0.365313179	0.366537508	0.298834784	0.300662208
ldl3_C14S1_K27me3_1	0.379111332	0.310400631	0.338422359	0.121972497	0.087897402	0.548705854	0.54982995	0.406729973	0.409058329	0.336689791	0.340204003
ldl3_C14S1_K27me3_2	0.500790227	0.384229246	0.422861345	0.223894315	0.186634936	0.678038264	0.680306925	0.531307211	0.534146838	0.462082383	0.466850178

	ldl3_C0_K4me3_1	ldl3_C0_K4me3_2	ldl3_C14_H3_1	ldl3_C14_H3_2	ldl3_C14_K4me1_1	ldl3_C14_K4me1_2	ldl3_C14_K4me2_1	ldl3_C14_K4me2_2	ldl3_C14_K4me3_1	ldl3_C14_K4me3_2	Col_C14S1_K4me2_1
Col_C0_H3_1	0.6870769	0.67686992	0.979231857	0.984196971	0.801666211	0.808936516	0.737574408	0.758335749	0.519500814	0.516761957	0.524951586
Col_C0_H3_2	0.697559728	0.687366941	0.978306183	0.982775143	0.805818773	0.812810538	0.746095619	0.766400818	0.530979835	0.528059797	0.53037859
Col_C0_K4me1_1	0.774598307	0.770858652	0.857333651	0.864085635	0.965902316	0.968839376	0.896428063	0.919108782	0.614833106	0.618850965	0.596442523
Col_C0_K4me1_2	0.777622482	0.77636617	0.856709542	0.87768758	0.951077369	0.955305552	0.886229025	0.910679945	0.614606998	0.624360738	0.594221193
Col_C0_K4me2_1	0.833154603	0.833970763	0.760523641	0.767710799	0.780945014	0.789207428	0.912575876	0.911237889	0.732717686	0.729694128	0.858648797
Col_C0_K4me2_2	0.848305705	0.849590713	0.79310328	0.804462929	0.819119486	0.827344688	0.923281334	0.926409262	0.73434332	0.734056314	0.823766547
Col_C0_K4me3_1	0.969037001	0.967003989	0.771466387	0.781492592	0.75660215	0.76497865	0.891263646	0.895361775	0.863116648	0.86221549	0.670130444
Col_C0_K4me3_2	0.942000871	0.945787688	0.751308837	0.786175864	0.743467245	0.753020879	0.868520781	0.876836261	0.835352838	0.845187897	0.65152361
Col_C14_H3_1	0.668840854	0.655840784	0.99326687	0.987349965	0.8043762	0.810041132	0.722808008	0.744879677	0.493425627	0.489935162	0.503056768
Col_C14_H3_2	0.66848206	0.657662047	0.983987457	0.987619741	0.805152369	0.812120226	0.72208803	0.745037232	0.495132062	0.493097367	0.489234108
Col_C14_K4me1_1	0.716764152	0.712884527	0.789326689	0.796580858	0.986489544	0.986318775	0.869088253	0.895870648	0.550111397	0.556611152	0.521142874
Col_C14_K4me1_2	0.718990846	0.71553833	0.788989227	0.798027386	0.984840001	0.985190826	0.870432969	0.897385651	0.553444265	0.56050912	0.51738046
Col_C14_K4me2_1	0.61778016	0.619719934	0.47726004	0.477682909	0.51956385	0.526095719	0.753510171	0.731475799	0.554249064	0.54507818	0.991613527
Col_C14_K4me2_2	0.660820207	0.663188868	0.54231141	0.545883754	0.605180234	0.611914	0.807935752	0.791653787	0.578640366	0.571872	0.986787956
Col_C14_K4me3_1	0.910433847	0.913244859	0.460482925	0.469913841	0.445961817	0.452718965	0.733861864	0.719440231	0.984822544	0.9821072	0.565152062
Col_C14_K4me3_2	0.893930123	0.898624982	0.41786395	0.431624332	0.412341527	0.419086762	0.71115364	0.696263941	0.983810584	0.983764886	0.534475696
ldl3_C0_H3_1	0.673225199	0.661139938	0.989774688	0.988058314	0.790094995	0.796688945	0.721124166	0.741966337	0.503988187	0.500412919	0.510469084
ldl3_C0_H3_2	0.692642204	0.682075931	0.986187204	0.987317707	0.80729632	0.814272255	0.741583491	0.762782926	0.521865574	0.519511302	0.524626334
ldl3_C0_K4me1_1	0.729105224	0.725471506	0.827822328	0.832835498	0.984056196	0.985060997	0.876551109	0.901362202	0.570261048	0.575087615	0.589134986
ldl3_C0_K4me1_2	0.733467623	0.731034456	0.824919325	0.833009538	0.98184113	0.983719008	0.876968209	0.902251116	0.573069044	0.578962057	0.586703481
ldl3_C0_K4me2_1	0.909116236	0.909043757	0.742042544	0.749438704	0.815625146	0.821246403	0.976884152	0.976982948	0.808694859	0.80831044	0.729432351
ldl3_C0_K4me2_2	0.906999764	0.907954241	0.74587033	0.755498104	0.827685813	0.833697214	0.976862653	0.978658077	0.804468114	0.805311016	0.720368961
ldl3_C0_K4me3_1	1	0.994824612	0.650933153	0.661162527	0.651929514	0.659075096	0.856727734	0.85408548	0.921791376	0.920769346	0.604227817
ldl3_C0_K4me3_2	0.994824612	1	0.636446753	0.650576572	0.647527794	0.655366255	0.856831061	0.854316322	0.924874364	0.924999673	0.606426295
ldl3_C14_H3_1	0.650933153	0.636446753	1	0.984728458	0.783063492	0.788219038	0.699690782	0.721222553	0.478279624	0.474026227	0.485503159
ldl3_C14_H3_2	0.661162527	0.650576572	0.984728458	1	0.788843364	0.795851089	0.708221687	0.731952388	0.487645837	0.489605068	0.48561416
ldl3_C14_K4me1_1	0.651929514	0.647527794	0.783063492	0.788843364	1	0.998399072	0.81421701	0.844657482	0.466883484	0.473290613	0.532178658
ldl3_C14_K4me1_2	0.659075096	0.655366255	0.788219038	0.795851089	0.998399072	1	0.818314997	0.848652632	0.473199653	0.479857533	0.538856606
ldl3_C14_K4me2_1	0.856727734	0.856831061	0.699690782	0.708221687	0.81421701	0.818314997	1	0.995960713	0.767373774	0.768090859	0.745051679
ldl3_C14_K4me2_2	0.85408548	0.854316322	0.721222553	0.731952388	0.844657482	0.848652632	0.995960713	1	0.753394336	0.755956845	0.724928854
ldl3_C14_K4me3_1	0.921791376	0.924874364	0.478279624	0.487645837	0.466883484	0.473199653	0.767373774	0.753394336	1	0.996427978	0.537159229
ldl3_C14_K4me3_2	0.920769346	0.924999673	0.474026227	0.489605068	0.473290613	0.479857533	0.768090859	0.755956845	0.996427978	1	0.528333933
Col_C14S1_K4me2_1	0.604227817	0.606426295	0.485503159	0.48561416	0.532178658	0.538856606	0.745051679	0.724928854	0.537159229	0.528333933	1
Col_C14S1_K4me2_2	0.583282486	0.585122203	0.478604446	0.478447069	0.522174892	0.528657908	0.731270987	0.71094533	0.51217215	0.503013792	0.995441986
Col_C14_K27me3_1	0.13384809	0.127426524	0.382716818	0.389610495	0.259919711	0.270866428	0.161125547	0.174556269	-0.006352712	-0.009057538	0.254222305
Col_C14_K27me3_2	0.150137515	0.144817209	0.38593149	0.394458298	0.27014997	0.281518739	0.175730609	0.189179063	0.011527919	0.009270372	0.262002258
ldl3_C14_K27me3_1	0.226315971	0.218267155	0.502930494	0.509576025	0.354745964	0.365590563	0.254357814	0.269703238	0.074996078	0.071967527	0.292715951
ldl3_C14_K27me3_2	0.127153104	0.122387088	0.333011922	0.343093927	0.223997004	0.23509086	0.144254536	0.156047131	0.001401725	-0.000340799	0.219087232
Col_C14S1_K27me3_1	0.115514134	0.110109132	0.339101697	0.347487846	0.231756601	0.242912947	0.139986361	0.152195238	-0.016790297	-0.019042153	0.239440539
Col_C14S1_K27me3_2	0.236998501	0.229899839	0.487803736	0.494976994	0.359547731	0.370816373	0.268617394	0.282983257	0.085752251	0.082766459	0.331171224
ldl3_C14S1_K27me3_1	0.274384872	0.267640808	0.536397241	0.547176358	0.398047922	0.409709335	0.304577036	0.320388475	0.119408185	0.117077003	0.324257497
ldl3_C14S1_K27me3_2	0.390979123	0.384477373	0.661078842	0.67486766	0.512905062	0.525309452	0.42654687	0.444199062	0.224581152	0.222548218	0.397485666

	Col_C14S1_K4me2_2	Col_C14_K27me3_1	Col_C14_K27me3_2	ldl3_C14_K27me3_1	ldl3_C14_K27me3_2	Col_C14S1_K27me3_1	Col_C14S1_K27me3_2	ldl3_C14S1_K27me3_1	ldl3_C14S1_K27me3_2
Col_C0_H3_1	0.517100537	0.394402456	0.400582307	0.514561947	0.34920484	0.353567415	0.503897381	0.556168142	0.687959832
Col_C0_H3_2	0.522424393	0.385722337	0.391308331	0.50607281	0.339934668	0.344868497	0.495942311	0.547705526	0.679824066
Col_C0_K4me1_1	0.583604964	0.27836459	0.290091842	0.385328421	0.244319947	0.247609392	0.38892269	0.43308958	0.561992055
Col_C0_K4me1_2	0.581413347	0.284485033	0.295765715	0.391036542	0.250343444	0.253175232	0.393502023	0.438019817	0.566578571
Col_C0_K4me2_1	0.847476683	0.290070627	0.303131652	0.377768648	0.258799562	0.26498788	0.397508932	0.424578312	0.542509164
Col_C0_K4me2_2	0.811783636	0.295267425	0.308390724	0.388103795	0.263261739	0.268747633	0.40504103	0.436059437	0.558628294
Col_C0_K4me3_1	0.65158092	0.237322284	0.252778726	0.33834994	0.218994498	0.212644975	0.347914716	0.387780848	0.513905133
Col_C0_K4me3_2	0.633648078	0.229816518	0.244775483	0.328128697	0.211652842	0.205405413	0.336480433	0.376836267	0.500763833
Col_C14_H3_1	0.495857768	0.394884514	0.399084896	0.513819284	0.346179695	0.352371624	0.501404322	0.550361788	0.676802761
Col_C14_H3_2	0.481565002	0.399053739	0.405613617	0.518519266	0.354702985	0.358257017	0.505621492	0.559025586	0.687972979
Col_C14_K4me1_1	0.508569515	0.235233367	0.246995086	0.335239255	0.205664851	0.207866691	0.338545424	0.381554783	0.502076275
Col_C14_K4me1_2	0.504552951	0.23116302	0.243333018	0.331904198	0.202655335	0.204047431	0.334866004	0.379111332	0.500790227
Col_C14_K4me2_1	0.990959504	0.238574251	0.246732249	0.278486512	0.206800792	0.223987145	0.315706219	0.310400631	0.384229246
Col_C14_K4me2_2	0.984847116	0.252703766	0.262375697	0.301878511	0.219273969	0.236187519	0.337161032	0.338422359	0.422861345
Col_C14_K4me3_1	0.540706885	0.000459106	0.01844868	0.078354836	0.008501009	-0.009291939	0.090873032	0.121972497	0.223894315
Col_C14_K4me3_2	0.508740933	-0.030115841	-0.01163969	0.044751488	-0.018116646	-0.037929482	0.056661712	0.087897402	0.186634936
ldl3_C0_H3_1	0.503068235	0.389763757	0.394709963	0.510391461	0.342461581	0.347785614	0.497788231	0.548705854	0.678038264
ldl3_C0_H3_2	0.516611595	0.389966223	0.395839038	0.509854198	0.344109389	0.348655506	0.49867026	0.54982995	0.680306925
ldl3_C0_K4me1_1	0.577023951	0.258048299	0.268998198	0.360870129	0.222850412	0.227934717	0.365313179	0.406729973	0.531307211
ldl3_C0_K4me1_2	0.574239405	0.25971803	0.271517999	0.362241516	0.225837273	0.23007758	0.366537508	0.409058329	0.534146838
ldl3_C0_K4me2_1	0.713559083	0.188247658	0.203382875	0.286105632	0.169513631	0.165305544	0.298834784	0.336689791	0.462082383
ldl3_C0_K4me2_2	0.70410648	0.189905215	0.205539926	0.288565237	0.171604392	0.167099098	0.300662208	0.340204003	0.466850178
ldl3_C0_K4me3_1	0.583282486	0.13384809	0.150137515	0.226315971	0.127153104	0.115514134	0.236998501	0.274384872	0.390979123
ldl3_C0_K4me3_2	0.585122203	0.127426524	0.144817209	0.218267155	0.122387088	0.110109132	0.229899839	0.267640808	0.384477373
ldl3_C14_H3_1	0.478604446	0.382716818	0.38593149	0.502930494	0.333011922	0.339101697	0.487803736	0.536397241	0.661078842
ldl3_C14_H3_2	0.478447069	0.389610495	0.394458298	0.509576025	0.343093927	0.347487846	0.494976994	0.547176358	0.67486766
ldl3_C14_K4me1_1	0.522174892	0.259919711	0.27014997	0.354745964	0.223997004	0.231756601	0.359547731	0.398047922	0.512905062
ldl3_C14_K4me1_2	0.528657908	0.270866428	0.281518739	0.365590563	0.23509086	0.242912947	0.370816373	0.409709335	0.525309452
ldl3_C14_K4me2_1	0.731270987	0.161125547	0.175730609	0.254357814	0.144254536	0.139986361	0.268617394	0.304577036	0.42654687
ldl3_C14_K4me2_2	0.71094533	0.174556269	0.189179063	0.269703238	0.156047131	0.152195238	0.282983257	0.320388475	0.444199062
ldl3_C14_K4me3_1	0.51217215	-0.006352712	0.011527919	0.074996078	0.001401725	-0.016790297	0.085752251	0.119408185	0.224581152
ldl3_C14_K4me3_2	0.503013792	-0.009057538	0.009270372	0.071967527	-0.000340799	-0.019042153	0.082766459	0.117077003	0.222548218
Col_C14S1_K4me2_1	0.995441986	0.254222305	0.262002258	0.292715951	0.219087232	0.239440539	0.331171224	0.324257497	0.397485666
Col_C14S1_K4me2_2	1	0.264854008	0.27160591	0.300810901	0.22829543	0.250043853	0.339728306	0.330879116	0.401131045
Col_C14_K27me3_1	0.264854008	1	0.989116058	0.977215517	0.978658265	0.985465007	0.97956356	0.964381418	0.917729587
Col_C14_K27me3_2	0.27160591	0.989116058	1	0.97589383	0.985278848	0.987734638	0.981615295	0.971237597	0.926474954
ldl3_C14_K27me3_1	0.300810901	0.977215517	0.97589383	1	0.968769302	0.963658295	0.980227241	0.983526982	0.959285215
ldl3_C14_K27me3_2	0.22829543	0.978658265	0.985278848	0.968769302	1	0.980532854	0.961951369	0.962060639	0.908873802
Col_C14S1_K27me3_1	0.250043853	0.985465007	0.987734638	0.963658295	0.980532854	1	0.975079039	0.958266361	0.904495129
Col_C14S1_K27me3_2	0.339728306	0.97956356	0.981615295	0.980227241	0.961951369	0.975079039	1	0.97986554	0.954857134
ldl3_C14S1_K27me3_1	0.330879116	0.964381418	0.971237597	0.983526982	0.962060639	0.958266361	0.97986554	1	0.975524844
ldl3_C14S1_K27me3_2	0.401131045	0.917729587	0.926474954	0.959285215	0.908873802	0.904495129	0.954857134	0.975524844	1
